# Reductive Metabolism of Ellagitannins in the Young Leaves of *Castanopsis sieboldii*

**DOI:** 10.3390/molecules24234279

**Published:** 2019-11-24

**Authors:** Hatsumi Wakamatsu, Sumire Tanaka, Yosuke Matsuo, Yoshinori Saito, Koyo Nishida, Takashi Tanaka

**Affiliations:** 1 Department of Natural Product Chemistry, Graduate School of Biomedical Sciences, Nagasaki University, 1-14 Bunkyo-machi, Nagasaki 852-8521, Japan; bb55316407@ms.nagasaki-u.ac.jp (H.W.); y-matsuo@nagasaki-u.ac.jp (Y.M.);; 2 Department of Pharmaceutics, Graduate School of Biomedical Sciences, Nagasaki University, 1-7-1 Sakamoto, Nagasaki 852-8501, Japan; koyo-n@nagasaki-u.ac.jp; 3 Department of Natural Product Chemistry, School of Pharmaceutical Sciences, Nagasaki University, 1-14 Bunkyo-machi, Nagasaki 852-8521, Japan; bb30114020@ms.nagasaki-u.ac.jp

**Keywords:** *Castanopsis sieboldii*, ellagitannin, dehydrohexahydroxydiphenoyl, hexahydroxydiphenoyl, triterpene

## Abstract

The leaves of *Castanopsis sieboldii* (Fagaceae) contain characteristic hexahydroxydiphenoyl (HHDP) esters of 28-*O*-glucosyl 2α,3β,23,24-tetrahydroxyolean- and urs-12-en-28-oic acids. In this study, uncharacterized substances were detected in the young leaves, which are not observed in the mature leaves. Preliminary HPLC analyses indicated that the substances had dehydro-HHDP (DHHDP) ester groups; however, the esters were unstable and decomposed during extraction. Therefore, the compounds were isolated as their stable phenazine derivatives by extracting the young leaves with acidic aqueous EtOH containing *o*-phenylenediamine. The structures of the phenazine derivatives indicated that the unstable metabolites of the young leaves were 3,24-DHHDP esters of the abovementioned triterpenes. Extraction of the young leaves with 80% acetonitrile containing reducing agents, ascorbic acid or dithiothreitol afforded the corresponding HHDP esters. Furthermore, heating of the young leaves in 80% acetonitrile also yielded the same HHDP esters as the reduction products. The results suggested that the HHDP esters are reductively produced from DHHDP esters in the young leaves. In addition, the structures of five previously reported triterpene HHDP esters were revised.

## 1. Introduction

Ellagitannins are a group of hydrolyzable tannins having hexahydroxydiphenoyl (HHDP) ester moieties and related acyl groups [1,2,3]. The HHDP group is a dimer of the galloyl group, and enzymatic conversion of gallotannins to ellagitannins has been reported [4,5,6]; however, the details of the biosynthetic mechanisms are still ambiguous. In addition, there is no chemical or biological evidence for the production mechanism of other ellagitannin acyl groups, such as dehydro-HHDP (DHHDP) and chebuloyl [1]. To clarify the biogenetical relationship between ellagitannin acyl groups, the chemical reactivity of the acyl groups and change of tannin composition during leaf growth were examined in our laboratory. Previously, it was demonstrated that the major ellagitannin in the leaves of *Camellia japonica* was oxidatively degraded as the leaves matured, and the chemical mechanism was proposed [7]. In this study, change of tannin composition in the leaves of *Castanopsis sieboldii* (syn. *C. cuspidata* var*. sieboldii*, Fagaceae) was examined. *C. sieboldii* is an evergreen broadleaf tree widely distributed in western Japan. The leaves contain galloyl shikimic acids [8] and ellagitannins, and the latter are mainly composed of a series of HHDP esters of 28-*O*-glucosyl 2α,3β,23,24-tetrahydroxyolean- and urs-12-en-28-oic acids (28-*O*-glucosyl castanopsigenins A and B) [9].

## 2. Results and Discussion

### 2.1. HPLC Analysis of the Leaves

**Figure 1:**
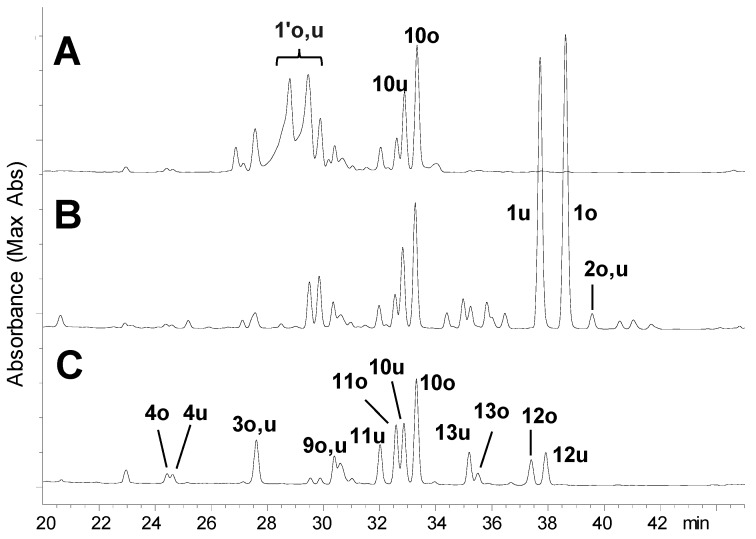
HPLC profiles of 80% CH_3_CN extracts of the leaves of *C. sieboldii* before and after derivatization. (**A**) young leaves/80% CH_3_CN 2% TFA, (**B**) young leaves/*o*-phenylenediamine in 80% CH_3_CN 2% TFA, (**C**) mature leaves/80% CH_3_CN 2% TFA. **1o**: **1** with oleanan-type triterpene unit, **1u**: **1** with ursane-type triterpene unit.

**Figure 2:**
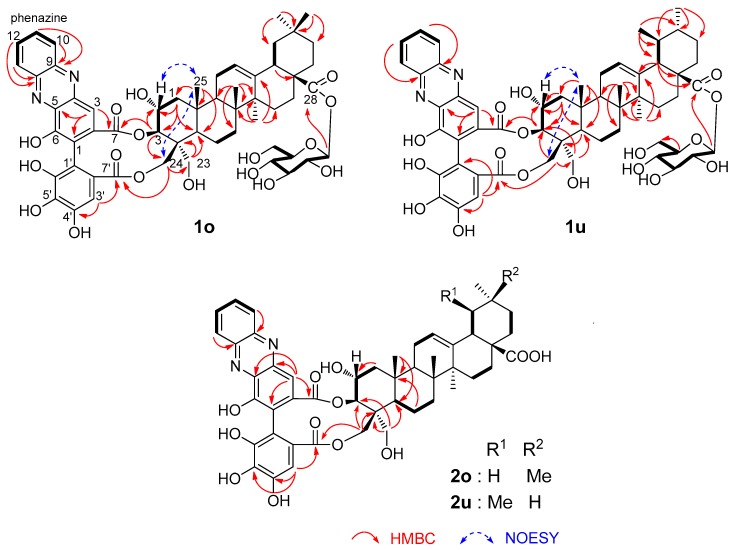
Selected ^1^H-^1^H COSY, HMBC, and NOESY correlations of **1o**, **1u**, and **2o,u**.

In the present study, firstly the triterpene HHDP esters in the fresh leaves were compared at different growth stages in spring (April, 2015). HPLC analyses of the leaf buds and small young leaves at the top of the twig showed two broad peaks with similar UV absorptions to those of other triterpene DHHDP esters (Figure 1A: **1′o,u**; where **o**: oleanane type, **u**: ursane type). However, the substances were detected neither in the wintered leaves of the same twig (Figure 1C) nor in summer mature leaves of the same tree collected in July. This observation suggests that the tannins detected as the broad peaks in the young leaves are key metabolites in the metabolism of the triterpene HHDP esters. On extraction of the young leaves with solvent containing *o*-phenylenediamine, the broad peaks were not observed and two peaks were detected at longer retention times (Figure 1B, **1o**, **1u**, and **2o,u**), suggesting that the compounds **1′o,u** have DHHDP ester moieties [10]. In contrast, no change was observed on treatment of the mature leaves with *o*-phenylenediamine. This study aimed at characterization of the key metabolites **1′o,u** in the young leaves. 

### 2.2. Structures of Phenazine Derivatives

To determine the structures of the uncharacterized metabolites **1′o,u**, the young fresh leaves were firstly extracted by a conventional method using aqueous acetone; however, the metabolites decomposed during concentration of the extract at 40–45 °C. The aforementioned preliminary experiments indicated that the unstable metabolites could be converted into stable derivatives by treatment with *o*-phenylenediamine (Figure 1B); therefore, the young leaves were extracted with 30% AcOH in EtOH containing *o*-phenylenediamine. The extract was fractionated by Diaion HP20SS column chromatography and the fractions containing triterpene esters were further separated by reversed-phase column chromatography to yield two phenazine derivatives **1o**, **1u** and an inseparable mixture of **2o,u**.

The HR-FAB-MS of **1o** and **1u** indicated that these compounds had the same molecular formula, C_56_H_66_N_2_O_17_ (**1o**: *m/z* 1061.4250 [M + Na]^+^, **1u**: *m/z* 1061.4250 [M + Na]^+^, calcd for C_56_H_66_N_2_O_17_Na, 1061.4259). Both compounds showed UV absorptions at 377, 277, and 245 nm characteristic of phenazine moieties [11], and the ^1^H- and ^13^C-NMR spectra (Table 1) showed signals arising from phenazine moieties, which were identical to those observed for the phenazine derivatives of dehydroellagitannins [12]. The ^13^C-NMR spectra also exhibited signals arising from triterpene and β-glucopyranose moieties, the chemical shifts of which were very similar to those of triterpene HHDP esters reported in a previous study [9]. Comparison of the signals with those in the literature suggested that **1o** and **1u** were esters of β-d-glucopyranosyl 2α,3β,23,24-tetrahydroxyolean-12-en-28-oate [13] and β-d-glucopyranosyl 2α,3β,23,24-tetrahydroxyurs-12-en-28-oate [14], respectively. These triterpene units were originally named as castanopsigenins A and B by Ageta et al. [9]. The location of the glucopyranose moiety at the C-28 carboxyl group was confirmed by HMBC correlations between the anomeric protons and the carboxyl carbons (Figure 2). The configurations of the A rings were determined by the appearance of NOESY correlations of the C-25 methyl protons with the H-2 and C-24 methylene protons. Furthermore, the locations of the phenazine ester moiety were determined based on the HMBC correlations of the ester carboxyl carbons with triterpene H-3 and H-24. The orientations of the acyl groups are also apparent from the HMBC correlations of the ester carbons and the aromatic protons. The atropisomerism of the biphenyl bond in **1o** and **1u** was determined to be in *S* configuration based on the appearance of positive and negative Cotton effects at 270 and 244 nm, respectively, in the CD spectrum (Figure S43 in Supplementary Materials) [15]. The absolute configurations of glucose units were determined to be d-form by acid hydrolysis and HPLC comparison with the thiazoridine derivative prepared with l-cysteine methyl ester and *o*-tolylisothiocyanate [16].

The minor phenazine derivative **2o,u** was an inseparable mixture of isomers. The ^1^H- and ^13^C-NMR spectra (Table 1) were very similar to those of **1o** and **1u**, and the HMBC correlations (Figure 2) confirmed that the partial structure including the phenazine units and triterpene A ring was the same as those of **1o** and **1u**. The ^13^C-NMR signals of the oleanan- and urusane-type triterpene moieties were also the same as those of **1o** and **1u**. Furthermore, the CD spectra displayed the same features as those of **1o** and **1u** (Figure S44). However, the signals of glucose were not observed in the NMR spectra of **2o,u**. Thus, **2o,u** were characterized to be desglucosyl analogs of **1o** and **1u** as shown in Figure 2.

### 2.3. Reaction of the Unstable Metabolites

**Figure 3:**
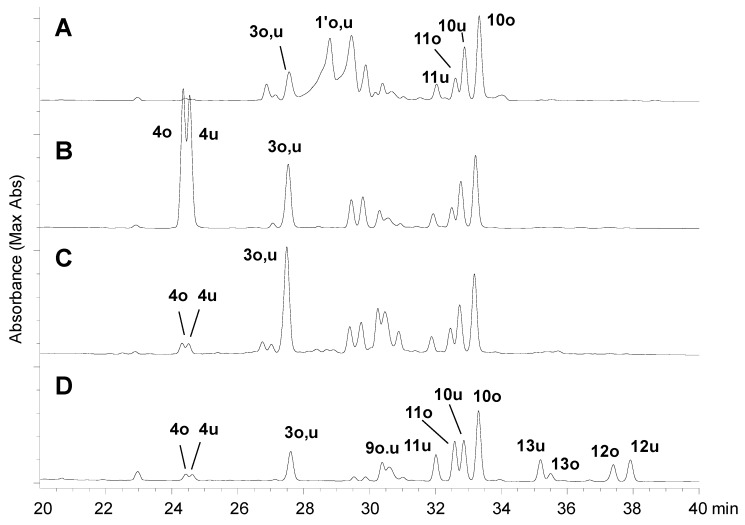
HPLC profiles of the extracts of the fresh leaves. (**A**) fresh young leaves extracted with 80% CH_3_CN containing 2% TFA. (**B**) young leaves treated with dithiothreitol in 80% CH_3_CN, r.t., 2 h. **C**: young leaves heated at 70 °C for 1 h in 80% CH_3_CN, and (**D**) mature leaves extracted with 80% CH_3_CN containing 2% TFA.

The production of these phenazine derivatives strongly suggested that the unstable compounds detected in the fresh young leaves were DHHDP esters **1′o** and **1′u** (Figure 3). It is generally accepted that the configuration of the biphenyl bond of phenazine derivatives reflects the configuration of the methine carbon of DHHDP esters [17]. Therefore, the configurations of the DHHDP group of **1′o** and **1′u** were deduced to be *S*. Most DHHDP esters of ellagitannins reported so far are stable unless the compounds were heated under acidic or basic conditions; however, Foo reported an example [18]: the 3,6-DHHDP group of 1-*O*-galloyl-2,4;3,6-bis-(*R*)-DHHDP-β-d-glucose (amariin) spontaneously degraded in EtOH to yield a reduction product having a 3,6-(*R*)-HHDP group. The degradation of **1′o** and **1′u** in the young leaves of *C. sieboldii* may be the same phenomenon. To confirm this, the young leaves were first treated with a reducing agent, dithiothreitol, in 80% CH_3_CN at r.t. for 2 h, and HPLC analysis of the reaction mixture showed **4** as a major product along with a small amount of **3**. Reduction with ascorbic acid also gave a similar result. The same products were also generated by merely heating the leaves at 70 °C in 80% CH_3_CN without any reducing agents. In this reaction, however, **3** and **4** were produced in an inverse ratio, that is **3** was the major and **4** was the minor product. Because the products **3** and **4** were detected in the extract of the mature leaves collected in October, the products were isolated from mature leaves to determine the structures.

**Scheme 1:**
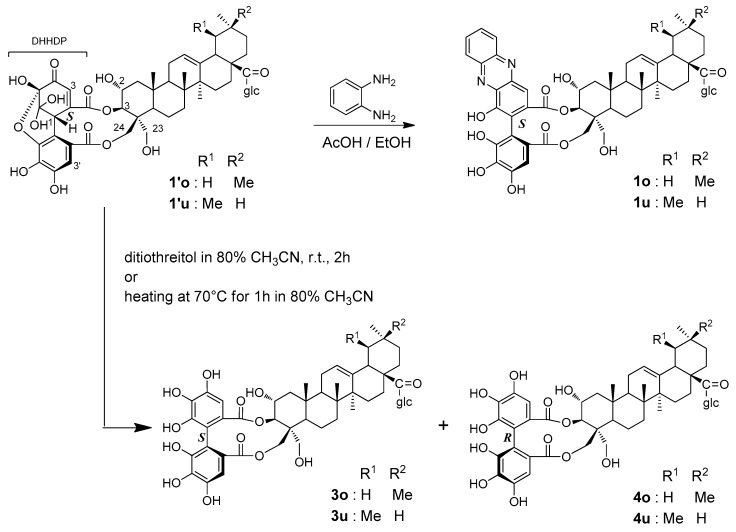
Reactions of the postulated DHHDP esters (**1′o,u**).

The product **3** was identified as the oleanane (**o**), and ursane (**u**) forms of castanopsinin E with (*S*)-HHDP ester moieties [9], and the structure was confirmed by HMBC examination (Scheme 1). Comparison of the ^13^C-NMR data of **4** with those in the literature suggested that this compound was castanopsinin C having an (*R*)-HHDP group at the triterpene 2,3-vicinal hydroxy groups [9]. This was also in agreement with the appearance of a negative Cotton effect at 231 nm. Furthermore, HPLC comparison with an authentic sample also supported that **4** was castanopsinin C. However, the HMBC spectrum of the isolated compound measured in methanol-*d*_4_ showed correlations of two ester carbonyl carbons with two doublet proton signals at δ 4.95 (d, *J* = 9.8 Hz, H-3) and δ 4.13 (d, *J* = 12.5 Hz). The coupling constant of the latter (12.5 Hz) was too large for the usual vicinal coupling between H-3 and H-2. In addition, the H-2 signal (δ 3.81, m) was not correlated with ester carbonyls in the HMBC spectrum. Therefore, it is more reasonable that the signal at δ 4.13 is attributable to geminally coupled H-23 or H-24, and one of the HHDP ester groups is attached to C-23 or C-24. Attempts to assign the signals of H-23 and H-24 by ^1^H-^1^H COSY, ROESY, HSQC, and HMBC measured in pyridine-*d*_5_ and CD_3_OD using different parameters all failed. Because of these unexpected difficulties in spectroscopic examination, DFT calculations were applied to two model compounds: 3,24- and 3,23-(*R*)-HHDP esters of methyl 2α,3β,24-trihydroxyolean-12-en-28-oate (the glucose moiety was replaced by a methyl group) (Figure S45). After a conformational search, ^1^H- and ^13^C-NMR chemical shifts of the low-energy conformers with Boltzmann populations greater than 1% were calculated [19,20]. The experimental ^1^H and ^13^C-NMR chemical shifts were in agreement with calculated values for 3,24-(*R*)-HHDP esters (*R*^2^ = 0.9802 for ^1^H; *R*^2^ = 0.9972 for ^13^C) rather than 3,23-isomers (*R*^2^ = 0.9744 for 1H; *R*^2^ = 0.9952 for ^13^C) (Figure S46). Furthermore, DP4+ analysis gave 100.0% probability for 3,24-(*R*)-HHDP esters (Table S7) [21]. Based on these results, the structure of castanopsinin C was revised to be β-d-glucopyranosyl 3,24-(*R*)-HHDP-2α,3β,23,24-tetrahydroxyurs- and olea-12-en-28-oate (**4o,u**).

**Figure 4:**
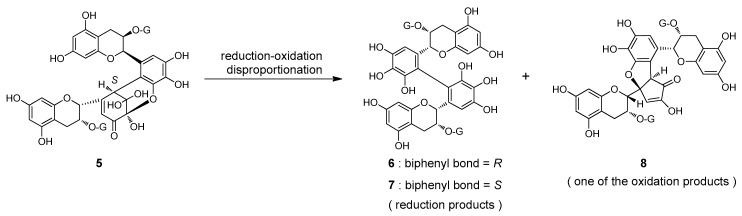
Reduction-oxidation disproportionation of dehydrotheasinensin A (**5**).

On heating in 80% CH_3_CN, the postulated DHHDP esters **1′o,u** were reduced to **3** and **4**, similar to the behavior observed for 1-*O*-galloyl-2,4;3,6-bis-(*R*)-DHHDP-β-d-glucose [18]. The most likely explanation for this phenomenon is reduction-oxidation disproportionation. A related disproportionation reaction was previously observed in black tea polyphenol production during tea fermentation. Epigallocatechin-3-*O*-gallate, a major catechin of green tea leaves, is converted to dehydrotheasinensin A (**5**), which has a hydrated quinone structure closely related to the DHHDP group. The quinone dimer **5** is unstable and decomposes on heating to give the reduction products theasinensins A (**6**) and D (**7**), accompanied by a complex mixture of oxidation products including galloyl oolongtheanin (**8**) (Figure 4) [22]. In the case of 3,6-DHHDP esters of 1-*O*-galloyl-2,4;3,6-bis-(*R*)-DHHDP-β-d-glucose, only the reduction product has been characterized in previous studies and no oxidation products have been identified. This spontaneous reduction of the hydrated quinone dimer may play a role in the metabolism of plant polyphenols to some extent.

### 2.4. Revision of Structures of Triterpene HHDP Esters

**Figure 5:**
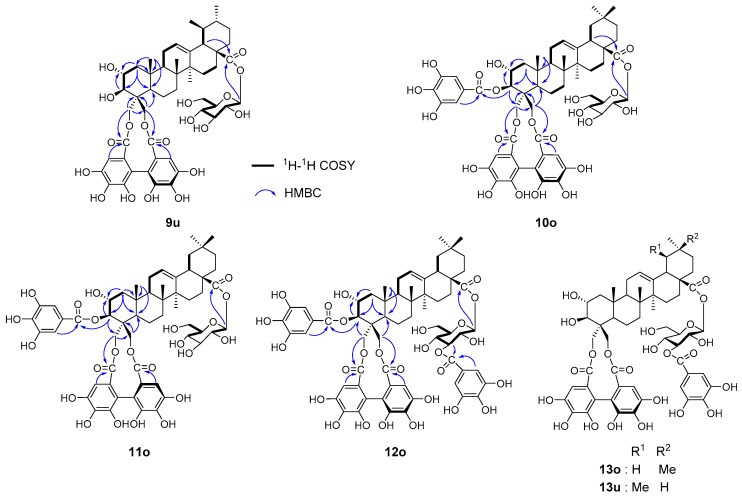
Revised structures and HMBC correlations for castanopsinins A (**9**), B (**10**), F (**11**), and H (**12**) and structure of castanopsinin G (**13**).

In this study, the structure of castanopsinin C was revised to **4** based on spectroscopic and computational methods. In the original study conducted in 1988, 2-dimensional NMR analysis was not available and the structures were mainly determined based on low-resolution 1D NMR and chemical degradation and derivatization [9]. Therefore, the major constituents of the mature leaves were isolated and reinvestigated by HMBC experiments (Figure 5).

The original structures of castanopsinins E (**3**) and G (**13**) were confirmed by HMBC experiments. However, the structure of castanopsinin A (formerly β-d-glucopyranosyl 3,23-(*R*)-HHDP-2α,3β,23,24-tetrahydroxyurs-12-en-28-oate) was revised to β-d-gluco- pyranosyl 23,24-(*R*)-HHDP-2α,3β,23,24-tetrahydroxyurs-12-en-28-oate (**9**), the structure of castanopsinin B (formerly β-d-glucopyranosyl 3,23-(*R*)-HHDP-24-*O*-galloyl-2α,3β,23,24-tetrahydroxyolean-12-en-28-oate) was revised to β-d-glucopyranosyl 3-*O*-galloyl-23,24-(*R*)-HHDP-2α,3β,23,24-tetrahydroxyolean-12-en-28-oate (**10**), the structure of castanopsinin F (formerly β-d-glucopyranosyl 3,23-(*S*)-HHDP-24-*O*-galloyl-2α,3β,23,24-tetrahydroxyolean-12-en-28-oate) was revised to β-d-glucopyranosyl 3-*O*-galloyl-23,24-(*S*)-HHDP-2α,3β,23,24-tetrahydroxyolean-12-en-28-oate (**11**), and the structure of castanopsinin H (formerly β-d-3-*O*-galloyl-glucopyranosyl 3,23-(*R*)-HHDP-24-*O*-galloyl-2α,3β,23,24-tetrahydroxyolean-12-en-28-oate) was revised to β-d-3-*O*-galloyl-glucopyranosyl 3-*O*-galloyl-23,24-(*R*)-HHDP-2α,3β,23,24-tetrahydroxyolean-12-en-28-oate (**12**). These compounds were all isolated as a mixture of oleanane- and ursane-type triterpenes, and the uniformity of the triterpene moiety of the HHDP esters was confirmed by alkaline methanolysis of a fraction containing a broad range of triterpene HHDP esters.

## 3. Materials and Methods

### 3.1. General Information

Optical rotations were measured on a JASCO P-1020 digital polarimeter (Jasco, Tokyo, Japan). IR spectra were measured on a JASCO FT/IR 410 spectrophotometer. Ultraviolet (UV) spectra were obtained on a JASCO V-560 UV/VIS spectrophotometer. ECD spectra were measured with a JASCO J-725N spectrophotometer. ^1^H- and ^13^C-NMR spectra were recorded on a Varian Unity plus 500 spectrometer (Agilent Technologies, Santa Clara, CA, USA) operating at 500 MHz and 126 MHz for the ^1^H and ^13^C nuclei, respectively. ^1^H- and ^13^C-NMR spectra were also recorded on a JEOL JNM-AL400 spectrometer (JEOL Ltd., Tokyo, Japan) operating at 400 and 100 MHz for the ^1^H and ^13^C nuclei, respectively. Coupling constants (*J*) were expressed in hertz and chemical shifts (δ) are reported in ppm with the solvent signal used as a standard (pyridine-*d*_5_: δ_H_ 7.19, δ_C_ 123.5 and methanol-*d*_4_: δ_H_ 3.31, δ_C_ 49.0). FAB-MS data were recorded on a JMS700N spectrometer (JEOL Ltd.) using *m*-nitrobenzyl alcohol or glycerol as the matrix. ESI-TOF-MS data were recorded on a Q-TOF LC/MS (Agilent 6550). Column chromatography was performed using Sephadex LH-20 (25–100 μm, GE Healthcare UK Ltd., Little Chalfont, UK), MCI-gel CHP20P (75–150 μm, Mitsubishi Chemical Co., Tokyo, Japan), Chromatorex ODS (Fuji Silysia Chemical Ltd., Kasugai, Japan), and Cosmosil 40C18-PREP (Nacalai Tesque Inc., Kyoto, Japan) columns. TLC was performed on precoated Kieselgel 60 F254 plates (0.2 mm thick, Merck, Darmstadt, Germany) with CHCl_3_–MeOH–H_2_O (10:3:0.5 or 7:3:0.5, v/v) and toluene–ethyl formate–formic acid (1:7:1, v/v) mixtures being used as the eluents. The spots were detected using UV illumination and by spraying with a 5% H_2_SO_4_ solution followed by heating. Analytical HPLC was performed on a Cosmosil 5C18-ARII (Nacalai Tesque) column (250 mm × 4.6 mm, i.d.) with a gradient elution of 4–30% (39 min) and 30–75% (15 min) CH_3_CN in 50 mM H_3_PO_4_ at 35 °C (flow rate, 0.8 mL/min; detection, Jasco photodiode array detector MD-2010) or with a gradient elution of 20–45% (40 min) and 45–80% (5 min) CH_3_CN in 50 mM H_3_PO_4_ at 35 °C (flow rate, 1 mL/min; detection, Jasco photodiode array detector MD-2010).

### 3.2. Plant Material

Fresh leaves of *C. sieboldii* were collected at Nagasaki University in Japan in April 2015 and October 2016. A voucher specimen was deposited at the Nagasaki University Graduate School of Biomedical Sciences.

### 3.3. Extraction and HPLC Analysis

The crushed young leaves of *C. sieboldii* collected at the end of April (200 mg) were extracted with 2% trifluoroacetic acid (TFA) in 80% aqueous CH_3_CN (4 mL) in a screw-capped vial and shaken at r.t. for 2 h (Figure 1A, Figure 3A). The crushed leaves (200 mg) were treated with 2% TFA in 80% aqueous CH_3_CN containing *o*-phenylenediamine (10 mg/4 mL) at r.t. for 2 h (Figure 1B). The crushed leaves (200 mg) were treated with 80% aqueous CH_3_CN containing dithiothreitol (20 mg/4 mL) at r.t. for 2 h (Figure 3B). The crushed leaves (200 mg) were heated at 70 °C for 1 h in 80% aqueous CH_3_CN (4 mL) (Figure 3C). The crushed mature leaves collected at the end of April (200 mg) were extracted with 80% aqueous CH_3_CN (4 mL) at r.t. for 2 h (Figure 1C, Figure 3D). After removal of plant debris by filtration with a membrane filter (0.45 μm), the filtrate was analyzed using reversed-phase HPLC.

### 3.4. Isolation of Phenazine Derivatives

Fresh young leaves (1.3 kg) of *C. sieboldii* collected at the end of April were crushed using a Waring blender with 30% AcOH in EtOH containing *o*-phenylenediamine (5.0 g/2.7 L) and left to stand at r.t. for 18 h. After filtration, the plant debris was extracted with 60% EtOH for 12 h. The extract was combined and concentrated by a rotary evaporator, and the resulting precipitate was removed by filtration. The filtrate was subjected to Diaion HP20SS column chromatography (8 cm i.d. × 38 cm) with 40–100% MeOH in H_2_O (20% stepwise elution from 40% to 80% each 0.5 L and 10% stepwise elution from 80% to 100% each 1.0 L) to give four fractions containing triterpene esters: Fr. 1 (19.9 g), Fr. 2 (5.57 g), Fr. 3 (2.44 g), and Fr.4 (4.89 g). Fr. 2 was separated by a Sephadex LH-20 (4 cm i.d. × 23 cm, 100% EtOH 3.0 L, 50% aqueous acetone 0.5 L) into five subfractions, Fr. 2a–2e. Fr. 2c (4.37 g) was separated by a Chromatorex ODS (4 cm i.d. × 17 cm, 70–100% MeOH in H_2_O) to give a mixture of **1o,u** (3.93 g). A part of the mixture of **1o,u** (2.48 g of 3.93 g) was separated by successive column chromatography with Chromatorex ODS (70–100% MeOH in H_2_O) and Chromatorex ODS (40–100% CH_3_CN in H_2_O) columns to give three fractions. The second fraction (1.97 g) was separated by MPLC using a Cosmosil 40C_18_-PREP (25–45% CH_3_CN in H_2_O, 10 h, 4 mL/min) to yield **1o** (38 mg), **1u** (44 mg). Fr. 4 was separated by a Sephadex LH-20 (4 cm i.d. × 23 cm) with 100% EtOH (3.0 L) and 50% acetone (0.5 L) to give **2o**,**u** (1.98 g).

#### 3.4.1. Compound **1o**

Yellow amorphous powder [α]_D_ +57.7 (*c* 0.097, MeOH); FAB-MS *m/z*: 1039 [M + H]^+^, 1061 [M + Na]^+^; HR-FAB-MS *m/z*: 1061.4250 [M + Na]^+^, (calcd for C_56_H_66_N_2_O_17_Na, 1061.4259); IR *ν*_max_ cm^−1^: 3422, 2925, 1722, 1652, 1511, 1461; UV (MeOH) λ_max_ (log *ε*): 377 (3.83), 277 (4.60), 245 (4.38); ECD (MeOH) λ_max_ (Δ*ε*): 219 (+21.0), 244 (−24.4), 270 (+28.1); ^1^H- (pyridine-*d*_5_, 500 MHz) and ^13^C-NMR (pyridine-*d*_5_, 126 MHz): Table 1.

#### 3.4.2. Compound **1**u

Yellow amorphous powder [α]_D_ +52.1 (*c* 0.10, MeOH); FAB-MS *m/z*: 1039 [M + H]^+^, 1061 [M+Na]^+^; HR-FAB-MS *m/z*: 1061.4250 [M + Na]^+^, (calcd for C_56_H_66_N_2_O_17_Na, 1061.4259); IR *ν*_max_ cm^−1^: 3420, 2925, 1728, 1647, 1516, 1467; UV (MeOH) λ_max_ (log *ε*): 377 (3.86), 277 (4.69), 243 (4.42); ECD (MeOH) λ_max_ (Δ*ε*): 220 (+22.5), 244 (−29.8), 270 (+35.4); ^1^H- (pyridine-*d*_5_, 500 MHz) and ^13^C-NMR (pyridine-*d*_5_, 126 MHz): Table 1.

#### 3.4.3. Compound **2o,u**

Tan amorphous powder [α]_D_ +33.2 (*c* 0.095, MeOH); FAB-MS *m/z*: 877 [M+H]^+^; HR-FAB-MS *m/z*: 877.3915 [M + H]^+^, (calcd for C_50_H_57_N_2_O_12_, 877.3911); IR *ν*_max_ cm^−1^: 3363, 2937, 1710, 1646, 1618, 1515, 1457; UV (MeOH) λ_max_ (log *ε*): 365 (3.89), 278 (4.63), 245 (4.52); ECD (MeOH) λ_max_ (Δ*ε*): 221 (+18.5), 244 (−21.8), 270 (+24.1); ^1^H- (pyridine-*d*_5_, 500 MHz) and ^13^C-NMR (pyridine-*d*_5_, 126 MHz): Table 1.

### 3.5. Acid Hydrolysis of Compound 1o,u

The absolute configuration of the glucose residue was determined according to the method previously reported with modifications [16]. Compound **1o,u** (0.5 mg) was hydrolyzed with 0.5 M HCl (1 mL) in a screw-capped vial at 100 °C for 2 h and neutralized with Amberlite IR 400. After drying in vacuo, the residue was dissolved in pyridine (0.1 mL) containing l-cysteine methyl ester hydrochloride (0.5 mg) and heated at 60 °C for 1 h. To the mixture, a solution of *o*-tolylisothiocyanate (0.5 mg) in pyridine (0.1 mL) was added, and the solution was heated at 60 °C for 1 h. The reaction mixture was directly analyzed by reversed-phase HPLC. The peak at *t*_R_ 16.95 min coincided with the derivative of d-glucose (d-glucose: 16.72 min; l-glucose: 15.37 min).

### 3.6. Separation of Triterpene HHDP Esters from Mature Leaves

Mature leaves collected at the middle of October (2.5 kg) were crushed using a Waring blender with 70% aqueous acetone (8.0 L, three times) and left to stand at r.t. for 18 h. After filtration the filtrate was concentrated and the resulting precipitates were removed by filtration. The aqueous filtrate was subjected to Diaion HP20SS column chromatography (8 cm i.d. × 43 cm) with H_2_O containing increasing proportions of MeOH (20% stepwise elution from 0% to 40% each 1.0 L and 10% stepwise elution from 40% to 100% each 1.0 L) to give six fractions containing phenolic compounds: Fr. 1 (40.7 g), Fr. 2 (31.2 g), Fr. 3 (9.7 g), Fr. 4 (20.9 g), Fr. 5 (4.6 g), and Fr. 6 (3.3 g). Fr. 4 containing triterpene HHDP esters was separated by a Sephadex LH-20 (4 cm i.d. × 25 cm, 100% EtOH 2.5 L, 50% acetone, 0.5 L) into seven fractions: 4-1–4-7. Fr. 4-6 was identified as **12** (300 mg). Fr. 4-2 (5.36 g) was repeatedly subjected to separation by a Chromatorex ODS (5 cm i.d. × 23 cm, 50% to 85% MeOH in H_2_O, 5% stepwise elution each 0.3 L) and a Chromatorex ODS (5 cm i.d. × 23 cm, 40–80% MeOH in H_2_O, 5% stepwise elution each 0.3L) to give **3** (408 mg) and **4** (187 mg). Fr. 4–3 [a part (4.46 g) of 5.01 g] was subjected to Diaion HP20SS column chromatography (60–100% MeOH in H_2_O, 5% stepwise, each 0.3 L) to afford three fractions: 4-3-1–4-3-3. Fr. 4-3-2 (2.21 g) was separated by a Sephadex LH-20 (0–100% MeOH in H_2_O, 10% stepwise, each 0.2 L) to give **10** (187 mg) and a fraction (149.8 mg) containing **9**, which was repeatedly separated by MPLC using a Cosmosil 40C_18_-PREP (2 cm i.d. × 30 cm, 25–45% CH_3_CN in H_2_O, 10 h, 4 mL/min) to yield **9** (49 mg). Fr. 4-3-3 (0.98 g) was separated by a Sephadex LH-20 (3 cm i.d. × 26 cm, 40–100% MeOH in H_2_O, 10% stepwise, each 0.2 L) to give **11** (82 mg). Fr. 4–5 (1.46 g) was separated by a Sephadex LH-20 (3 cm i.d. × 26 cm, 50–100% MeOH in H_2_O, 10% stepwise, each 0.2 L) to give **13** (111 mg).

#### 3.6.1. Castanopsinin E (**3**)

Yellow amorphous powder; ECD (MeOH) λ_max_ (Δ*ε*): 232 (+24.1), 257 (−10.5), 280 (+3.1); **3o**; ^1^H-NMR (pyridine-*d*_5_, 500 MHz) δ_H_: 7.24 (2H, HHDP-3, HHDP-3′), 6.29 (1H, d, *J* = 8.2 Hz, glc-1), 5.83 (1H, d, *J* = 6.6 Hz, H3), 5.49 (m, H12), 5.43 (m, H24), 4.43 (2H, m, glc-6, H23), 4.39 (1H, m, glc-6), 4.33 (1H, m, glc4), 4.32 (1H, m, H2), 4.26 (1H, t, *J* = 8.9 Hz, glc-3), 4.18 (1H, t, *J* = 8.9 Hz, glc2), 4.11 (1H, d, *J* = 11.2 Hz, H23), 4.00 (1H, m, glc-5), 4.00 (1H, d, *J* = 10.5 Hz, H24), 3.17 (1H, dd, *J* = 13.7, 5.2 Hz, H18), 2.39 (1H, m, H5), 2.34 (1H, m, H15), 2.01 (1H, m, H1), 2.00 (1H, m, H11), 1.99 (1H, m, H16), 1.94 (1H, m, H11), 1.91 (1H, m, H16), 1.85 (2H, m, H6, H9), 1.80 (1H, m, H22), 1.71 (1H, m, H22), 1.68 (1H, m, H19), 1.61 (1H, m, H1), 1.37 (1H, m, H6), 1.29 (2H, m, H7), 1.20 (1H, m, H19), 1.09 (6H, s, H26, H27), 1.07 (3H, s, H25), 1.07 (1H, m, H15), 1.04 (2H, m, H21), 0.86 (6H, s, H29, H30); ^13^C-NMR (pyridine-*d*_5_, 126 MHz) δ_C_: 176.5 (C28), 169.6, 169.8 (HHDP-7, HHDP-7′), 146.7 (HHDP-4, HHDP-4′), 146.4 (HHDP-6, HHDP-6′), 144.2 (C13), 137.3, 138.2 (HHDP-5, HHDP-5′), 126.8, 128.3 (HHDP-2,HHDP2′), 122.9 (C12), 115.2, 116.6 (HHDP-1, HHDP-1′), 108.0, 108.2 (HHDP-3, HHDP-3′), 95.8 (glc-1), 79.3 (glc-5), 78.9 (glc-3), 77.2 (C3), 74.2 (glc-2), 71.2 (glc-4), 67.6 (C2), 65.2 (C24), 64.8 (C23), 62.3 (glc-6), 47.2 (C17), 47.0 (C4), 46.7 (C1), 46.2 (C19), 48.5 (C9), 44.8 (C5), 42.4 (C14), 41.9 (C18), 40.2 (C8), 36.8 (C10), 34.0 (C21), 33.1 (C29), 33.0 (C7), 32.5 (C22), 30.8 (C20), 28.5 (C15), 26.0 (C27), 24.2 (C11), 23.7 (C30), 23.5 (C16), 18.9 (C6), 17.9 (C25), 17.5 (C26).

#### 3.6.2. Castanopsinin C (**4**)

Yellow amorphous powder [α]_D_ +13.7 (*c* 0.46, MeOH); ESI-TOF-MS *m/z*: 969 [M+H]^+^; HR-ESI-TOF-MS *m/z*: 991.3942 [M + Na]^+^, (calcd for C_50_H_64_O_19_Na, 991.3934); IR *ν*_max_ cm^−1^: 3400, 2935, 1708, 1612, 1451; UV (MeOH) λ_max_ (log *ε*): 263 sh (4.15); ECD (MeOH) λ_max_ (Δ*ε*): 231 (−20.3), 262 (+10.2), 286 (−7.2); ***4o***: ^1^H-NMR (pyridine-*d*_5_, 500 MHz) δ_H_: 7.74, 7.10 (each 1H, s, HHDP-3, HHDP-3′), 6.31 (1H, d, *J* = 8.0 Hz, glc-1), 5.82 (1H, d, *J* = 9.7 Hz, H3), 5.34 (1H, t, *J* = 3.8 Hz, H12), 4.45 (1H, m, glc-6), 4.39 (1H, m, glc-6), 4.35 (1H, t, *J* = 9.2 Hz, glc4), 4.26 (1H, t, *J* = 8.9 Hz, glc-3), 4.18 (1H, t, *J* = 8.0 Hz, glc2), 4.17 (1H, m, H2), 4.00 (1H, m, glc-5), 3.13 (dd, 13.9, 5.2, H18), 2.27 (td, 13.9, 4.7 Hz, H15), 2.04 (1H, m, H1), 1.968 (1H, m, H11), 1.971 (1H, m, H16), 1.96 (1H, m, H5), 1.89 (1H, m, H16), 1.88 (1H, m, H11), 1.73 (1H, m, H9), 1.68 (1H, m, H19), 1.66 (1H, m, H6), 1.63 (2H, m, H22), 1.59 (1H, m, H7), 1.34 (1H, m, H6), 1.30 (1H, m, H7), 1.28 (1H, m, H21), 1.18 (1H, m, H19), 1.16 (1H, m, H1), 1.16 (3H, s, H26), 1.12 (3H, s, H25), 1.09 (3H, s, H27), 1.05 (1H, m, H15), 1.04 (1H, m, H21), 0.84(6H, s, H29, H30); ^13^C-NMR (pyridine-*d*_5_, 126 MHz) δ_C_: 176.4 (C28), 168.8, 170.6 (HHDP-7, HHDP-7′), 145.9, 145.7 (HHDP-4, HHDP-4′), 147.0, 146.8 (HHDP-6, HHDP-6′), 144.0 (C13), 139.8, 137.6 (HHDP-5, HHDP-5′), 122.7 (C12), 118.4, 118.0 (HHDP-1, HHDP-1′), 112.2, 106.7 (HHDP-3, HHDP-3′), 95.7 (glc-1), 79.2 (glc-5), 79.0 (C3), 78.9 (glc-3), 74.1 (glc-2), 71.7 (glc-4), 62.0 (glc-6), 67.4 (C2), 62.3, 62.4 (C23, C24), 48.3 (C4), 48.2 (C1), 47.5 (C9), 47.0 (2C; C5, C17), 46.0 (C19), 42.2 (C14), 41.7 (C18), 39.9 (C8), 37.7 (C10), 33.9 (C21), 33.0 (2C, C22, C29), 32.7 (C7), 30.7 (C20), 28.1 (C15), 25.9 (C27), 24.0 (C11), 23.5 (C30), 23.3 (C16), 18.2 (C6), 17.7 (C26), 17.5 (C25). Due to the broadening of the ^1^H signals, two-dimensional NMR spectroscopies were measured in pyridine-*d*_5_ and methanol-*d*_4_: ^1^H-NMR (methanol-*d*_4_, 500 MHz) δ_H_:7.16, 6.57 (each 1H, s, HHDP-3, 3’), 4.95 (1H, d, *J* = 9.8 Hz, H-3), 4.13 (1H, d, *J* = 12.5 Hz, H-24), 3.81 (1H, m, H-2); ^13^C-NMR (methanol-*d*_4_,126 MHz) δ_C_: 170.9, 167.8 (HHDP-7, 7’).

#### 3.6.3. Castanopsinin A (**9**)

Yellow amorphous powder [α]_D_ +64.1 (*c* 0.102, MeOH); FAB-MS *m/z*: 991 [M+Na]^+^; HR-FAB-MS *m/z*: 991.3941 [M + Na]^+^, (calcd for C_50_H_64_O_19_Na, 991.3940); IR *v*_max_ cm^−1^: 3393, 2924, 1724, 1617, 1454; UV (MeOH) λ_max_ (log *ε*): 255 (4.19); ECD (MeOH) λ_max_ (Δ*ε*): 234 (−7.36), 258 (+4.15), 282 (+0.69), 305 (+1.40); ^1^H- (pyridine-*d*_5_, 500 MHz) and ^13^C-NMR (pyridine-*d*_5_, 126 MHz) are included in Table S1.

#### 3.6.4. Castanopsinin B (**10**)

Yellow amorphous powder [α]_D_ +34.2 (*c* 0.101, MeOH); FAB-MS *m/z*: 1121 [M + H]^+^, 1143 [M + Na]^+^; HR-FAB-MS *m/z*: 1143.4048 [M+Na]^+^, (calcd for C_57_H_68_O_23_Na, 1143.4049); IR *v*_max_ cm^−1^: 3400, 2925, 1714, 1614, 1452; UV (MeOH) λ_max_ (log *ε*): 258 (4.25), 212 (4.59); ECD (MeOH) λ_max_ (Δ*ε*): 212 (−12.2), 226 (−2.78), 237 (−11.9), 260 (3.33), 279 (−2.90), 306 (+0.77); ^1^H- (pyridine-*d*_5_, 500 MHz) and ^13^C-NMR (pyridine-*d*_5_, 126 MHz) are included in Table S1.

#### 3.6.5. Castanopsinin F (**11**)

Yellow amorphous powder [α]_D_ +135.0 (*c* 0.102, MeOH); FAB-MS *m/z*: 1121 [M+H]^+^; HR-FAB-MS *m/z*: 1121.4231 [M + H]^+^, (calcd for C_57_H_69_O_23_, 1121.4230); IR *v*_max_ cm^−1^: 3430, 2946, 1718, 1617, 1451; UV (MeOH) λ_max_ (log *ε*): 260 (4.35), 215 (4.68); ECD (MeOH) λ_max_ (Δ*ε*): 218 (+0.40), 235 (+22.4), 259 (−3.07), 280 (+9.73); ^1^H- (pyridine-*d*_5_, 500 MHz) and ^13^C-NMR (pyridine-*d*_5_, 126 MHz) are included in Table S1.

#### 3.6.6. Castanopsinin H (**12**)

Tan amorphous powder [α]_D_ +109.3 (*c* 0.1, MeOH); FAB-MS *m/z*: 1296 [M+H+Na]^2+^; HR-FAB-MS *m/z*: 1295.4161 [M+Na]^+^, (calcd for C_64_H_72_O_27_Na, 1295.4159); IR *v*_max_ cm^−1^: 3400, 2943, 1712, 1512, 1450; UV (MeOH) λ_max_ (log *ε*): 268 (4.41), 215 (4.81); ECD (MeOH) λ_max_ (Δ*ε*): 210 (−11.6), 225 (+1.61), 238 (−14.2), 261 (+13.2), 282 (+3.51), 296 (+4.60); ^1^H- (pyridine-*d*_5_, 500 MHz) and ^13^C-NMR (pyridine-*d*_5_, 126 MHz) are included in Table S1.

### 3.7. Alkaline Methanolysis of Triterpene HHDP Ester-Containing Fraction

Fraction (Fr.) 4–3 (400 mg) containing a range of triterpene HHDP esters was treated with 4% methanolic NaOMe (10 mL) at r.t. for 12 h. After concentration of the reaction mixture under reduced pressure, the residue was chromatographed over a Diaion HP20SS (3 cm × 27 cm, 0–100% MeOH in H_2_O). The fraction containing the triterpene glucoside was concentrated and the resulting white precipitate was collected by filtration to give the triterpene glycoside (14 mg), which was identified as a mixture of bellericoside [12] and β-d-glucopyranosyl 2α,3β,23, 24-tetrahydroxyurs-12-en-28-oate [13] by comparison of its ^13^C-NMR data.

### 3.8. Computational Methods

A conformational search was performed using the Monte Carlo method and the MMFF94 force field with Spartan’14 (Wavefunction, Irvine, CA, USA). The obtained low-energy conformers within 6 kcal/mol of the most stable conformer were optimized at the B3LYP/6-31G(d,p) level in pyridine (PCM). The vibrational frequencies were also calculated at the same level to confirm the stability of the conformers, and no imaginary frequencies were found. The ^1^H- and ^13^C- NMR chemical shifts of the low-energy conformers with Boltzmann populations greater than 1% were calculated using the GIAO method at the mPW1PW91/6-311+G(2d,p) level in pyridine (PCM) [19,20]. The calculated NMR chemical shifts for each conformer were averaged according to the Boltzmann distribution theory at 298 K based on their relative Gibbs free energies, and were linearly corrected for the experimental data. All DFT calculations were performed using Gaussian 16 [23]. GaussView was used to draw the three-dimensional molecular structures [24].

## 4. Conclusions

In this study, we investigated the unstable metabolites **1′o,u** in the young leaves of *C. sieboldii*, and the structures of the DHHDP esters were deduced from spectroscopic evidence obtained from the two major phenazine derivatives **1o** and **1u**. The HPLC analysis of the leaves treated with *o*-phenylene-diamine indicated production of minor phenazine derivatives other than **1o,u**, suggesting the presence of other minor DHHDP esters in the young leaves. The most important finding is the reactivity of the DHHDP esters **1′o,u**; that is, heating of the fresh young leaves in organic solvent afforded a reduction product, castanopsinin E (**3o,u**), as the main product. The concentration of this reduction product increased in the mature leaves, suggesting the reaction is related to the ellagitannin metabolism in the leaves. The reduction of **1′o,u** is probably a reduction-oxidation disproportionation similar to that observed for the dimer of 1-*O*-galloyl-2,4;3,6-bis-(*R*)-DHHDP-β-d-glucose and epigallocatechin quinone **5** (Figure 4). The reaction observed for **1′o,u** may imply that HHDP esters are produced by reduction of DHHDP esters. Although the major triterpene HHDP esters of the mature leaves, such as **10** and **11**, are already present in the young leaves, the precursors of these compounds with DHHDP esters may be highly susceptible to the reduction-oxidation disproportionation. The presence of galloyl esters **12** and **13** in the mature leaves suggested galloylation occurs in the mature leaves. Further studies on the reactions of the DHHDP esters are now in progress.

## Figures and Tables

**Table 1:** ^1^H- (500 MHz) and ^13^C- (126 MHz) NMR Data for compounds **1o**, **1u** and **2o** (δ in ppm, *J* in Hz, measured in pyridine-d_5_).

Position	1o		1u		2o *^a^*	
	^1^H	^13^C	^1^H	^13^C	^1^H	^13^C
	Triterpene					
1	2.23 (m)	47.0	2.27 (m)	47.7	2.24 (m)	47.3
	1.53 (m)		1.56 (m)		1.54 (m)	
2	4.46 (m)	67.6	4.49 (m)	67.8	4.48 (m)	67.8
3	5.96 (d, 8.3)	80.7	5.96 (d, 8.1)	80.6	5.95 (d, 8.3)	80.6
4		48.4		48.5		48.4
5	2.36 (m)	45.7	2.37 (m)	45.8	2.36 (m)	45.8
6	1.90 (m)	18.3	1.88 (m)	18.5	1.92 (m)	18.5
			1.38 (m)		1.43 (m)	
7	1.72 (m)	32.3	1.70 (m)	33.3	1.67 (m)	32.9
			1.35 (m)		1.33 (m)	
8		39.8		40.2		40.0
9	1.90 (m)	47.7	1.86 (m)	47.8	1.90 (m)	47.5
10		37.1		37.2		37.1
11	1.99 (m)	24.0	2.01 (m)	24.1	1.90 (2H, m)	23.7
12	5.44 (m)	122.6	5.46 (m)	126.0	5.44 (m)	122.4
13		143.8		138.5		144.8
14		42.1		42.7		42.3
15	1.08 (2H, m)	28.0	1.02 (2H, m)	30.0	1.12 (2H, m)	28.2
16	1.91 (2H, m)	22.7	1.99 (m)	24.6	2.00 (2H, m)	23.8
17		46.8		48.4		46.6
18	3.19 (m)	41.6	2.50 (d, 11.3)	53.4	3.26 (dd, 4.0, 13.7)	42.0
19	1.21 (m)	45.8	0.90 (m)	39.1	1.76 (m)	46.0
					1.24 (m)	
20		30.5		39.3		32.7
21	1.04 (m)	33.8	1.31 (m)	30.8	1.18 (2H, m)	34.1
22	1.23 (m)	31.9	1.86 (m)	36.8	1.93 (2H, m)	37.3
			1.71 (m)			
23	4.39 (m)	62.4	4.42 (m)	62.7	4.38 (d, 11.0)	62.5
	4.35 (m)				4.17 (d, 11.0)	
24	5.57 (d, 11.5)	65.0	5.57 (d, 11.7)	65.2	5.56 (d, 11.7)	65.1
	4.06 (m)		4.04 (d, 11.5)		4.06 (d, 11.7)	
25	1.00 (3H, s)	23.2	1.04 (3H, s)	17.0	0.98 (3H, s)	17.4
26	1.10 (3H, s)	17.3	1.14 (3H, s)	17.8	1.01 (3H, s)	16.9
27	1.14 (3H, s)	25.8	1.09 (3H, s)	23.7	1.12 (3H, s)	26.0
28		176.2		176.2		180.1
29	0.87 (3H, s)	32.9	0.90 (3H, d, 6.3)	17.3	0.91 (3H, s)	33.2
30	0.86 (3H, s)	23.4	0.86 (3H, s)	21.2	0.97 (3H, s)	23.6
	Glucose					
1	6.31 (d, 8.0)	95.6	6.25 (d, 8.1)	95.7		
2	4.19 (m)	74.0	4.19 (m)	74.1		
3	4.27 (m)	78.7	4.27 (m)	78.9		
4	4.37 (m)	70.9	4.38 (m)	71.2		
5	4.02 (m)	79.1	4.01 (m)	79.2		
6	4.45 (m)	62.0	4.20 (m)	62.3		
	Phenazine					
1		117.2		117.5		117.4
2		136.6		135.1		136.0
3	8.30(s)	118.2	8.29 (s)	118.3	8.13 (s)	118.3
4		142.3		142.4		142.4
5		136.3		136.5		136.5
6		153.3		153.5		153.4
7		169.1		169.2		169.2
9		142.1 *^b^*		142.1 *^e^*		143.9 *^h^*
10	7.70 (m)	130.4 *^c^*	7.71 (s)	130.6 *^f^*	7.72 (m)	130.6 *^i^*
11	8.12 (m)	129.7 *^d^*	8.13 (m)	129.5 *^g^*	8.06 (m)	129.8 *^j^*
12	8.17 (m)	129.3 *^d^*	8.18 (m)	129.9 *^g^*	8.12 (m)	129.4 *^j^*
13	7.70 (m)	130.3 *^c^*	7.71 (m)	130.5 *^f^*	7.71 (m)	130.4 *^i^*
14		143.9 *^b^*		144.0 *^e^*		142.0 *^h^*
1’		115.5		115.6		115.6
2’		125.0		125.2		125.5
3’	7.49 (s)	108.5	7.49 (s)	108.7	7.50 (s)	108.6
4’		147.1		147.3		147.2
5’		138.6		138.8		138.7
6’		146.8		147.0		146.7
7’		169.2		169.4		169.3

*^a^* Only the data of oreanane isomer was shown. *^b–j^* Assignments may be interchanged.

## Supplementary Materials

The following are available online, Figure S1: Correlation plots of experimental vs. calculated ^1^H NMR chemical shifts of castanopsinin C (**4**). Figures S2–S53: ^1^H and ^13^C NMR, ^1^H-^1^H COSY, HSQC, HMBC, and NOE spectra of isolated compounds. Tables S1–S4: ^1^H and ^13^C NMR data of castanopsinins A, B, F, and H.
