# Supplementary material for: Reductive Metabolism of Ellagitannins in the Young Leaves of Castanopsis sieboldii
Source: Molecules. 2019 Nov 24;24(23):4279. doi: 10.3390/molecules24234279 (PMC6930603; doi:10.3390/molecules24234279)
Supplement: Supplementary file 1 [file molecules-24-04279-s001.pdf]

Supplementary Materials

Reductive Metabolism of Ellagitannins in the Young Leaves of *Castanopsis sieboldii*

Hatsumi Wakamatsu[1,2], Sumire Tanaka[3], Yosuke Matsuo[1], Yoshinori Saito[1], Koyo Nishida[2], and Takashi Tanaka[1,*]

1 Department of Natural Product Chemistry, Graduate School of Biomedical Sciences, Nagasaki University,
1-14 Bunkyo-machi, Nagasaki 852-8521, Japan; E-Mails: bb55316407@ms.nagasaki-u.ac.jp (H.W.); y-matsuo@nagasaki-u.ac.jp (Y.M.); saiyoshi@nagasaki-u.ac.jp (Y.S.)

2 Department of Pharmaceutics, Graduate School of Biomedical Sciences, Nagasaki University, 1-7-1 Sakamoto, Nagasaki 852-8501, Japan; E-Mail: koyo-n@nagasaki-u.ac.jp (K.N.)

3 Department of Natural Product Chemistry, School of Pharmaceutical Sciences, Nagasaki University, 1-14 Bunkyo-machi, Nagasaki 852-8521, Japan; E-Mail: bb30114020@ms.nagasaki-u.ac.jp (S.T.)

page

1 Table S1. ^{1}H (500 MHz) and ^{13}C (126 MHz) NMR Data for **9u** and **10o** (δ in ppm, J in Hz, Measured in pyridine-*d5*)

2 Table S2. ^{1}H (500 MHz) and ^{13}C (126 MHz) NMR Data for **11o** and **12o** (δ in ppm, J in Hz, Measured in pyridine-*d5*)

3 Figure S1. ^{1}H NMR spectrum of **1o** (pyridine-d_5, 500 MHz)

4 Figure S2. ^{13}C NMR spectrum of **1o** (pyridine-d_5, 126 MHz)

5 Figure S3. ^{1}H-^{1}H COSY spectrum of **1o** (pyridine-d_5, 500 MHz)

6 Figure S4. HSQC spectrum of **1o** (pyridine-d_5)

7 Figure S5. HMBC spectrum of **1o** (pyridine-d_5)

8 Figure S6. ^{1}H NMR spectrum of **1u** (pyridine-d_5, 500 MHz)

9 Figure S7. ^{13}C NMR spectrum of **1u** (pyridine-d_5, 126 MHz)

10 Figure S8. ^{1}H-^{1}H COSY spectrum of **1u** (pyridine-d_5, 500 MHz)

11 Figure S9. HSQC spectrum of **1u** (pyridine-d_5)

12 Figure S10. HMBC spectrum of **1u** (pyridine-d_5)

13 Figure S11. NOESY spectrum of **1o**,**u** (pyridine-d_5, 500 MHz)

14 Figure S12. ^{1}H NMR spectrum of **2** (pyridine-d_5, 500 MHz)

15 Figure S13. ^{13}C NMR spectrum of **2** (pyridine-d_5, 126 MHz)

16 Figure S14. ^{1}H-^{1}H COSY spectrum of **2o** (pyridine-d_5, 500 MHz)

17 Figure S15. HSQC spectrum of **2** (pyridine-d_5)

18 Figure S16. HMBC spectrum of **2** (pyridine-d_5)

19 Figure S17. ^{1}H NMR spectrum of **4** (pyridine-d_5, 500 MHz)

20 Figure S18. ^{13}C NMR spectrum of **4** (pyridine-d_5, 126 MHz)

21 Figure S19. ^{1}H-^{1}H COSY spectrum of **4** (pyridine-d_5, 500 MHz)
22 Figure S20. HSQC spectrum of **4** (pyridine-d_5)
23 Figure S21. HMBC spectrum of **4** (pyridine-d_5)
24 Figure S22. HMBC spectrum of **4** (CD_3OD)
25 Figure S23. ^{1}H NMR spectrum of **9** (pyridine-d_5, 500 MHz)
26 Figure S24. ^{13}C NMR spectrum of **9** (pyridine-d_5, 126 MHz)
27 Figure S25. ^{1}H-^{1}H COSY spectrum of **9** (pyridine-d_5, 500 MHz)
28 Figure S26. HSQC spectrum of **9** (pyridine-d_5)
29 Figure S27. HMBC spectrum of **9** (pyridine-d_5)
30 Figure S28. ^{1}H NMR spectrum of **10** (pyridine-d_5, 500 MHz)
31 Figure S29. ^{13}C NMR spectrum of **10** (pyridine-d_5, 126 MHz)
32 Figure S30. ^{1}H-^{1}H COSY spectrum of **10** (pyridine-d_5, 500 MHz)
33 Figure S31. HSQC spectrum of **10** (pyridine-d_5)
34 Figure S32. HMBC spectrum of **10** (pyridine-d_5)
35 Figure S33. ^{1}H NMR spectrum of **11** (pyridine-d_5, 500 MHz)
36 Figure S34. ^{13}C NMR spectrum of **11** (pyridine-d_5, 126 MHz)
37 Figure S35. ^{1}H-^{1}H COSY spectrum of **11** (pyridine-d_5, 500 MHz)
38 Figure S36. HSQC spectrum of **11** (pyridine-d_5)
39 Figure S37. HMBC spectrum of **11** (pyridine-d_5)
40 Figure S38. ^{1}H NMR spectrum of **12** (pyridine-d_5, 500 MHz)
41 Figure S39. ^{13}C NMR spectrum of **12** (pyridine-d_5, 126 MHz)
42 Figure S40. ^{1}H-^{1}H COSY spectrum of **12** (pyridine-d_5, 500 MHz)
43 Figure S41. HSQC spectrum of **12** (pyridine-d_5)
44 Figure S42. HMBC spectrum of **12** (pyridine-d_5)
45 Figure S43. Experimental ECD spectra of **1o** and **1u**
45 Figure S44. Experimental ECD spectra of **2o**,**u**
46 Figure S45. Lowest-energy conformers of 3,24-(*R*)-HHDP ester of methyl 2α,3β,23,24-tetrahydroxyolean-12-en-28-oate (**4'oA**) and 3,23-(*R*)-HHDP ester of methyl 2α,3β,23,24-tetrahydroxyolean-12-en-28-oate (**4'oB**) at the B3LYP/6-31G(d,p) level in pyridine.
47 Table S3. Calculated ^{1}H NMR chemical shifts of **4'oA**.
48 Table S4. Calculated ^{1}H NMR chemical shifts of **4'oB**.
49 Table S5. Calculated ^{13}C NMR chemical shifts of **4'oA**.
50 Table S6. Calculated ^{13}C NMR chemical shifts of **4'oB**.
51 Figure S46. Correlation plots of experimental ^{1}H NMR chemical shifts versus corresponding calculated ^{1}H NMR chemical shifts of **4'o**.

51 Figure S47. Correlation plots of experimental ^{13}C NMR chemical shifts versus corresponding calculated ^{13}C NMR chemical shifts of **4'o**.

52 Table S7. The DP4+ probability analysis of **4'o**.

53 Cartesian coordinates of the lowest-energy conformer of **4'oA** (**4'oA-1**) at the B3LYP/6-31G(d,p) level in pyridine (PCM)

54 Cartesian coordinates of the lowest-energy conformer of **4'oB** (**4'oB-1**) at the B3LYP/6-31G(d,p) level in pyridine (PCM).

Table S1. ^{1}H (500 MHz) and ^{13}C (125 MHz) NMR Data for **9u** and **10o** (δ in ppm, J in Hz, Measured in pyridine-$d5$)

	position	9u ^{1}H	9u ^{13}C	10o ^{1}H	10o ^{13}C
triterpene	1	2.21 (dd, 12.7, 4.9)	47.5	2.27 (dd, 13.0, 4.6)	48.0
		1.25 (m)		1.37 (m)	
	2	4.23 (m)	68.3	4.47 (m)	66.5
	3	3.42 (d, 9.0)	78.6	5.40 (d, 9.5)	79.1
	4		48.9		48.6
	5	1.41 (m)	57.40	1.54 (m)	57.3
	6	2.45 (m)	20.0	2.46 (d, 13.2)	19.9
		1.62 (m)		1.66 (m)	
	7	1.57 (m)	33.05	1.50 (m)	32.5
		1.25 (m)		1.24 (m)	
	8		39.7		39.4
	9	1.70 (m)	47.6	1.76 (m)	47.6
	10		38.1		38.2
	11	1.95 (m)	24.0	1.95 (2H, m)	24.1
	12	5.37 (m)	125.8	5.37 (t, 3.4)	122.5
	13		138.6		144.3
	14		42.4		42.0
	15	2.41 (m)	28.7	2.34 (td,13.9, 4.6)	28.4
		1.09 (m)		1.09 (m)	
	16	2.03 (m)	24.6	2.06 (td, 13.7, 3.8)	23.3
				2.00 (m)	
	17		48.3		46.9
	18	2.49 (d, 11.0)	53.1	3.18 (dd, 13.7, 5.1)	41.6
	19	1.38 (m)	39.3	1.72 (m)	46.1
				1.21 (m)	
	20	0.88 (m)	39.1		30.8
	21	1.34 (m)	30.8	1.37 (m)	34.0
				1.09 (m)	
	22	1.92 (m)	36.8	1.77 (2H, m)	32.6
		1.69 (m)			
	23	6.04 (d, 11.2)	71.4	5.54 (d, 11.5)	69.7
		3.73 (d, 11.2)		3.82 (d, 11.5)	
	24	5.31 (d, 11.2)	62.6	5.46 (d, 11.7)	61.8
		4.40 (m)		4.33 (d, 11.5)	
	25	0.87 (3H, s)	16.7	0.96 (3H, s)	16.5
	26	1.05 (3H, s)	17.5	1.02 (3H, s)	17.3
	27	1.12 (3H, s)	23.8	1.30 (3H, s)	26.2
	28		176.2		176.4
	29	0.92 (d, 6.4)	17.4	0.91 (3H, s)	33.1
	30	0.90 (d, 7.1)	21.3	0.89 (3H, s)	23.6
glucose	1	6.26 (d, 8.1)	95.8	6.34 (d, 8.3)	95.9
	2	4.20 (t, 8.3)	74.2	4.21 (t, 8.3)	74.3
	3	4.28 (t, 8.9)	78.9	4.28 (t, 8.8)	79.0
	4	4.36 (m)	71.1	4.37 (t, 9.0)	71.0
	5	4.02 (m)	79.3	4.03 (m)	79.5
	6	4.44 (m)	62.20	4.47 (m)	62.2
				4.42 (dd, 11.7, 4.5)	
HHDP	1,1'		116.5 (2C)		116.7 (2C)
	2,2'		127.5, 127.8		127.4, 126.8
	3,3'	7.16 (s), 7.29 (s)	107.3, 108.36	7.08 (s), 7.56 (s)	107.5, 108.6
	4,4'		146.8, 146.6		146.8, 145.91
	5,5'		137.8, 137.7		137.8 (2C)
	6,6'		146.19, 146.24		145.94 (2C)
	7,7'		169.6, 169.8		169.0, 169.6
galloyl	1				121.4
	2,6			8.03 (s)	111.9
	3,5				146.3
	4				141.6
	CO				167.4
	1'				
	2',6'				
	3',5'				
	4'				
	CO				

Table S2. ^{1}H (500 MHz) and ^{13}C (125 MHz) NMR Data for **11o** and **12o** (δ in ppm, J in Hz, Measured in pyridine-$d5$)

		**11o**		**12o**	
	position	^{1}H	^{13}C	^{1}H	^{13}C
triterpene	1	2.23 (dd, 4.5 ,12.8)	47.82	2.28 (m)	48.4
		1.37 (m)		1.36 (m)	
	2	4.12 (m)	65.3	4.47 (m)	66.5
	3	5.74 (d, 10.3)	84.8	5.38 (m)	79.0
	4		47.80		48.4
	5	1.18 (m)	50.8	1.52 (m)	57.5
	6	2.04 (m)	20.7	2.40 (m)	19.9
		1.75 (m)		1.59 (m)	
	7	1.22 (m)	33.3	1.52 (m)	32.7
		1.01 (m)		1.17 (m)	
	8		39.9		39.4
	9	1.75 (m)	48.5	1.73 (m)	47.6
	10		38.8		38.0
	11	1.93 (2H, m)	23.9	1.95 (m)	24.0
	12	5.40 (m)	122.3	5.37 (m)	122.6
	13		144.2		143.9
	14		42.2		42.2
	15	2.17 (m)	28.1	2.18 (m)	28.2
		1.08 (m)		1.16 (m)	
	16	2.02 (m)	23.5	2.08 (m)	24.6
		1.91 (m)			
	17		47.0		47.0
	18	3.15 (dd, 4.9, 13.9)	41.7	3.16 (dd, 13.6, 5.3)	41.7
	19	1.68 (m)	46.2	1.73 (m)	46.1
		1.19 (m)		1.22 (m)	
	20		30.7		30.6
	21	1.33 (m)	34.0	1.37 (m)	34.0
		1.05 (m)		1.11 (m)	
	22	1.82 (m)	32.5	1.81 (m)	32.6
		1.75 (m)		1.76 (m)	
	23	5.51 (d, 11.2)	69.6	5.54 (d, 11.5)	69.7
		4.15 (d, 11.0)		3.78 (d, 11.5)	
	24	5.48 (m)	63.1	5.41 (m)	61.8
		4.48 (m)		4.32 (m)	
	25	1.12 (3H, s)	17.7	0.94 (3H,s)	16.7
	26	1.05 (3H, s)	17.5	0.96 (3H, s)	17.3
	27	1.14 (3H, s)	26.0	1.21 (3H, s)	26.0
	28		176.4		176.3
	29	0.87 (3H, s)	33.1	0.91 (3H, s)	33.1
	30	0.84 (3H, s)	23.59	0.88 (3H, s)	23.6
glucose	1	6.32 (d, 7.8)	95.82	6.39 (d, 8.3)	95.4
	2	4.22 (t, 8.4)	74.14	4.30 (m)	72.3
	3	4.27 (t, 8.8)	78.91	6.15 (t, 9.3)	80.10
	4	4.37 (t, 9.0)	71.1	4.55 (t, 9.3)	68.90
	5	4.06 (m)	79.4	4.04 (m)	79.1
	6	4.42 (m)	62.3	4.41 (dd, 3.2, 6.8)	61.7
		4.47 (m)			
HHDP	1,1'		116.3, 116.56		116.7 (2C)
	2,2'		126.6, 127.2		127.4, 126.8
	3,3'	7.14 (s), 6.90 (s)	106.7, 108.0	7.07 (s), 7.53 (s)	107.6, 108.6
	4,4'		146.7, 146.5		146.8, 145.9
	5,5'		137.57, 137.8		137.8, 137.9
	6,6'		146.0, 146.4		146.0 (2C)
	7,7'		169.9, 169.8		169.00, 169.5
galloyl	1		121.74		121.3
	2,6	7.93 (s)	110.4	8.01 (s)	111.9
	3,5		147.4		146.5
	4		140.8		141.5
	CO		168.0		167.3
	1'				121.6
	2',6'			7.86 (s)	110.5
	3',5'				147.4
	4'				140.7
	CO				167.2

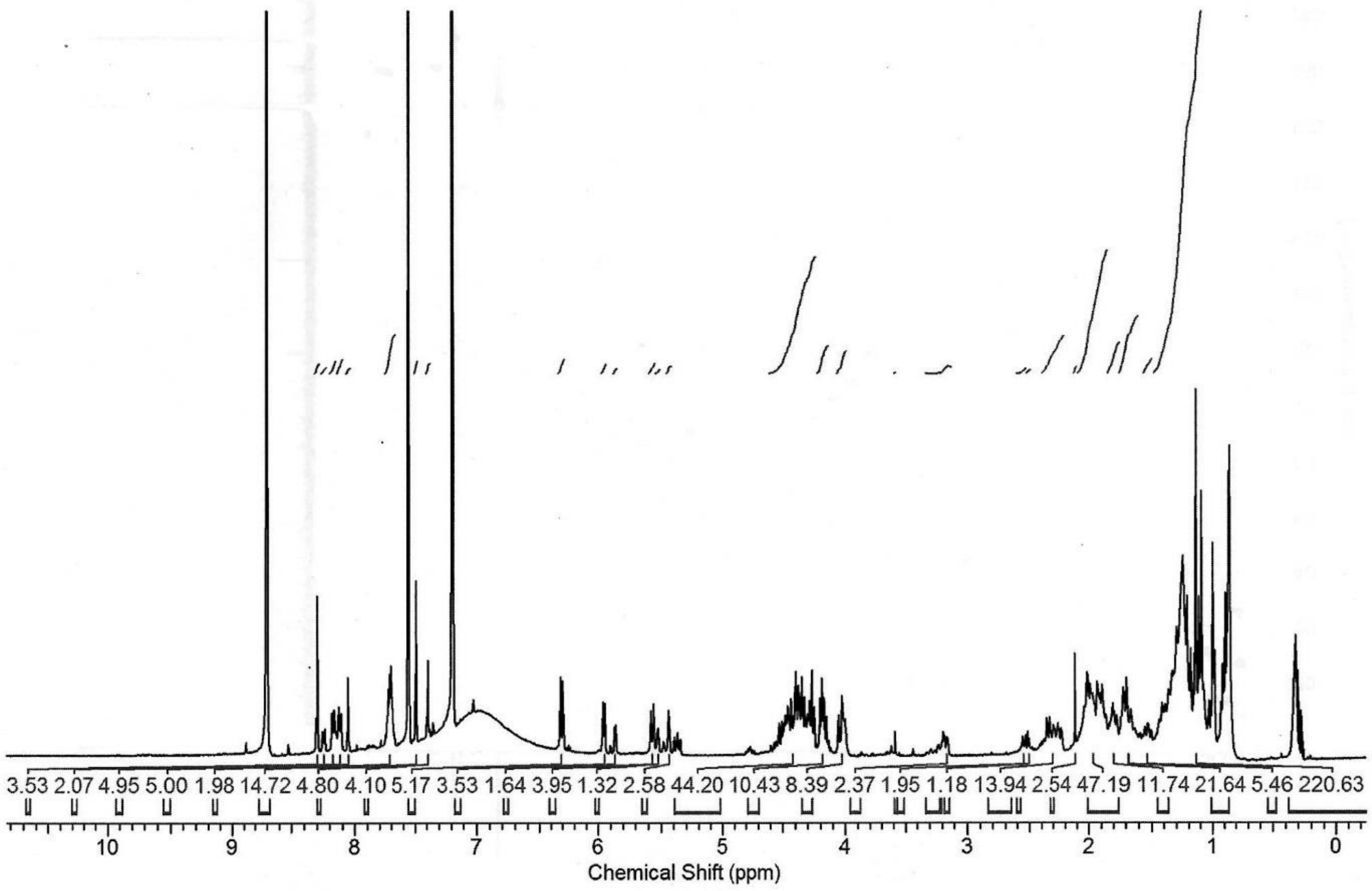

Figure S1. ^{1}H NMR spectrum of **1o** (pyridine-d_5, 500 MHz)

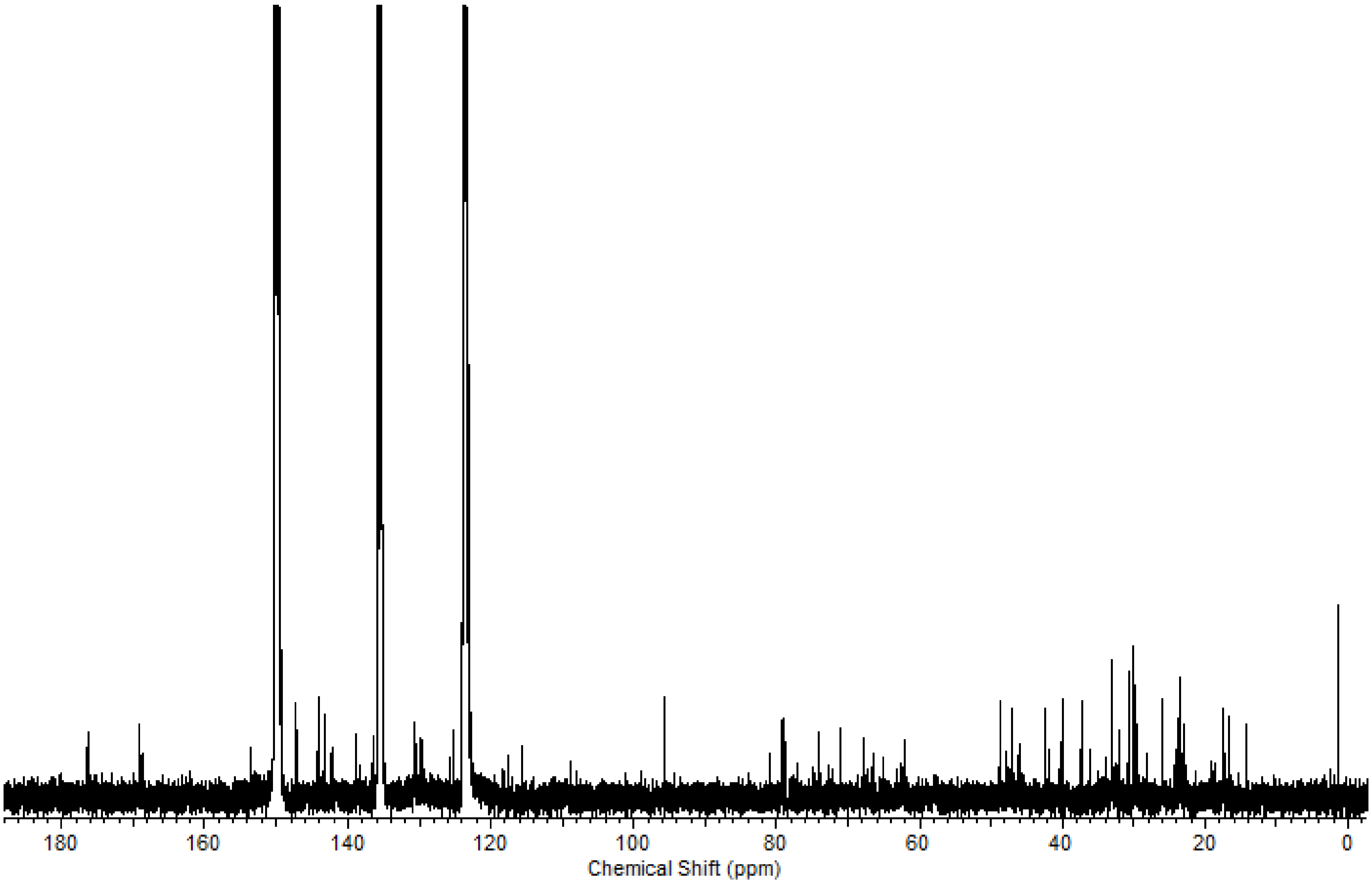

Figure S2. ^{13}C NMR spectrum of **1o** (pyridine-d_5, 126 MHz)

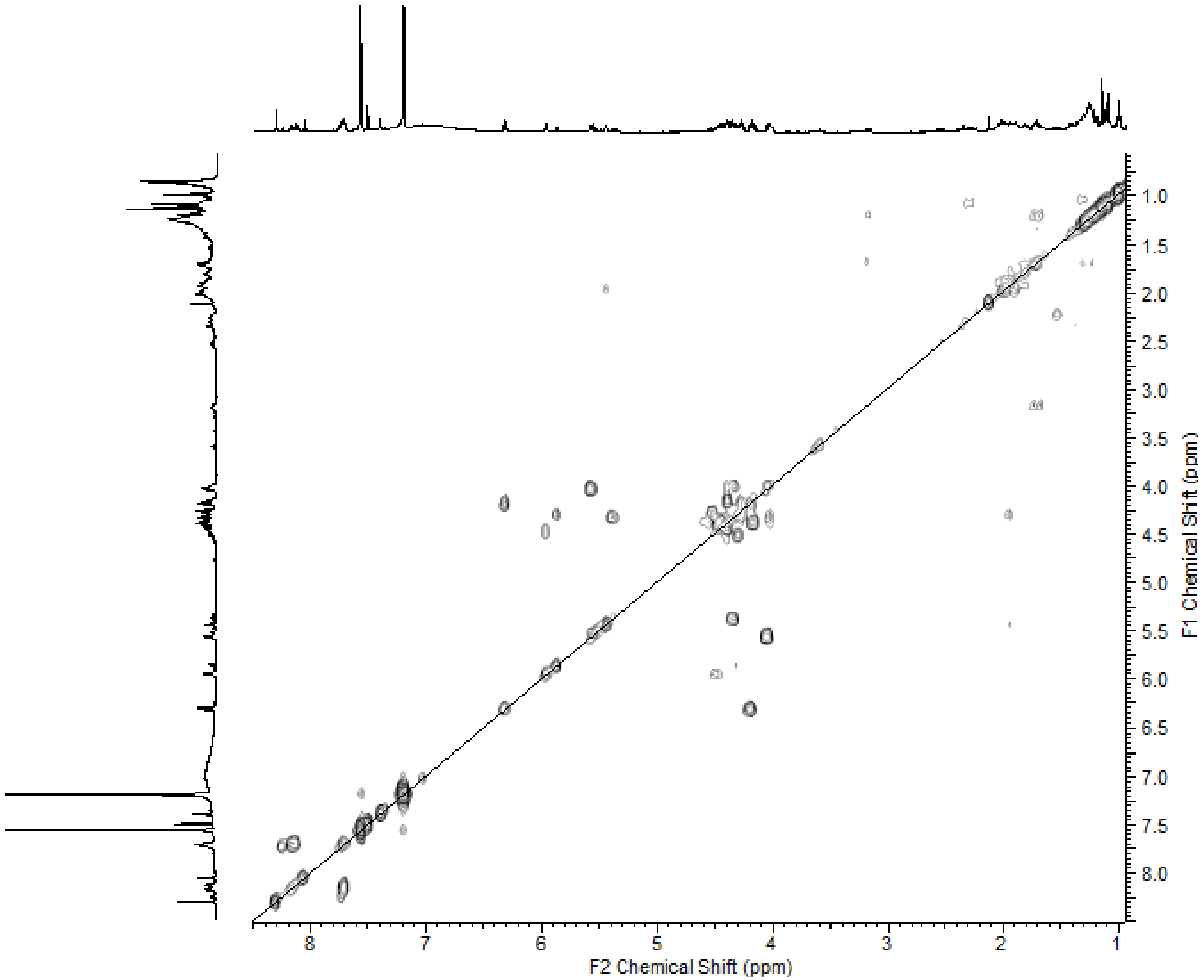

Figure S3. ^{1}H-^{1}H COSY spectrum of **1o** (pyridine-d_5, 500 MHz)

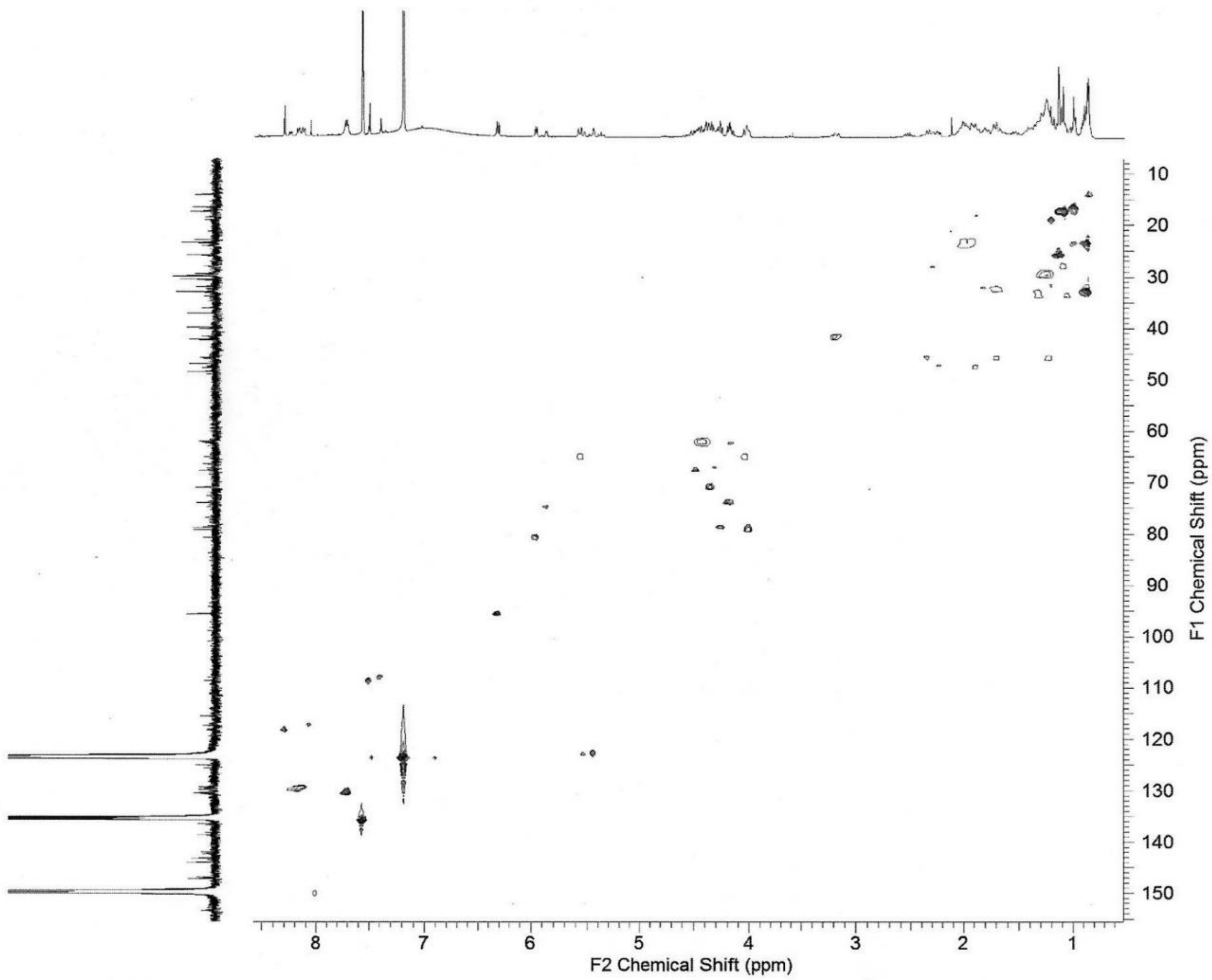

Figure S4. HSQC spectrum of **1o** (pyridine-d_5)

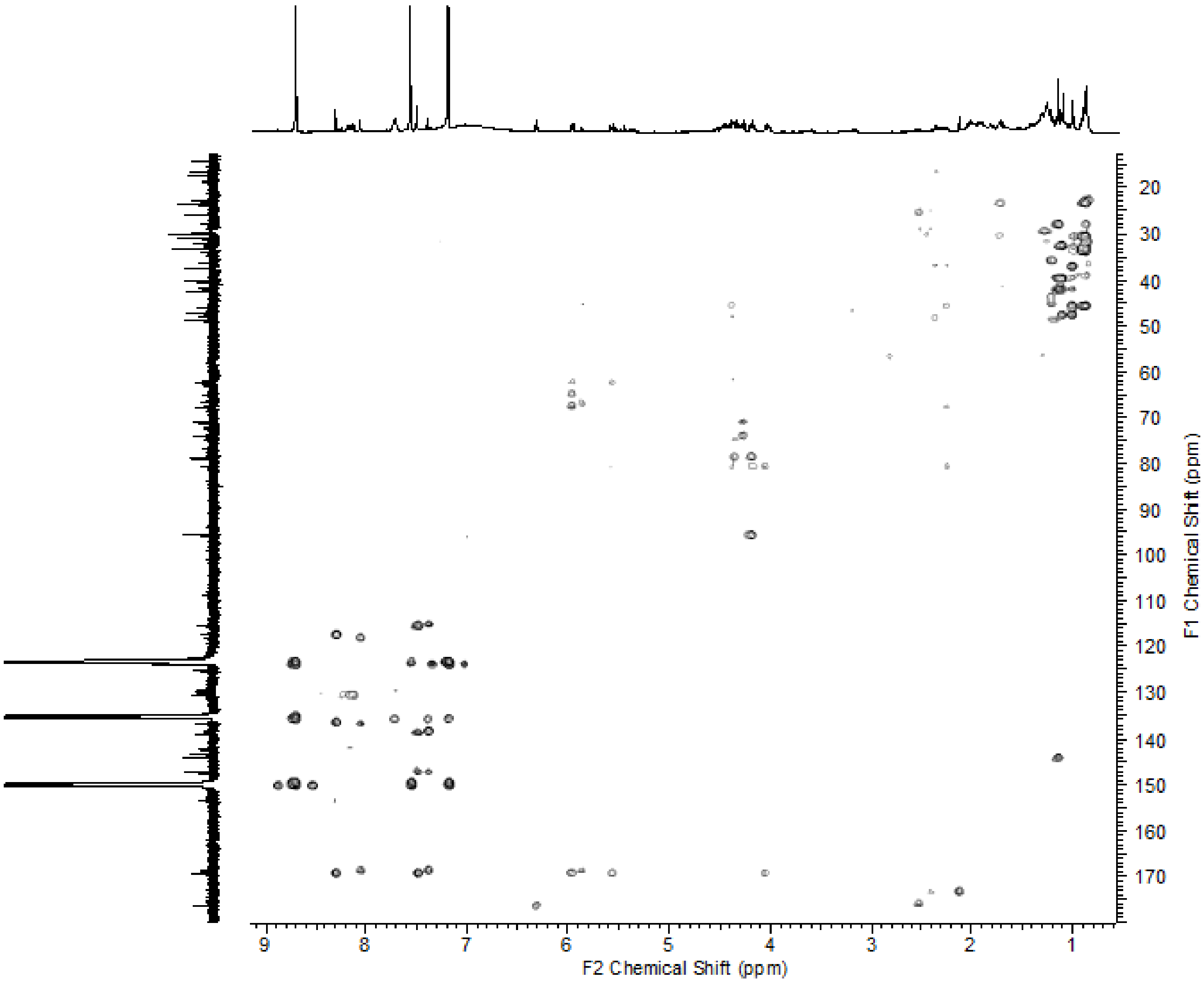

Figure S5. HMBC spectrum of **1o** (pyridine-d_5)

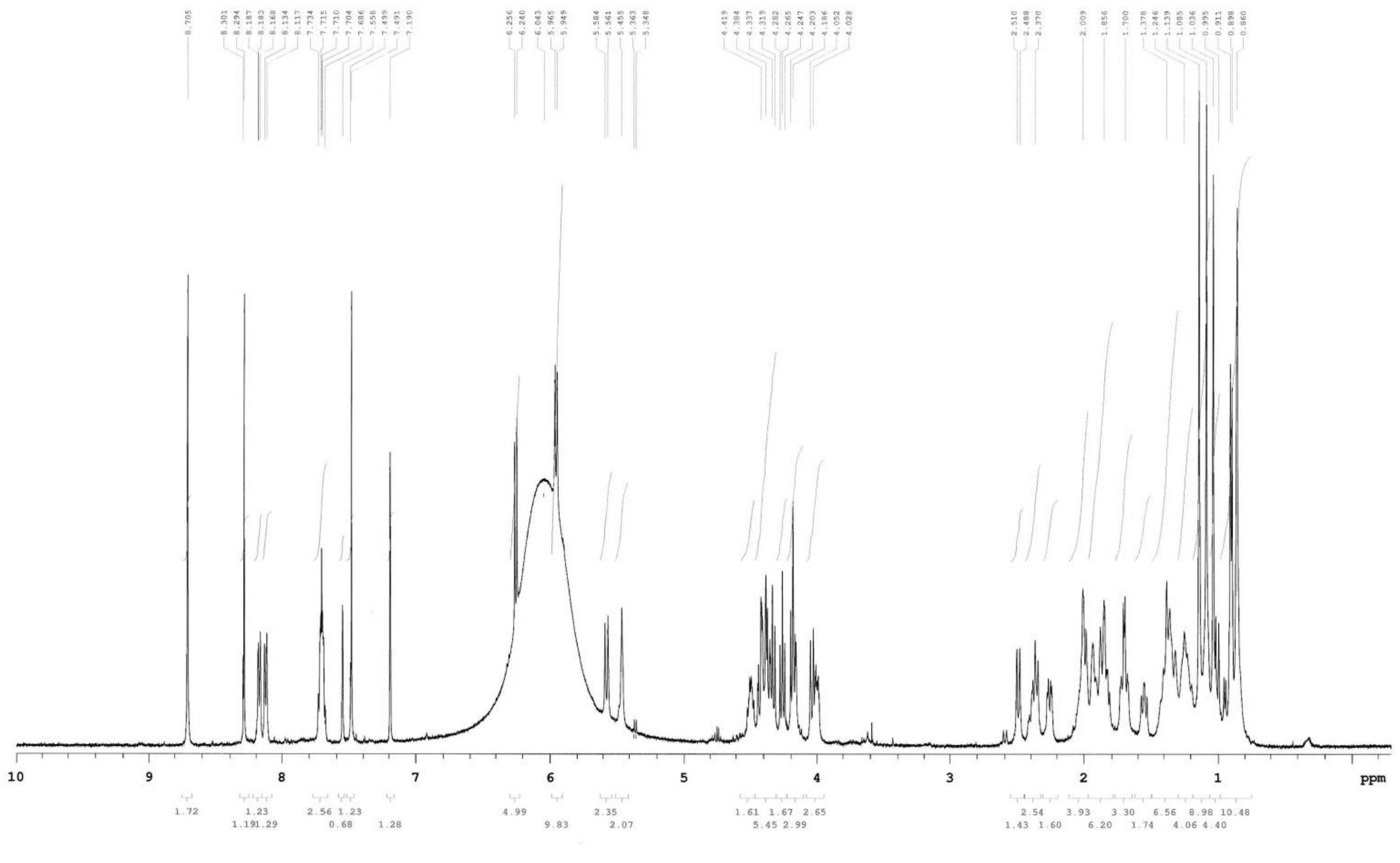

Figure S6. ^{1}H NMR spectrum of **1u** (pyridine-d_5, 500 MHz)

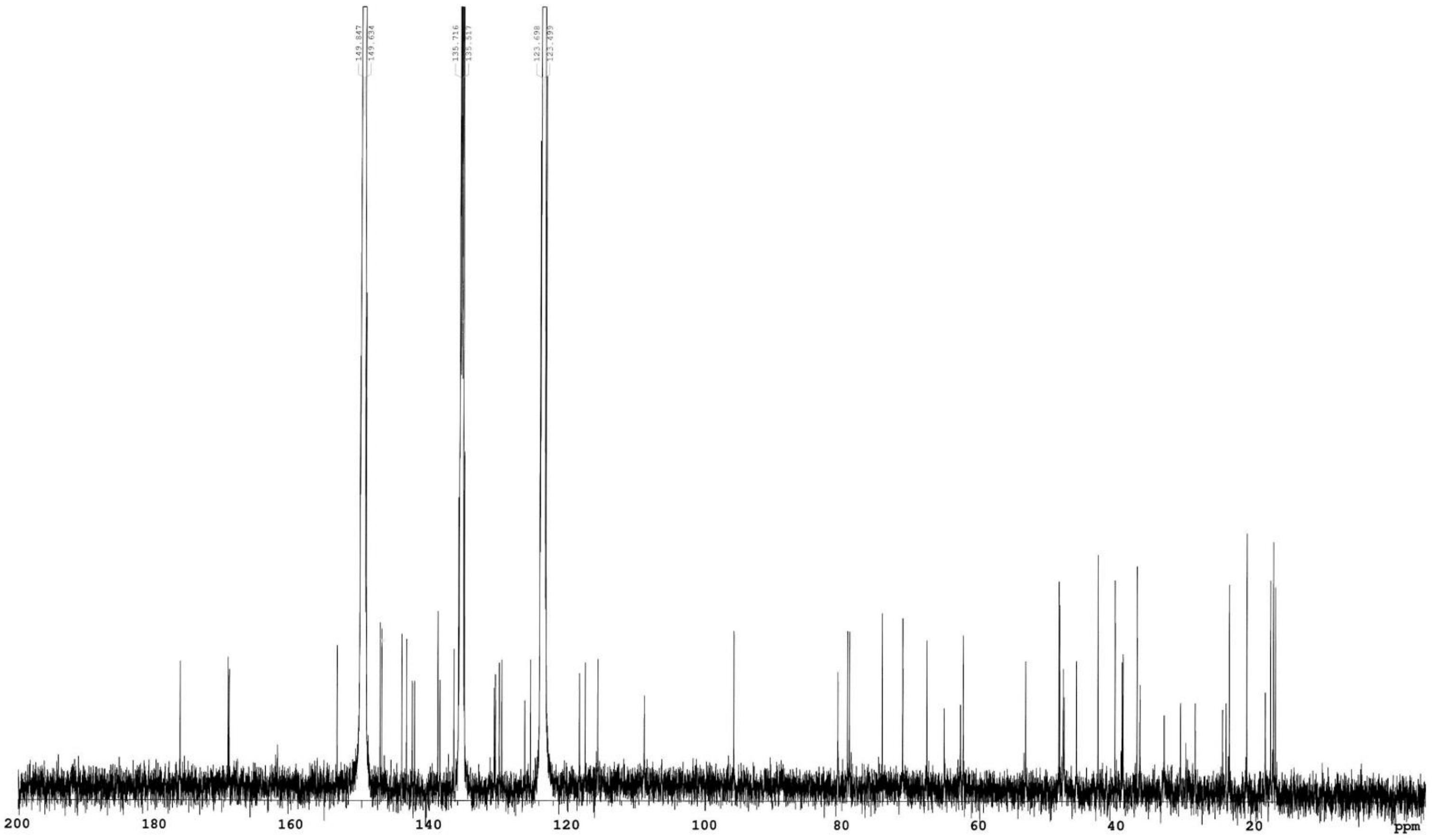

Figure S7. ${}^{13}C$ NMR spectrum of **1u** (pyridine-d_5, 126 MHz)

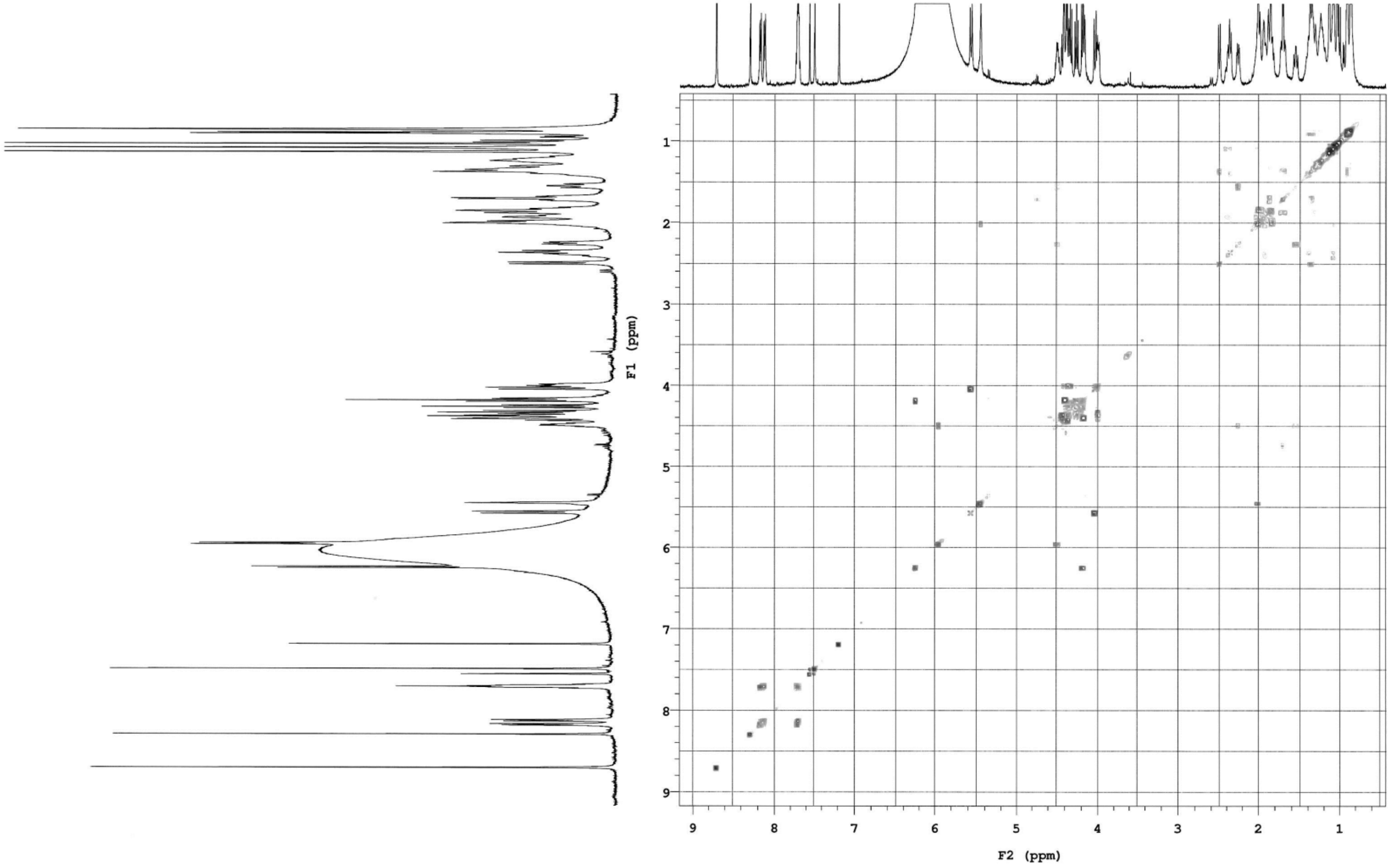

Figure S8. ^{1}H-^{1}H COSY spectrum of **1u** (pyridine-d_5, 500 MHz)

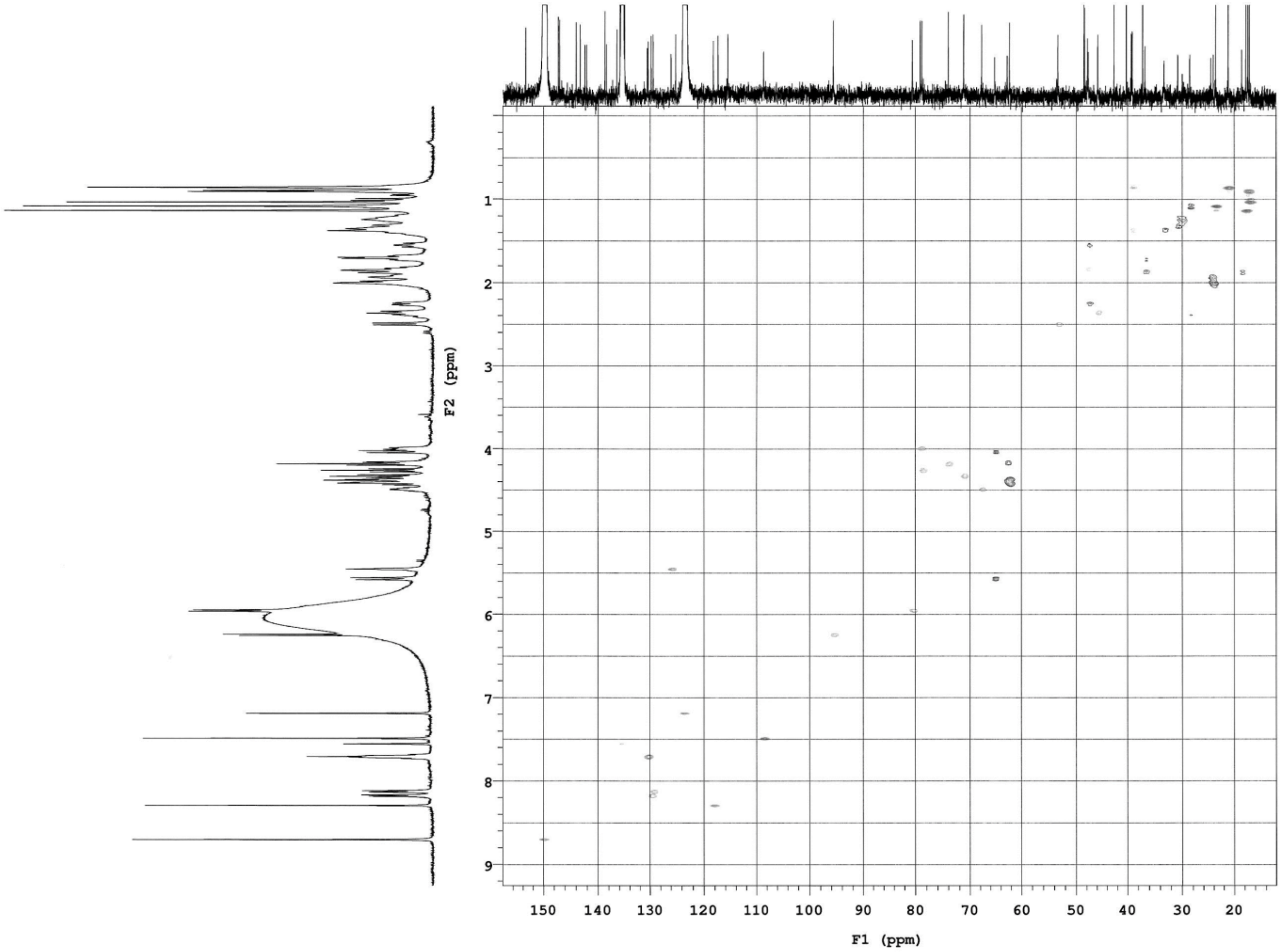

Figure S9. HSQC spectrum of **1u** (pyridine-d_5)

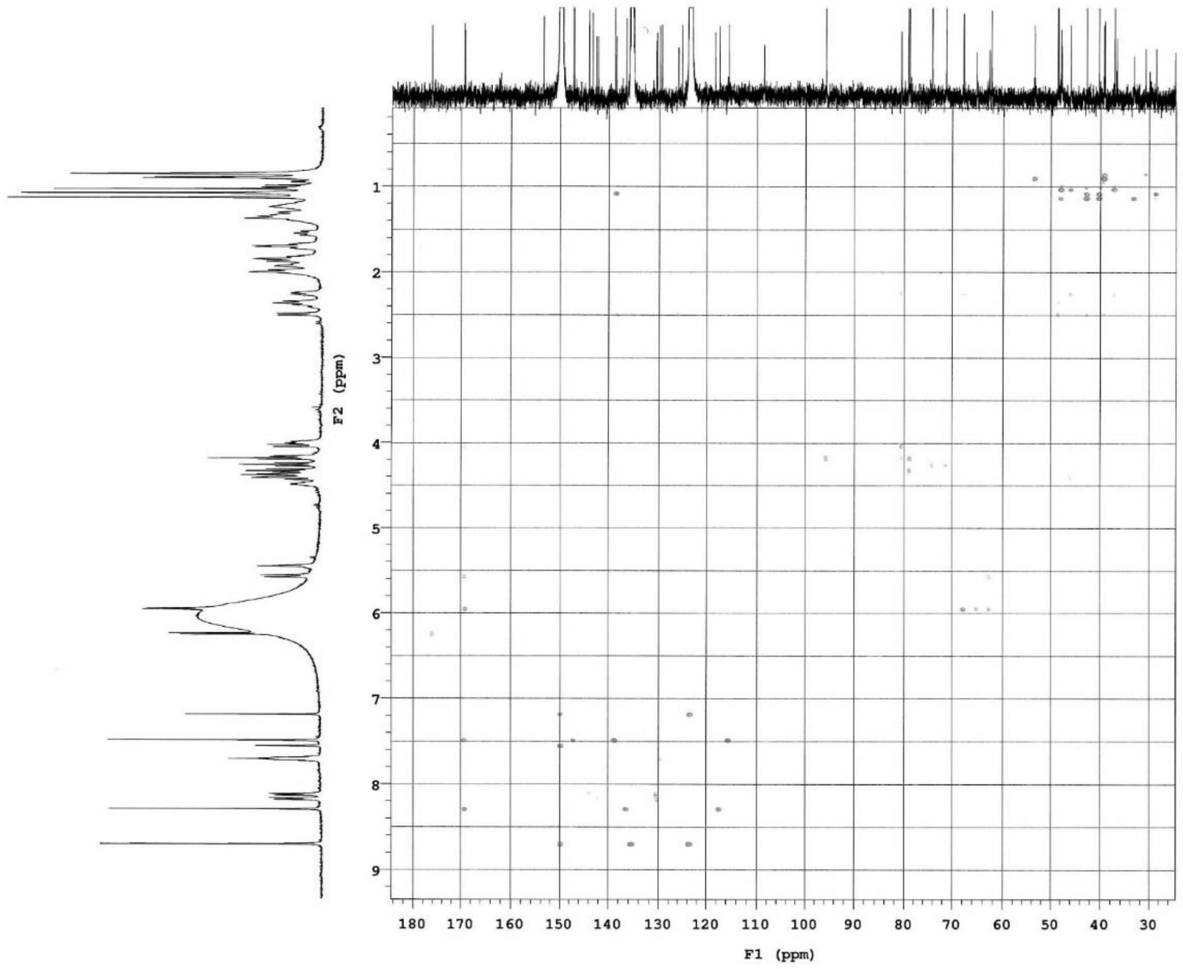

Figure S10. HMBC spectrum of **1u** (pyridine-d_5, 500 MHz)

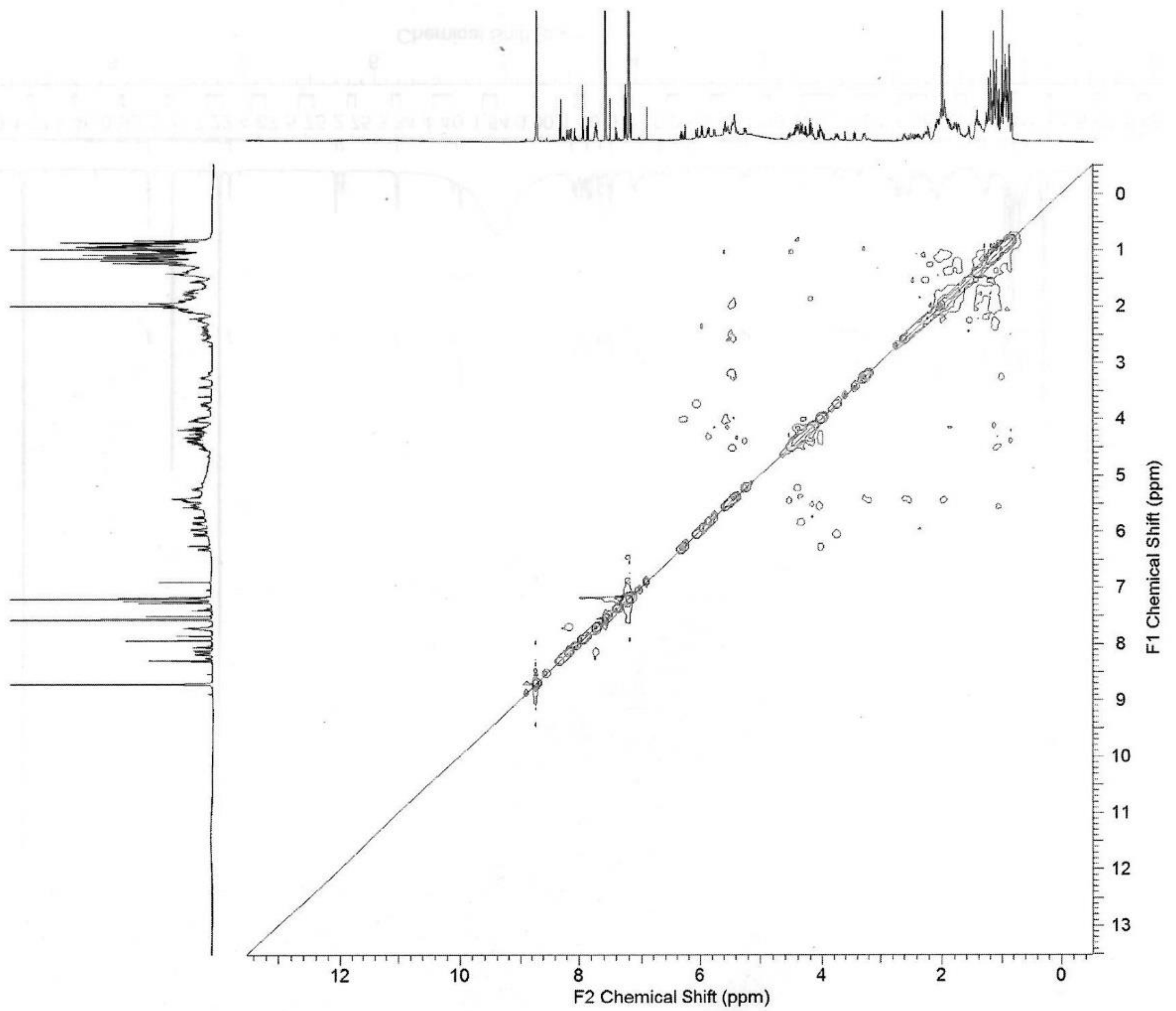

Figure S11. NOESY spectrum of **1o,u** (pyridine-d_5, 500 MHz)

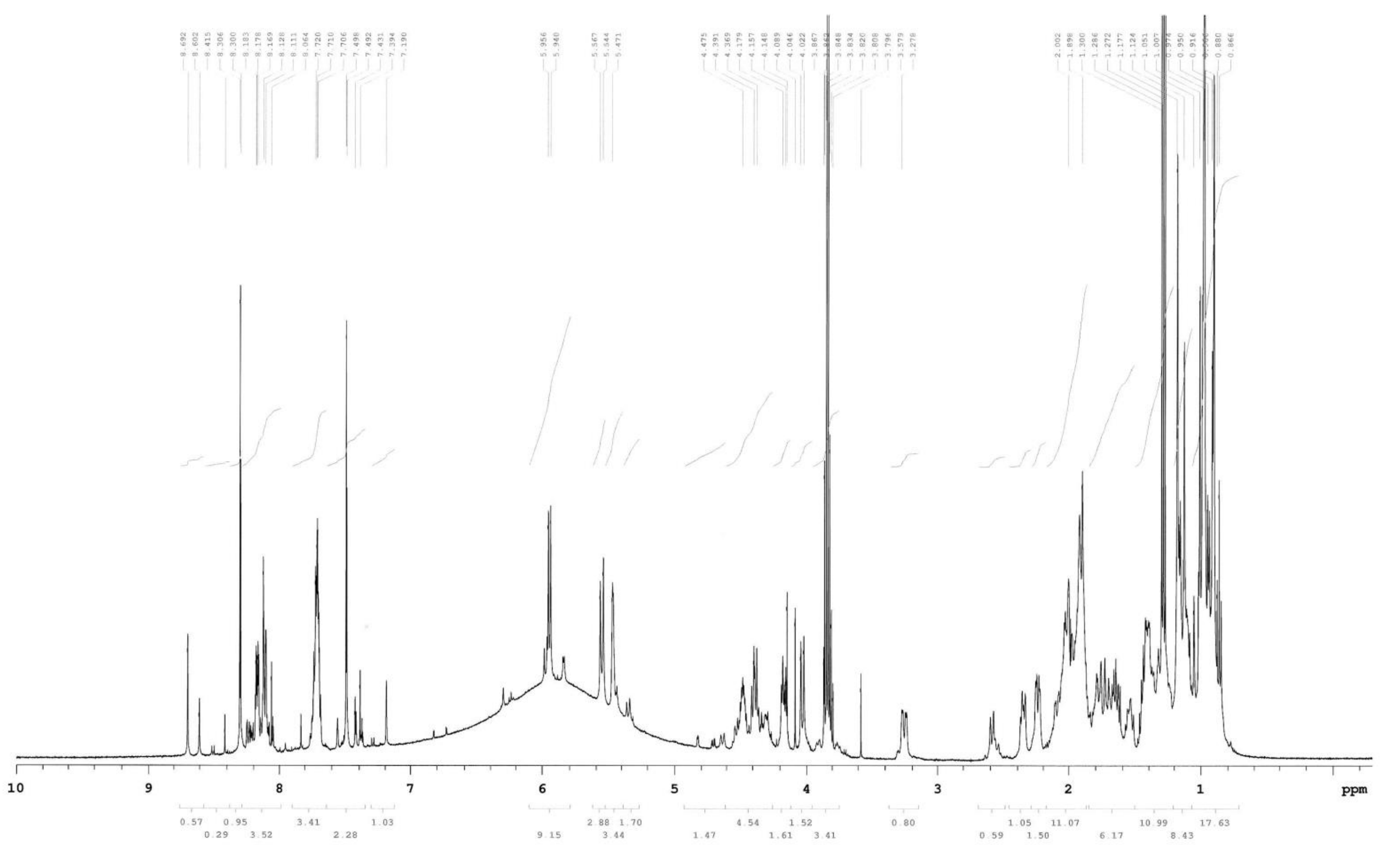

Figure S12. ^{13}H NMR spectrum of **2** (pyridine-d_5, 500 MHz)

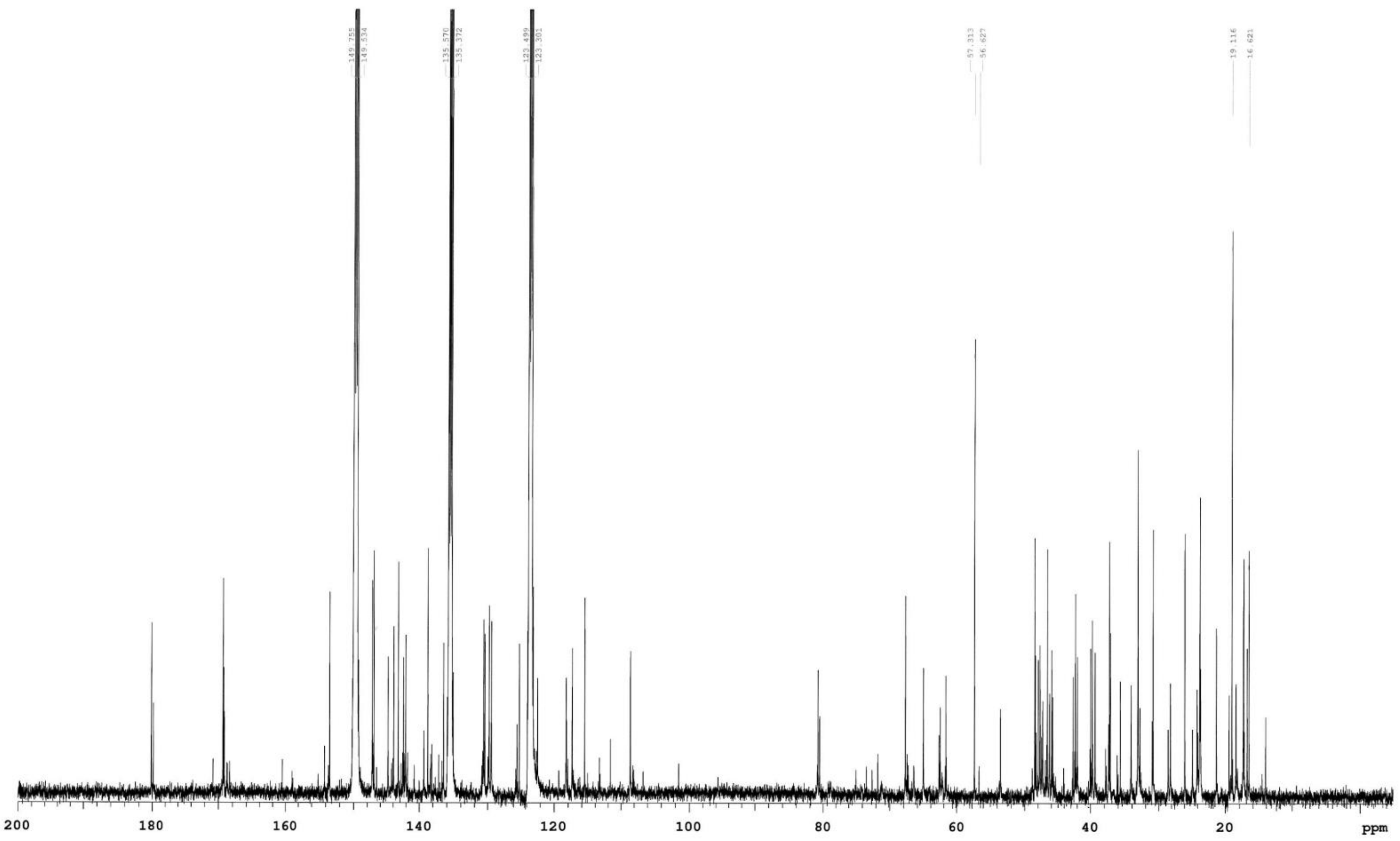

Figure S13. ^{13}C NMR spectrum of **2** (pyridine-d_5, 126 MHz)

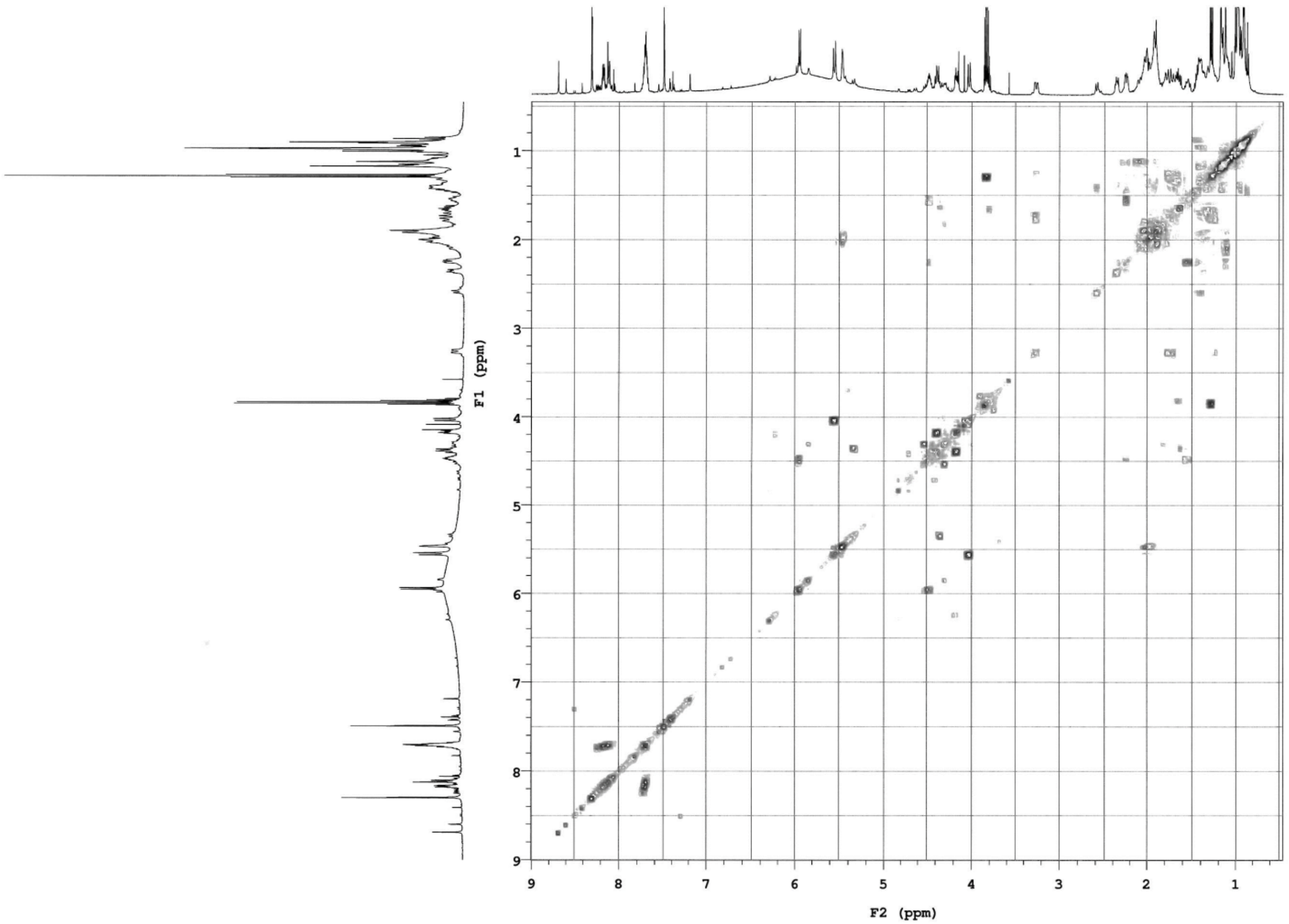

Figure S14. ^{1}H-^{1}H COSY spectrum of **2** (pyridine-d_5, 500 MHz)

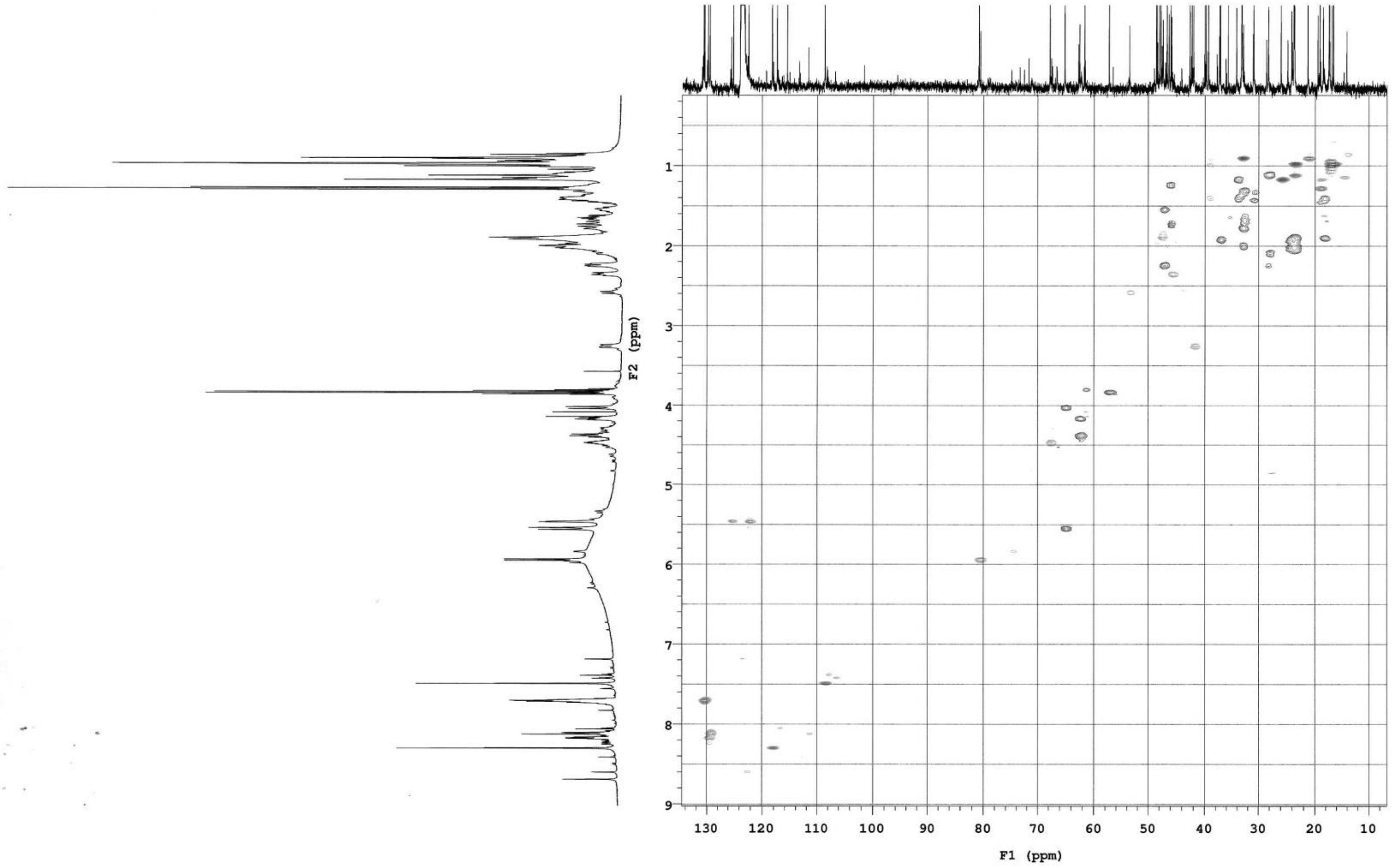

Figure S15. HSQC spectrum of **2** (pyridine-d_5)

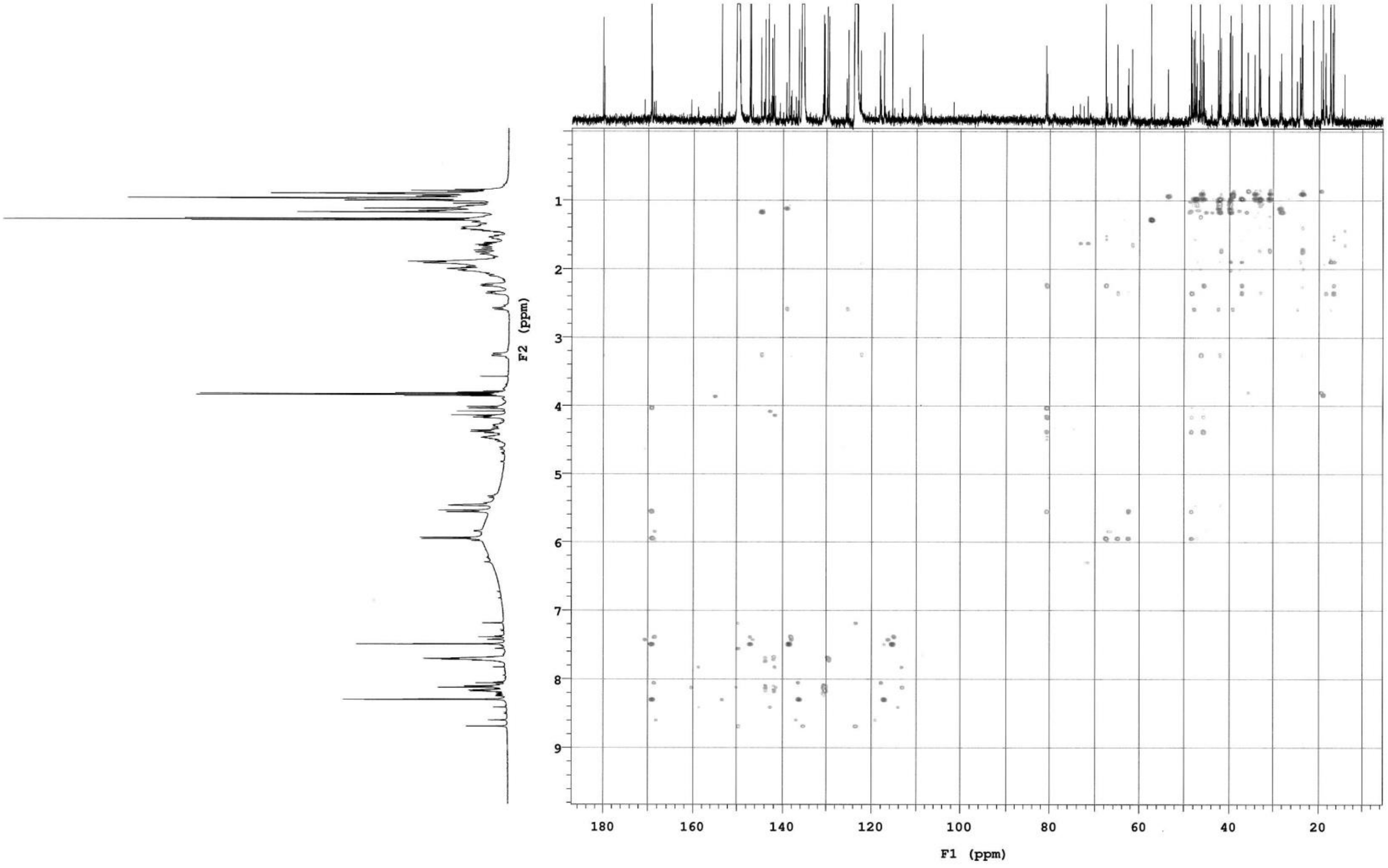

Figure S16. HMBC spectrum of **2** (pyridine-d_5)

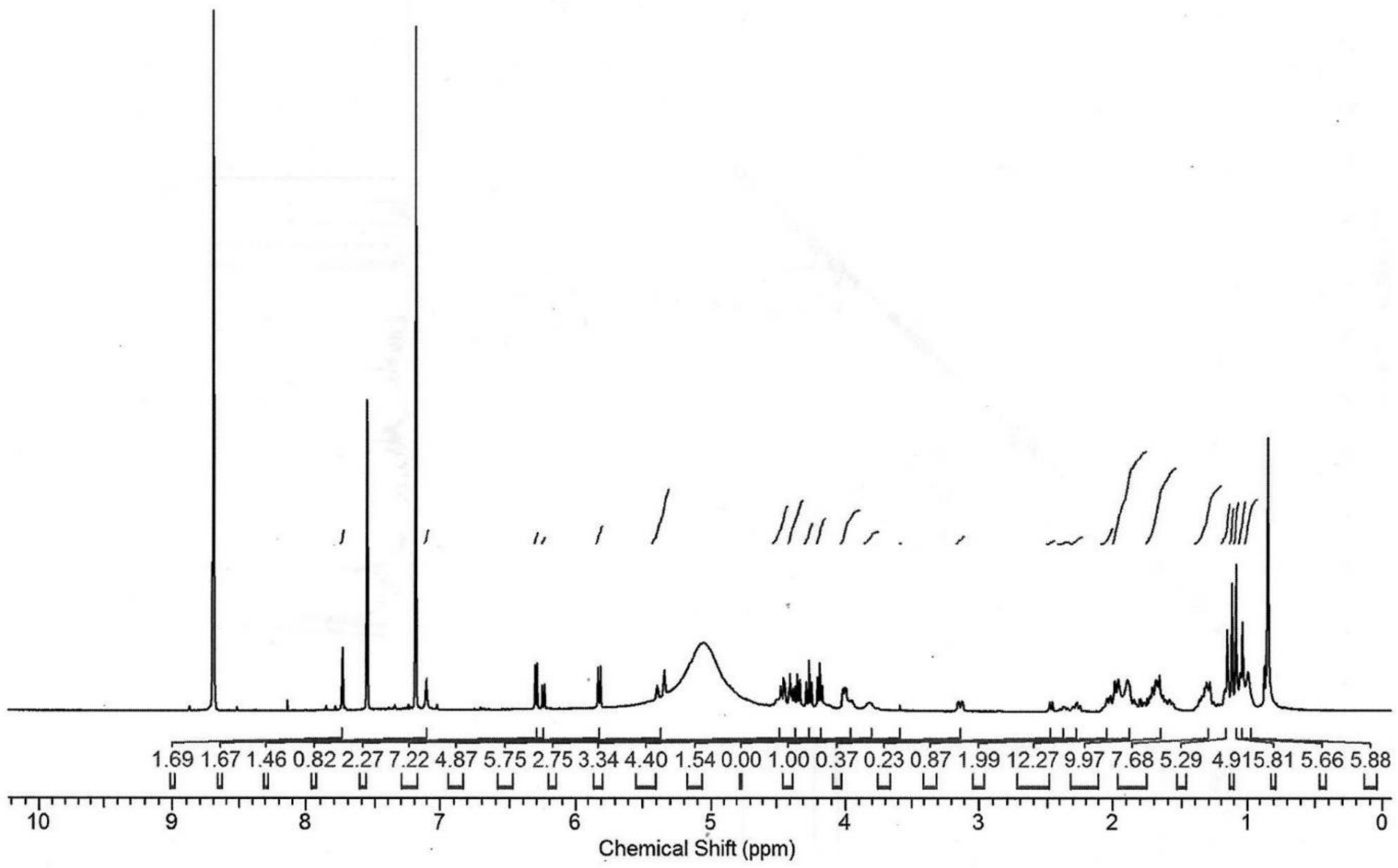

Figure S17. ^{1}H NMR spectrum of **4** (pyridine-d_5, 500 MHz)

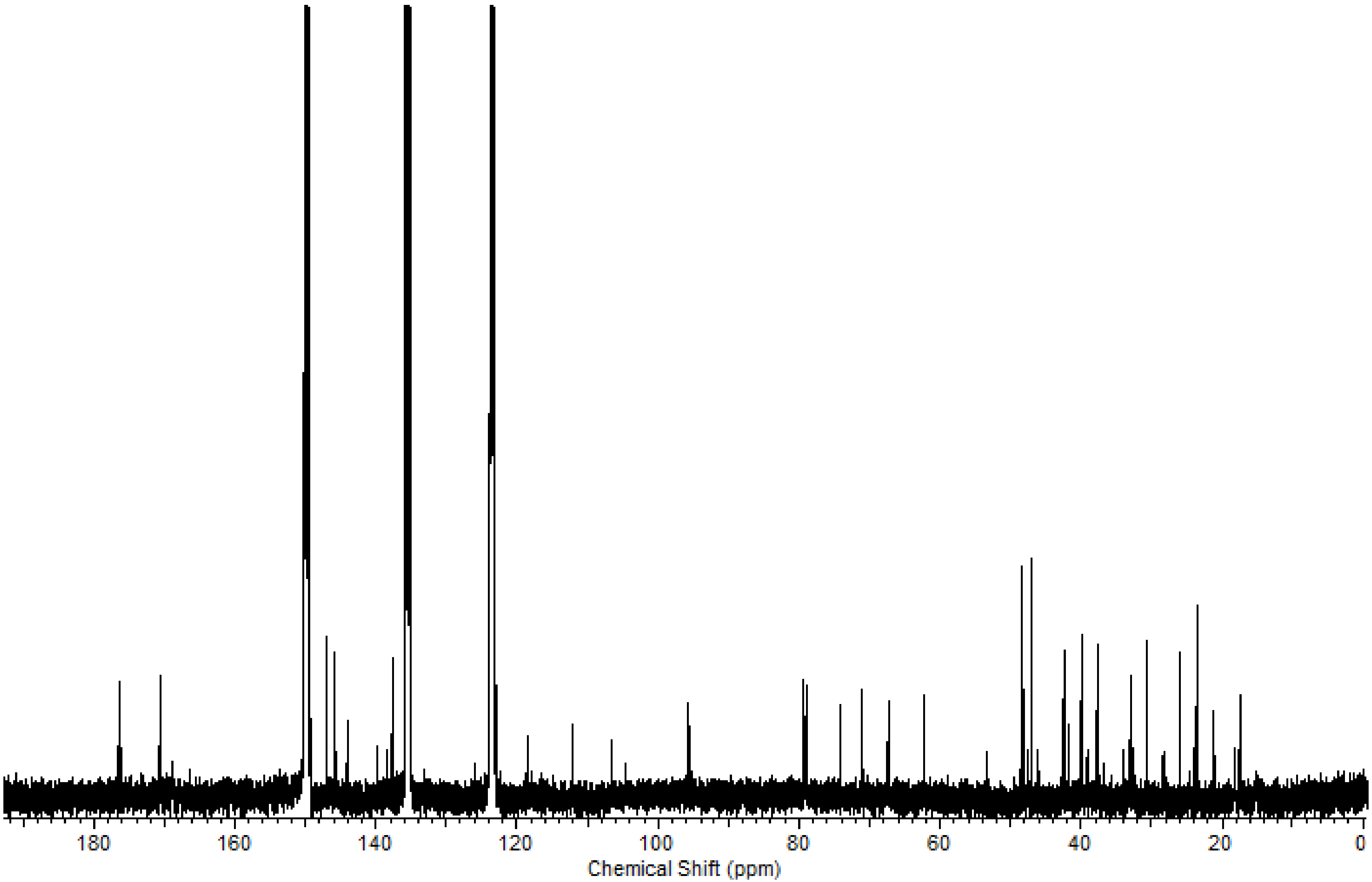

Figure S18. ^{13}C NMR spectrum of **4** (pyridine-d_5, 126 MHz)

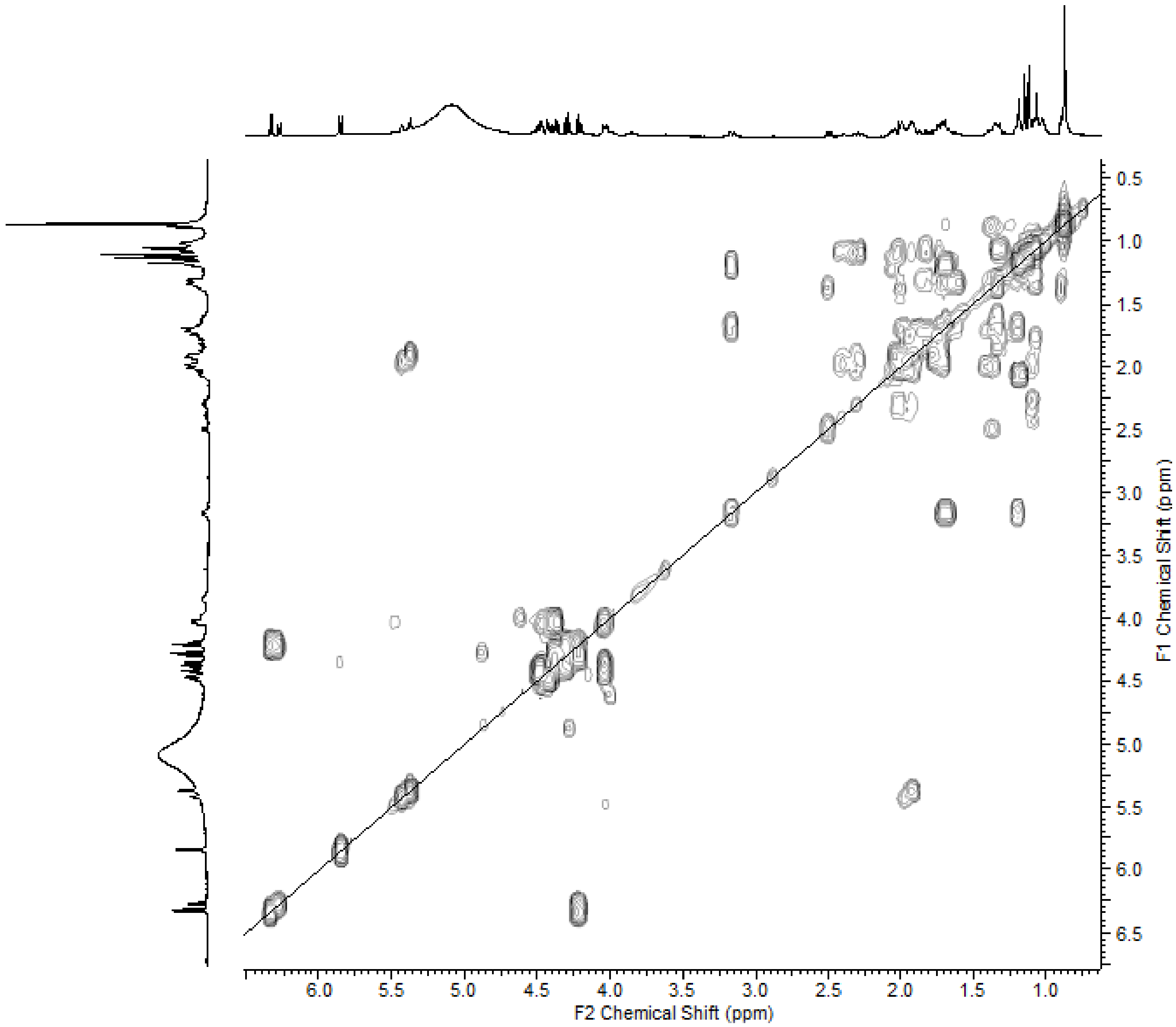

Figure S19. ^{1}H-^{1}H COSY spectrum of **4** (pyridine-d_5, 500 MHz)

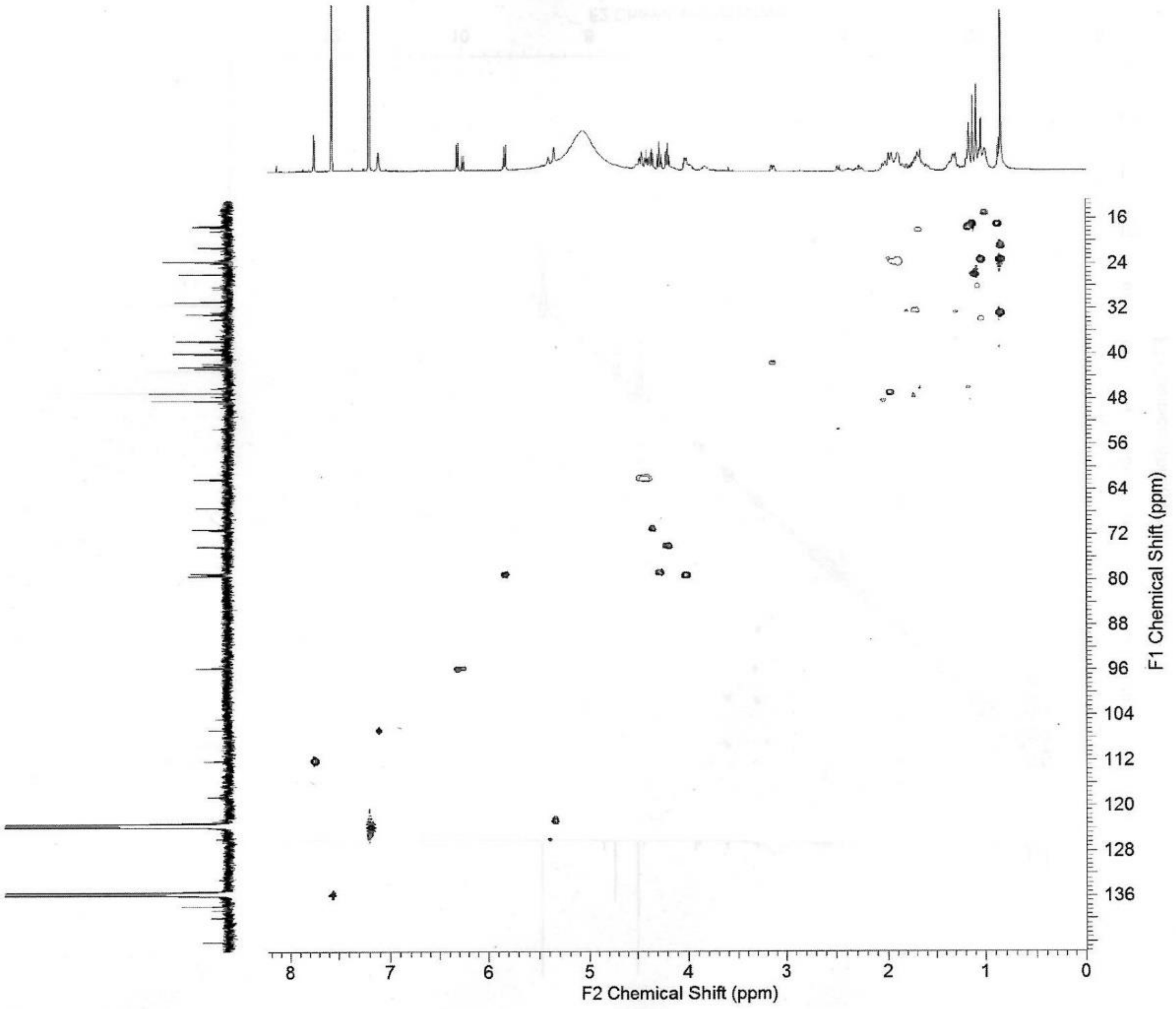

Figure S20. HSQC spectrum of **4** (pyridine-d_5)

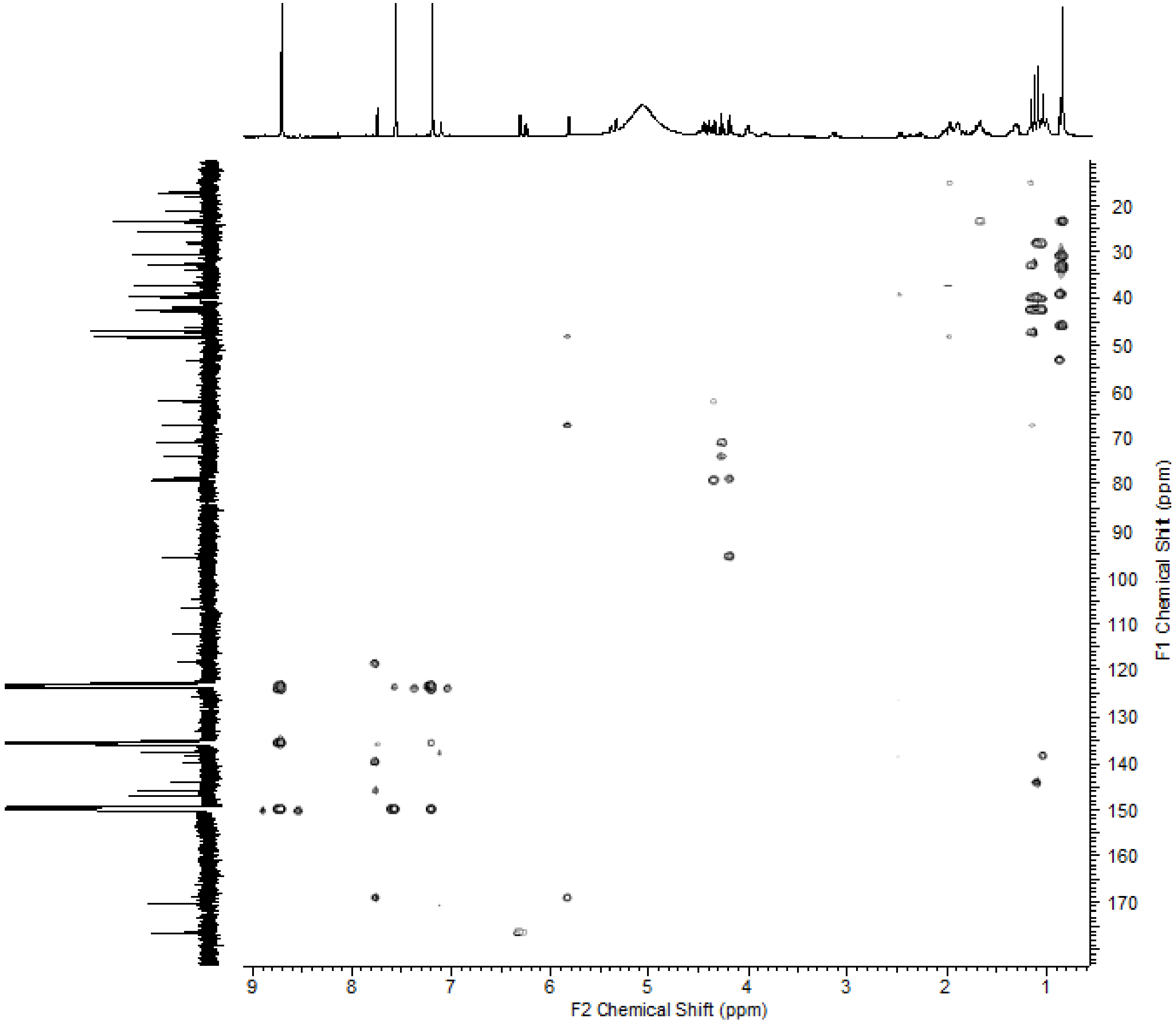

Figure S21. HMBC spectrum of **4** (pyridine-*d*5)

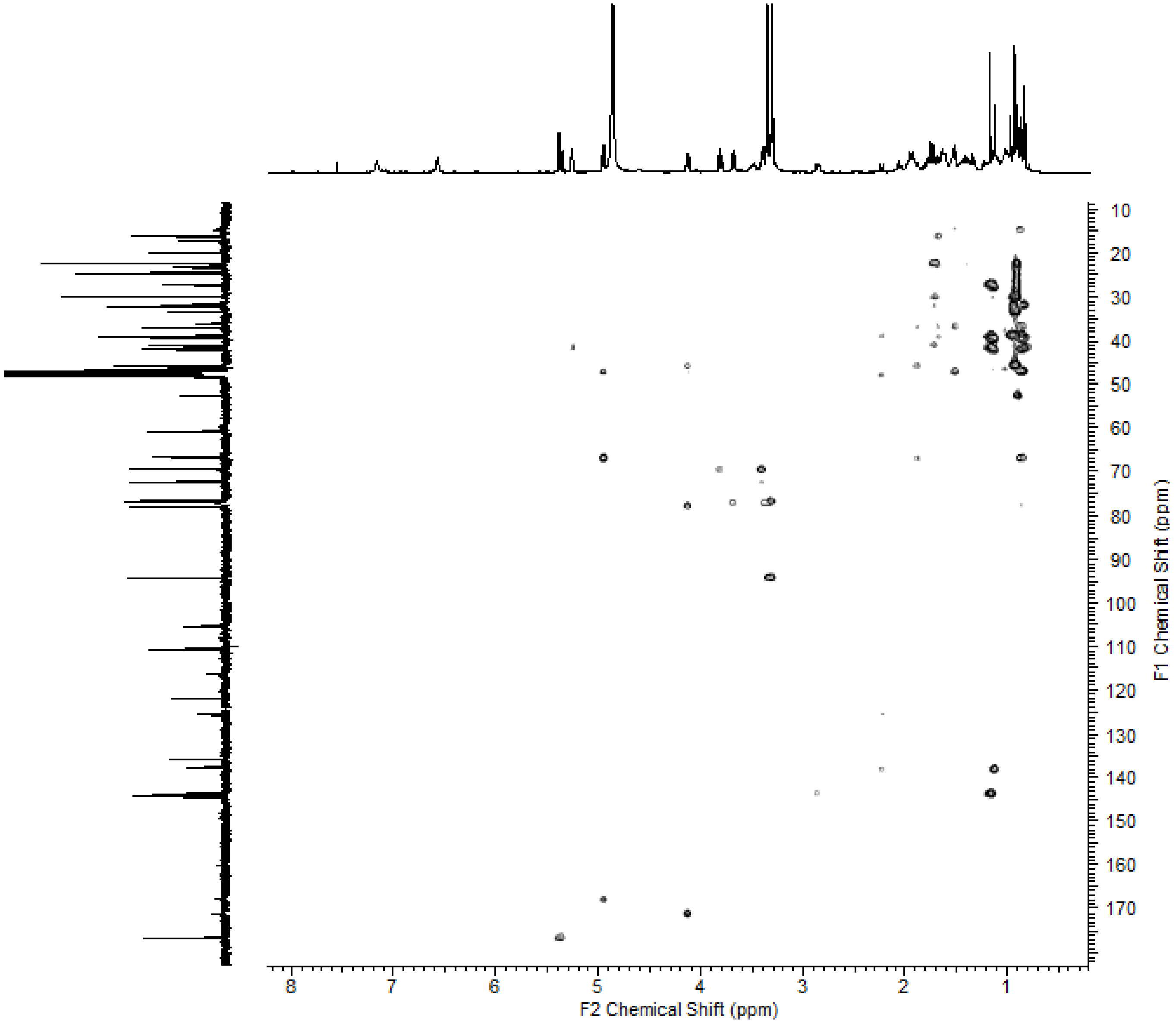

Figure S22. HMBC spectrum of **4** (CD_3OD, 500 MHz)

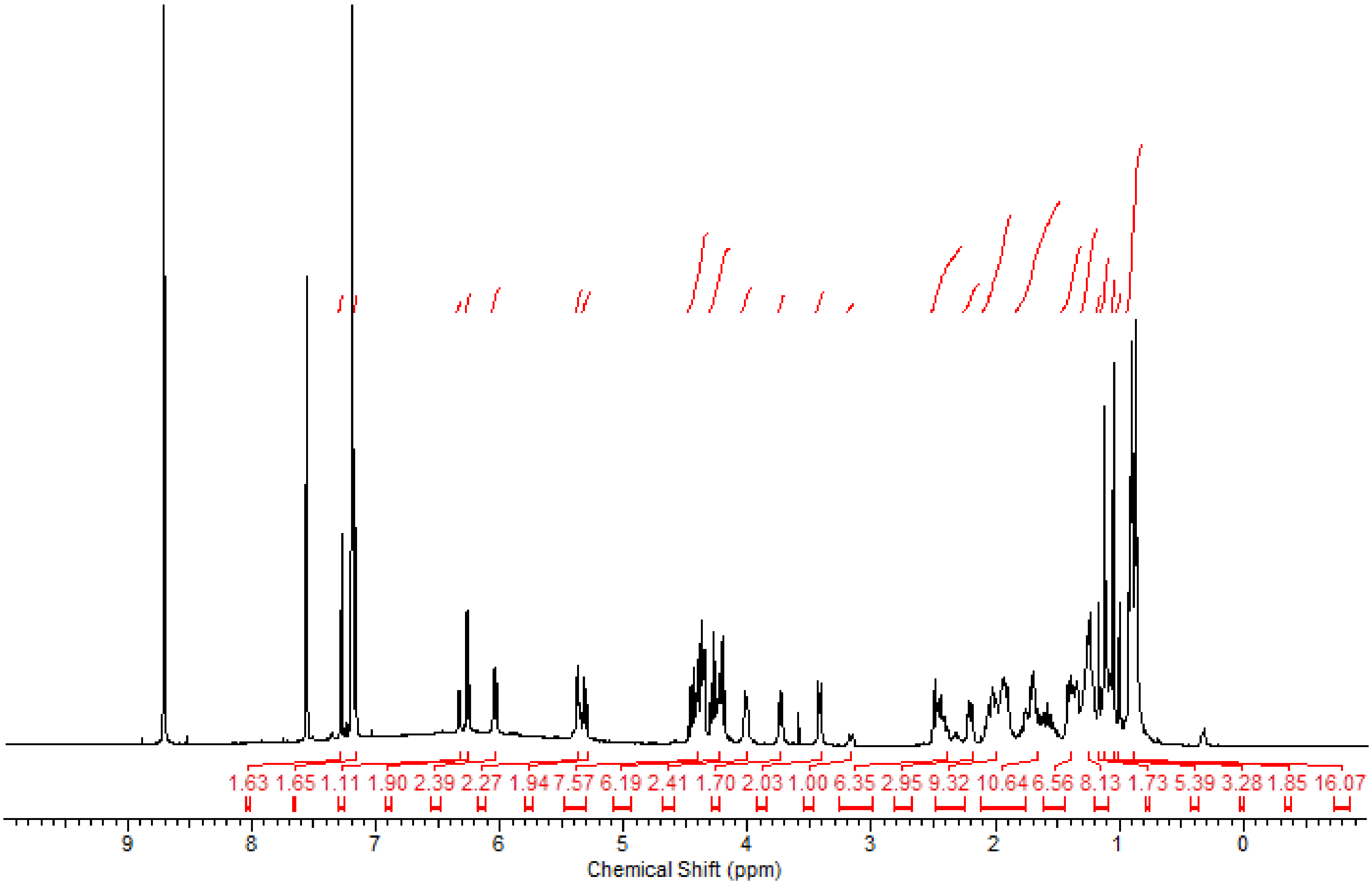

Figure S23. ¹H NMR spectrum of **9** (pyridine-d_5, 500 MHz)

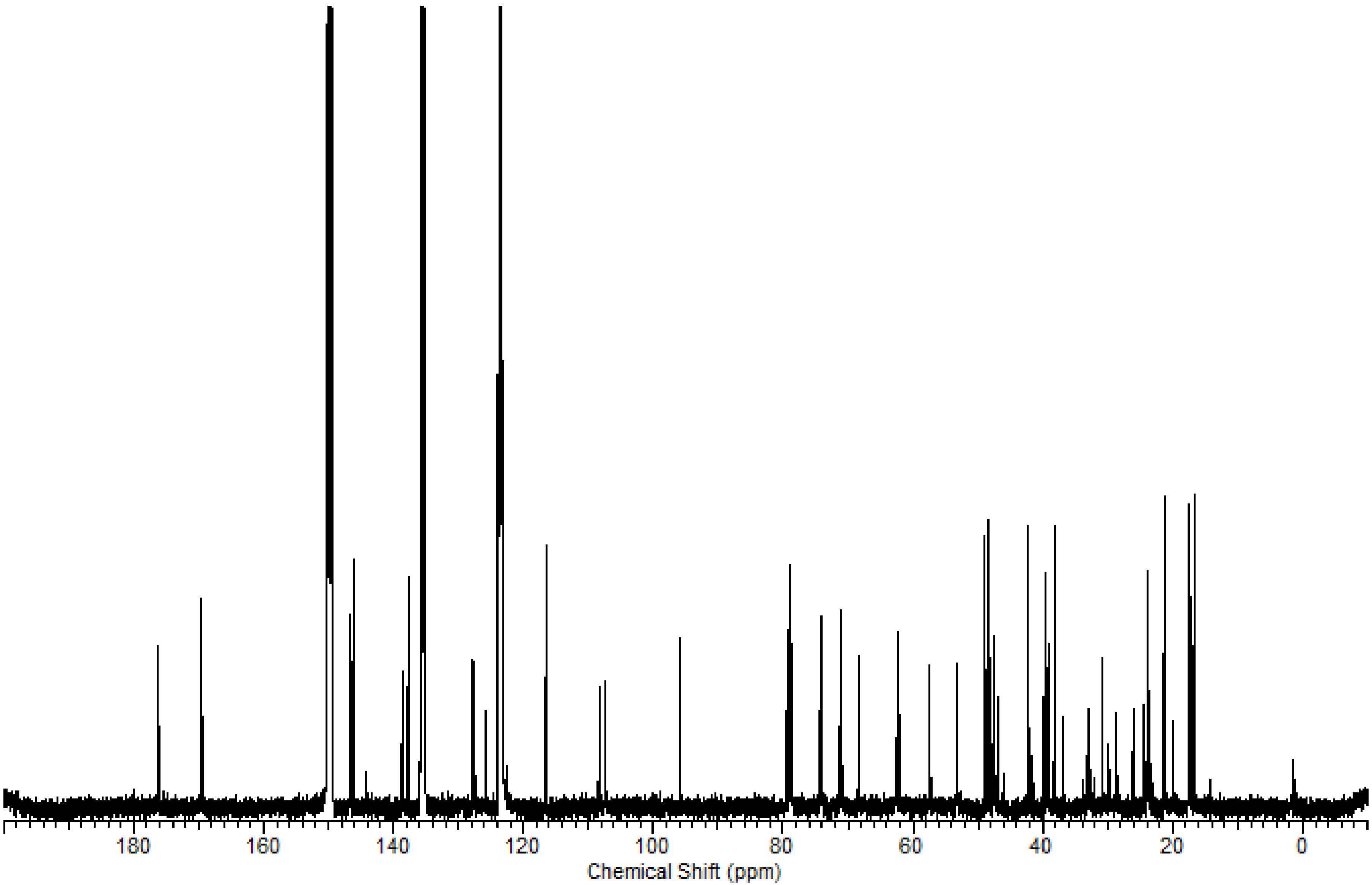

Figure S24. ^{13}C NMR spectrum of **9** (pyridine-d_5, 126 MHz)

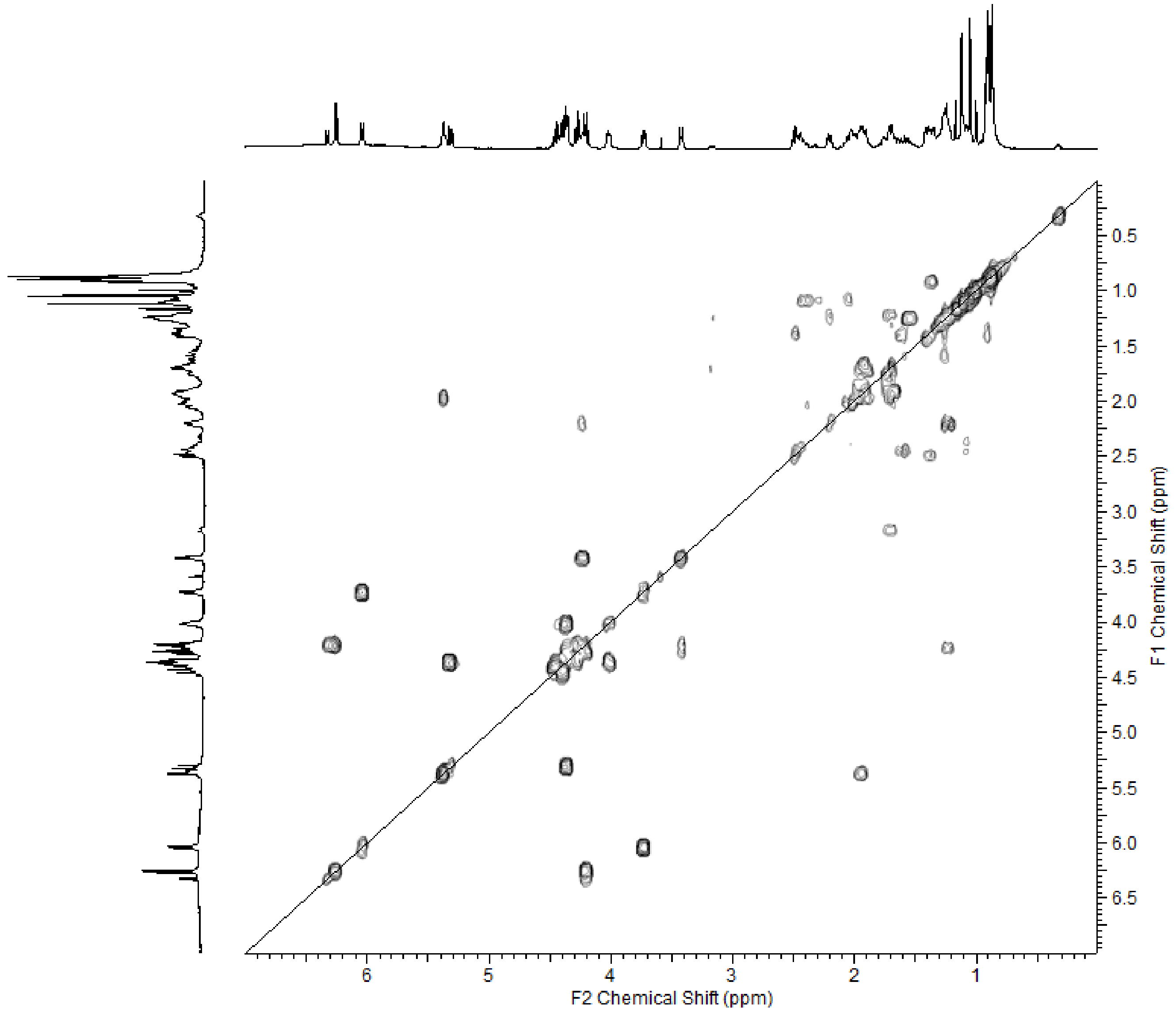

Figure S25. ^{1}H-^{1}H COSY spectrum of **9** (pyridine-d_5, 500 MHz)

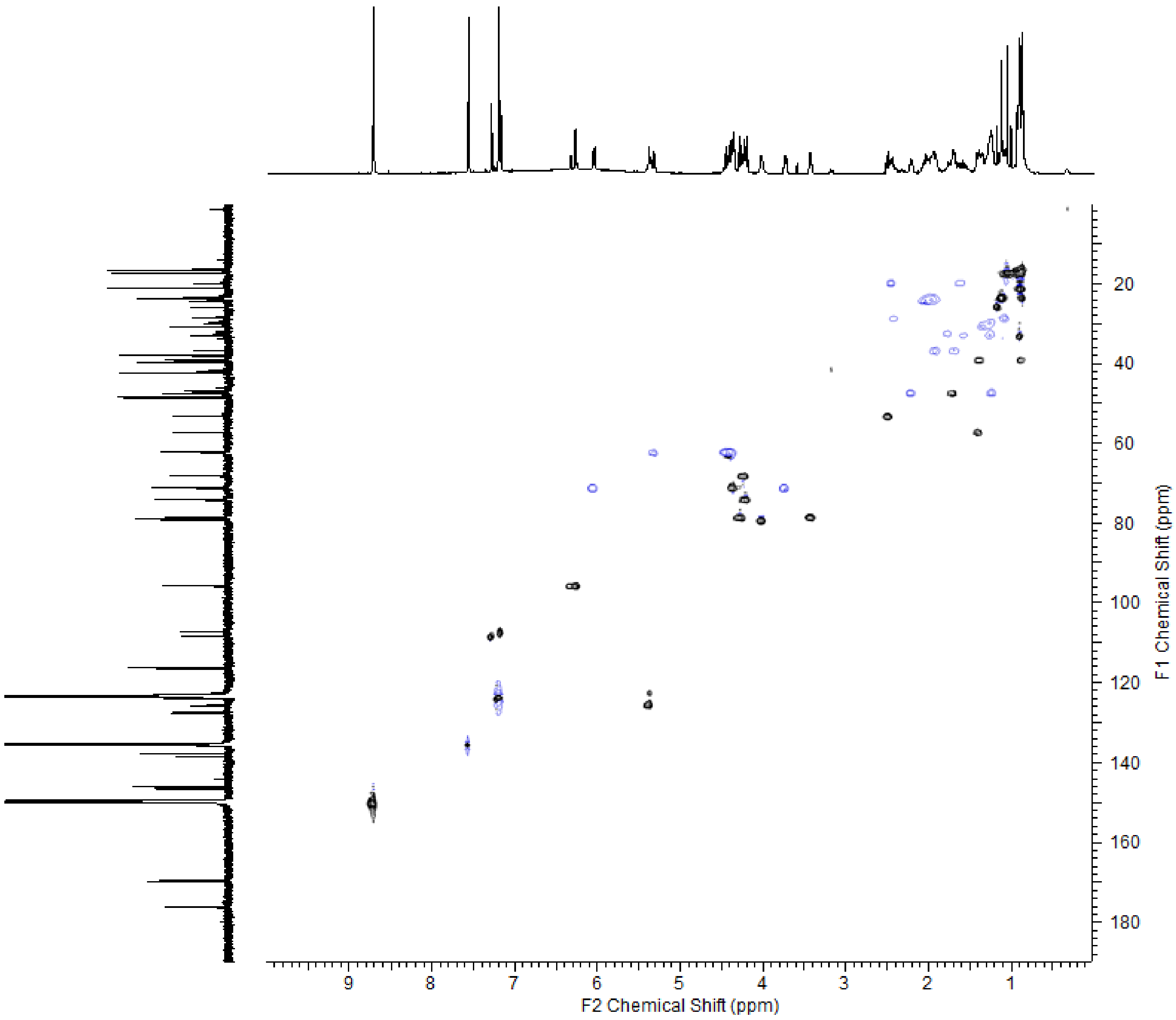

Figure S26. HSQC spectrum of **9** (pyridine-d_5)

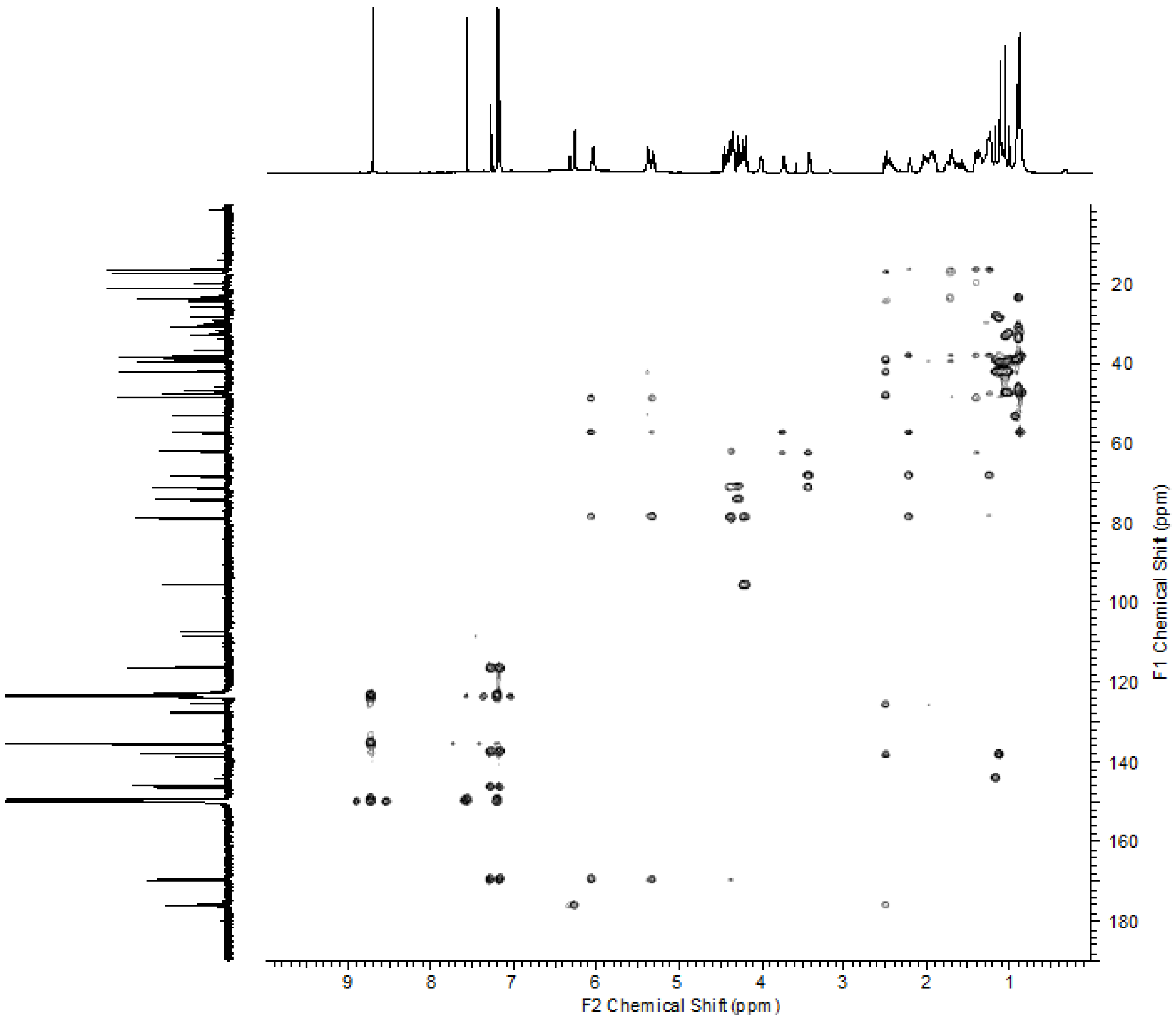

Figure S27. HMBC spectrum of **9** (pyridine-d_5)

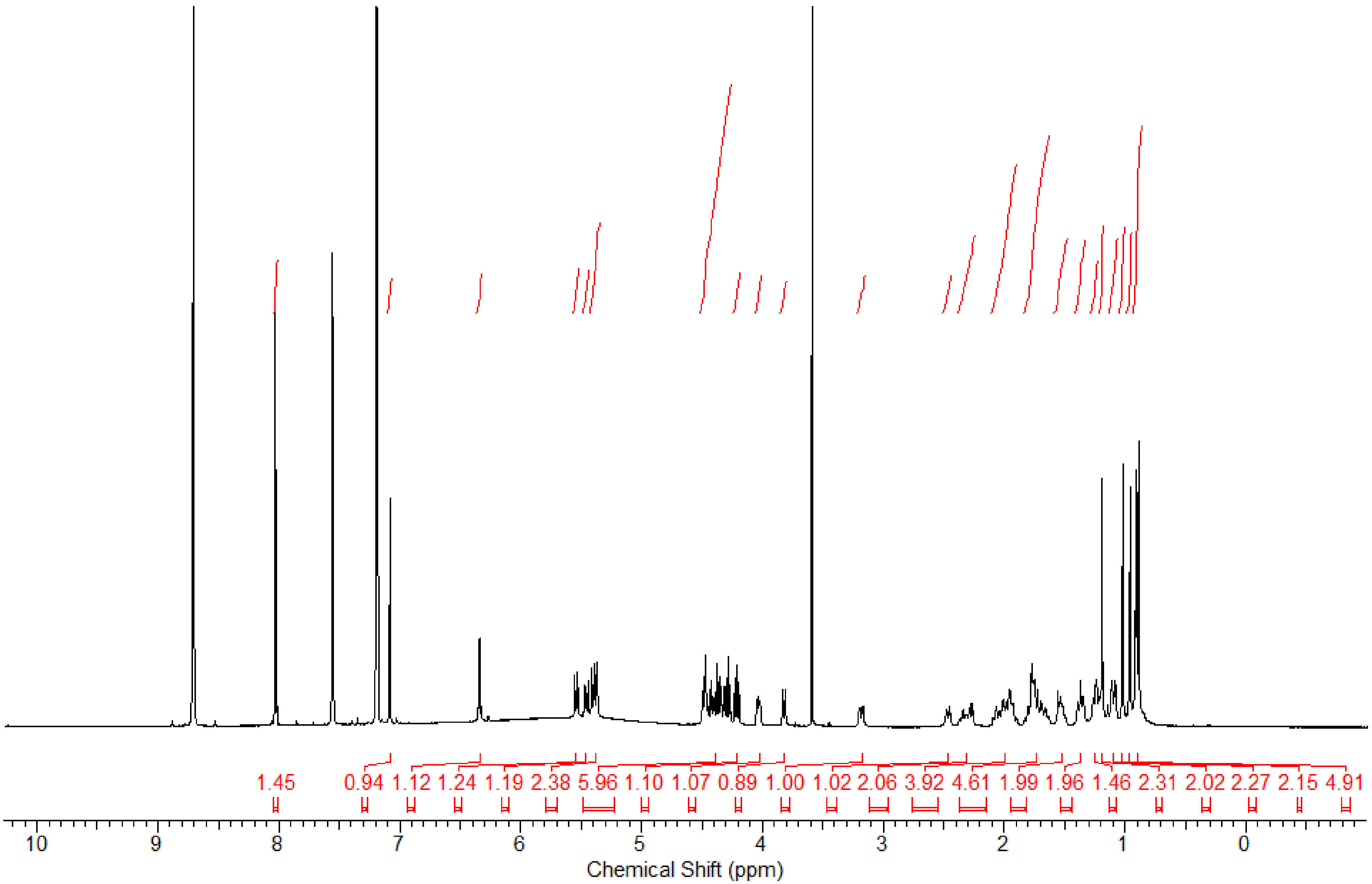

Figure S28. ^{1}H NMR spectrum of **10** (pyridine-d_5, 500 MHz)

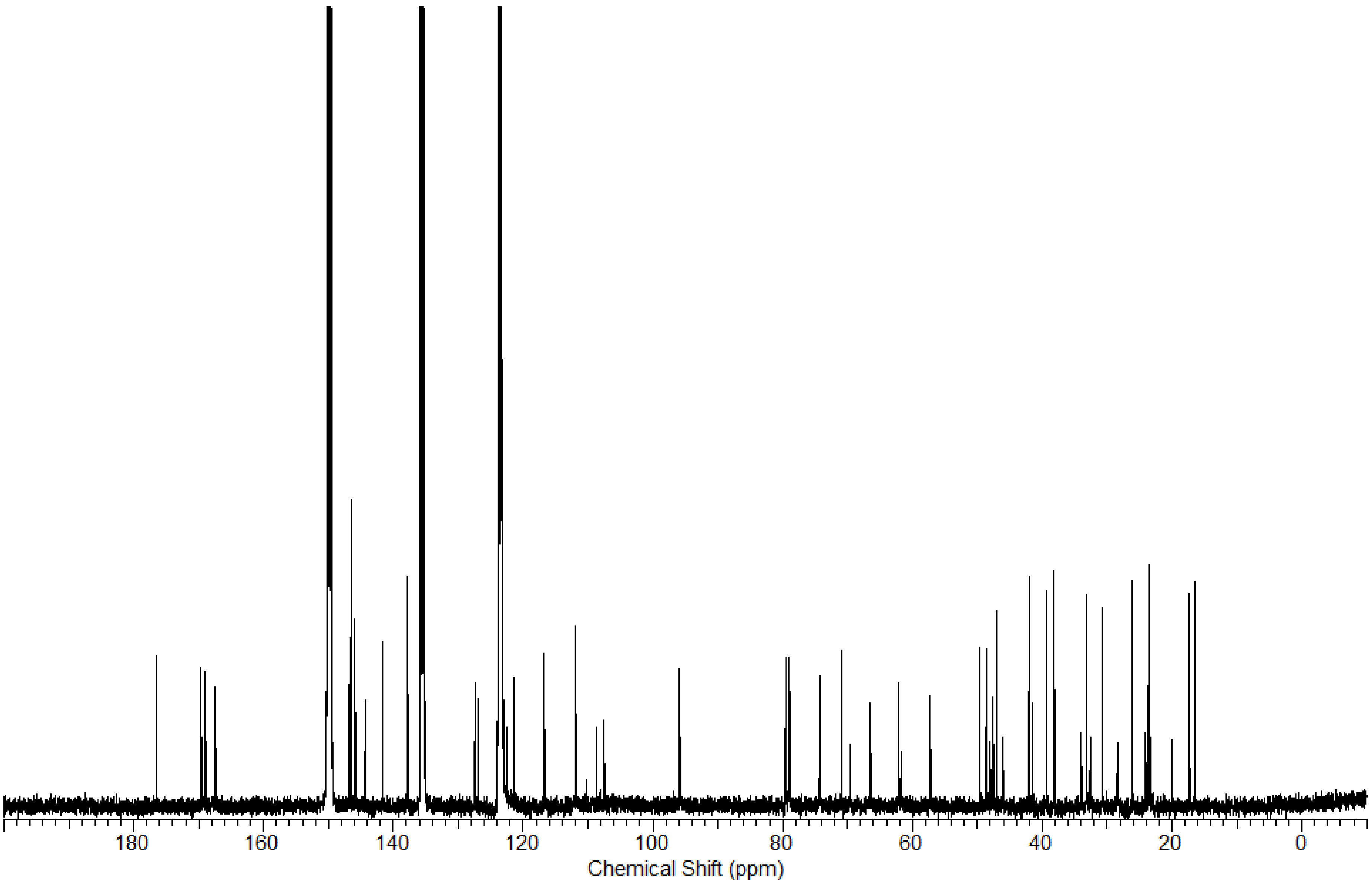

Figure S29. ^{13}C NMR spectrum of **10** (pyridine-*d*$_5$, 126 MHz)

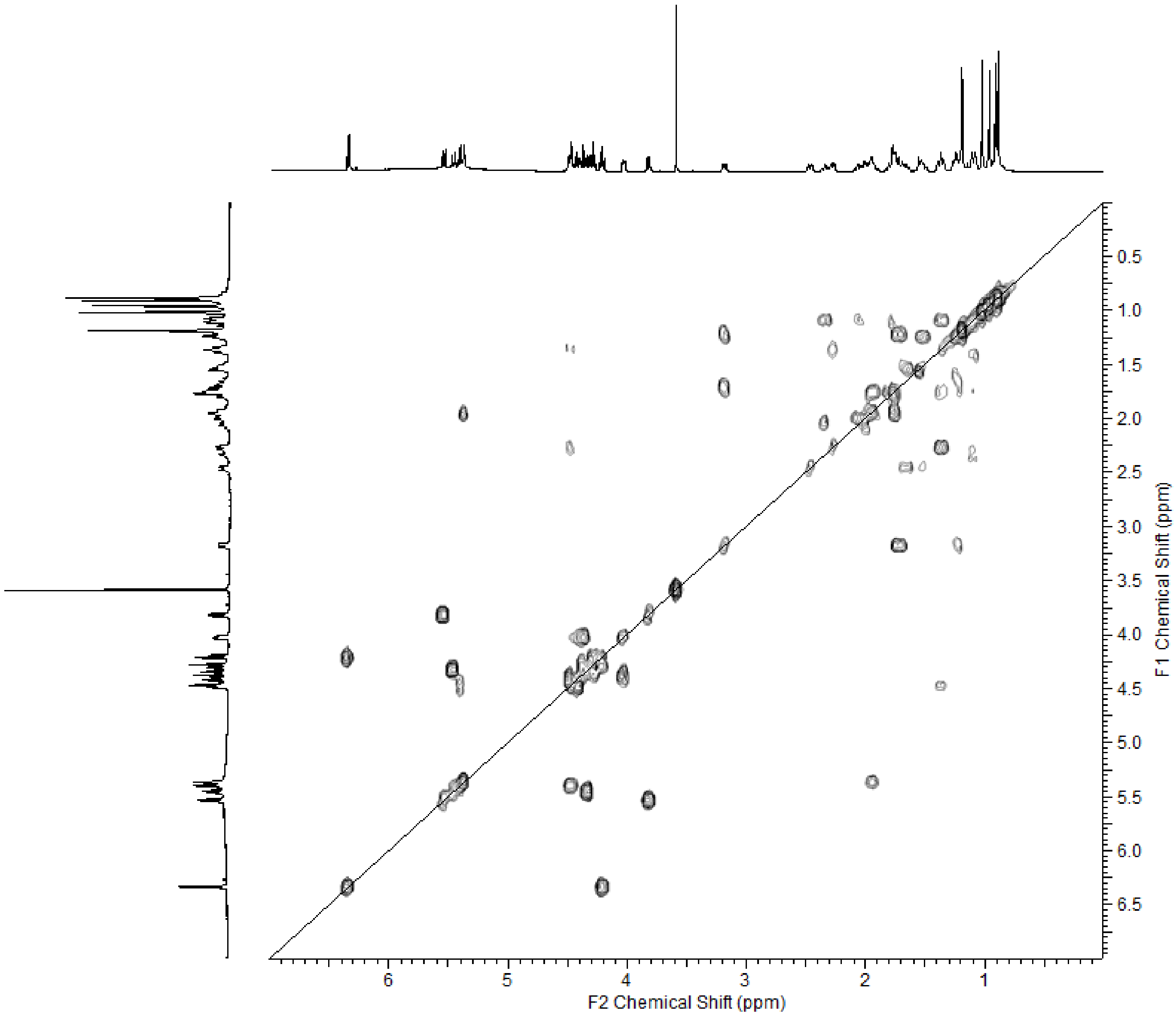

Figure S30. ^{1}H-^{1}H COSY spectrum of **10** (pyridine-d_5, 500 MHz)

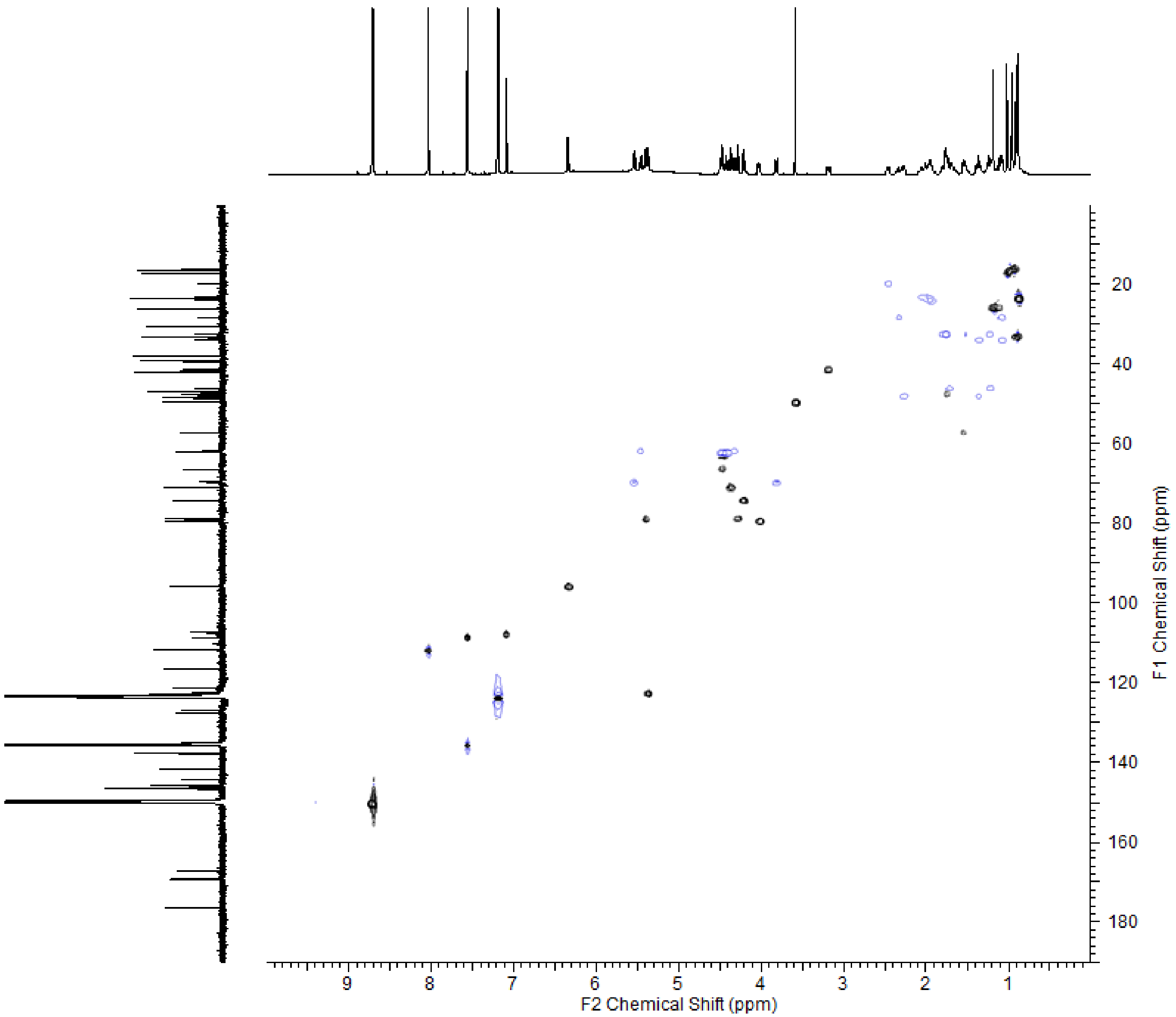

Figure S31. HSQC spectrum of **10** (pyridine-d_5)

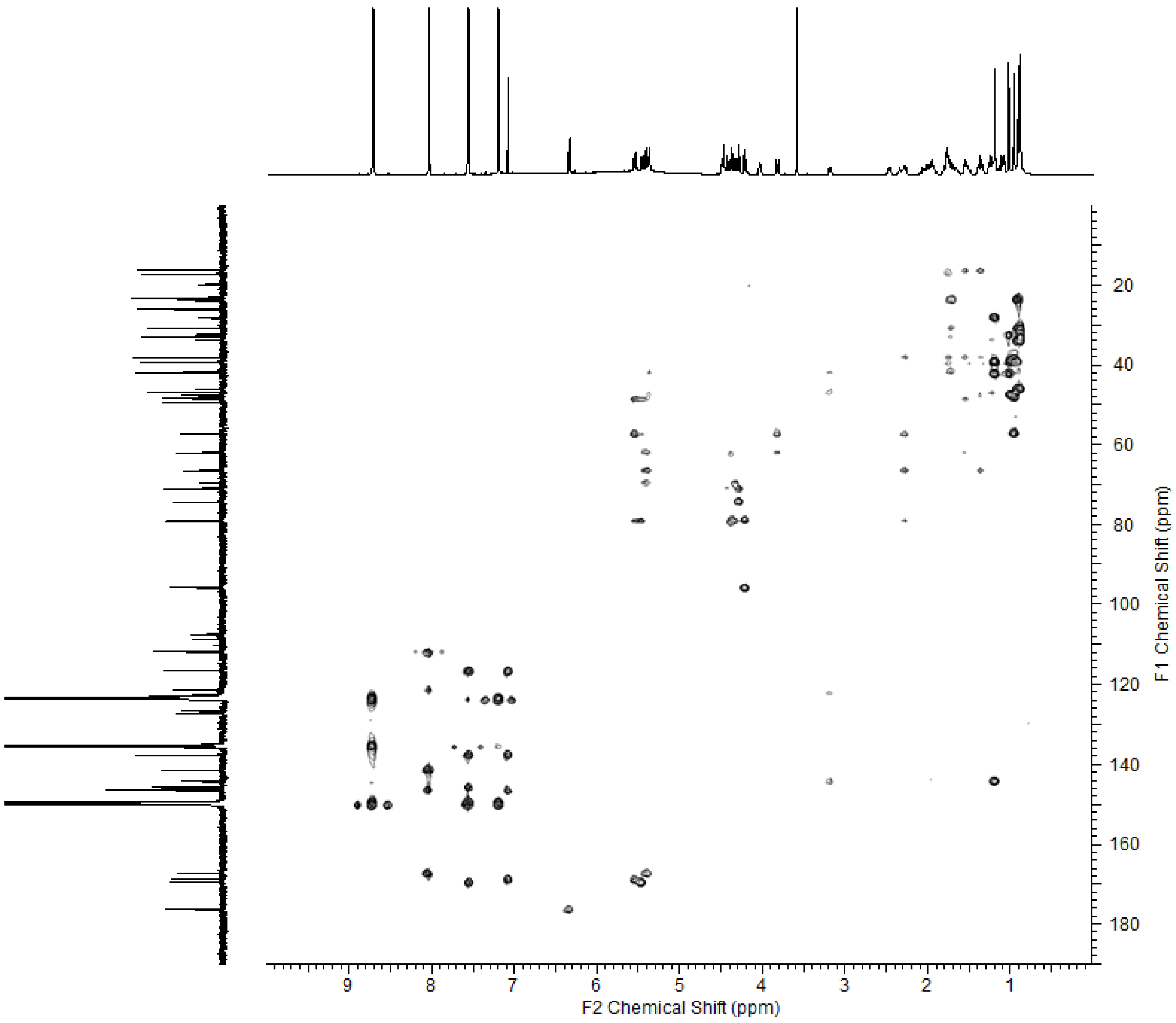

Figure S32. HMBC spectrum of **10** (pyridine-d_5)

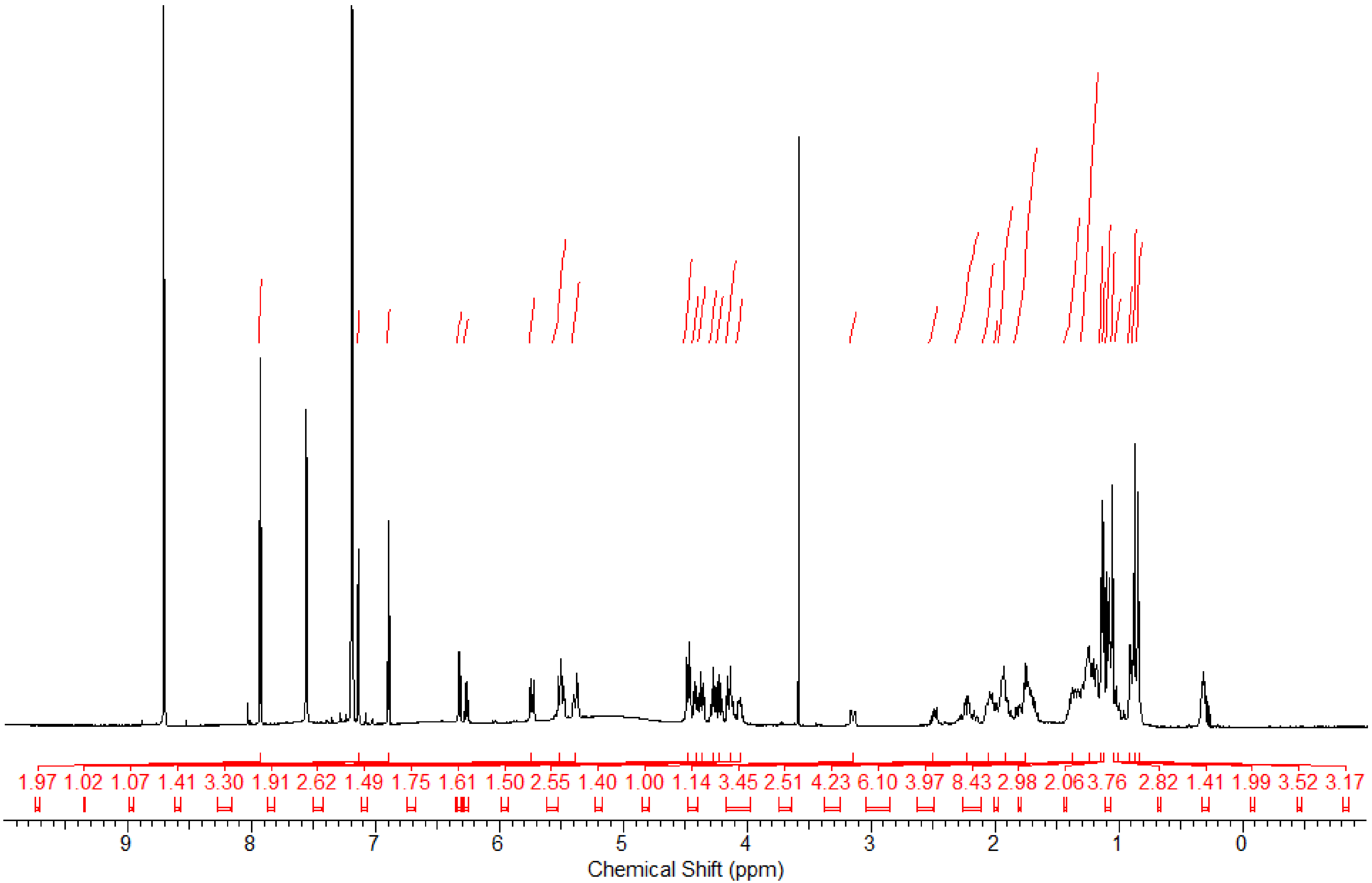

Figure S33. ^{1}H NMR spectrum of **11** (pyridine-d_5, 500 MHz)

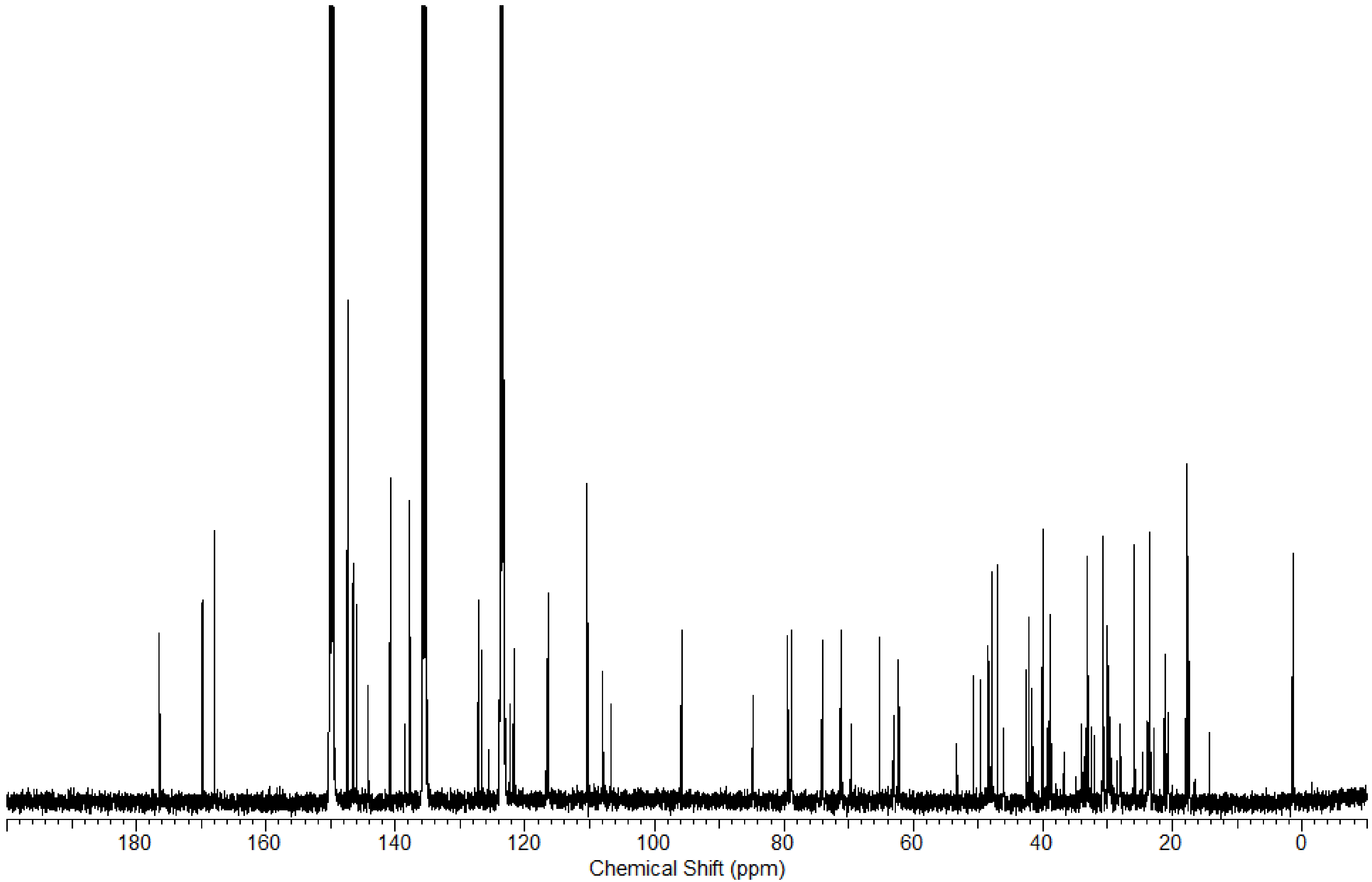

Figure S34. ^{13}C NMR spectrum of **11** (pyridine-d_5, 126 MHz)

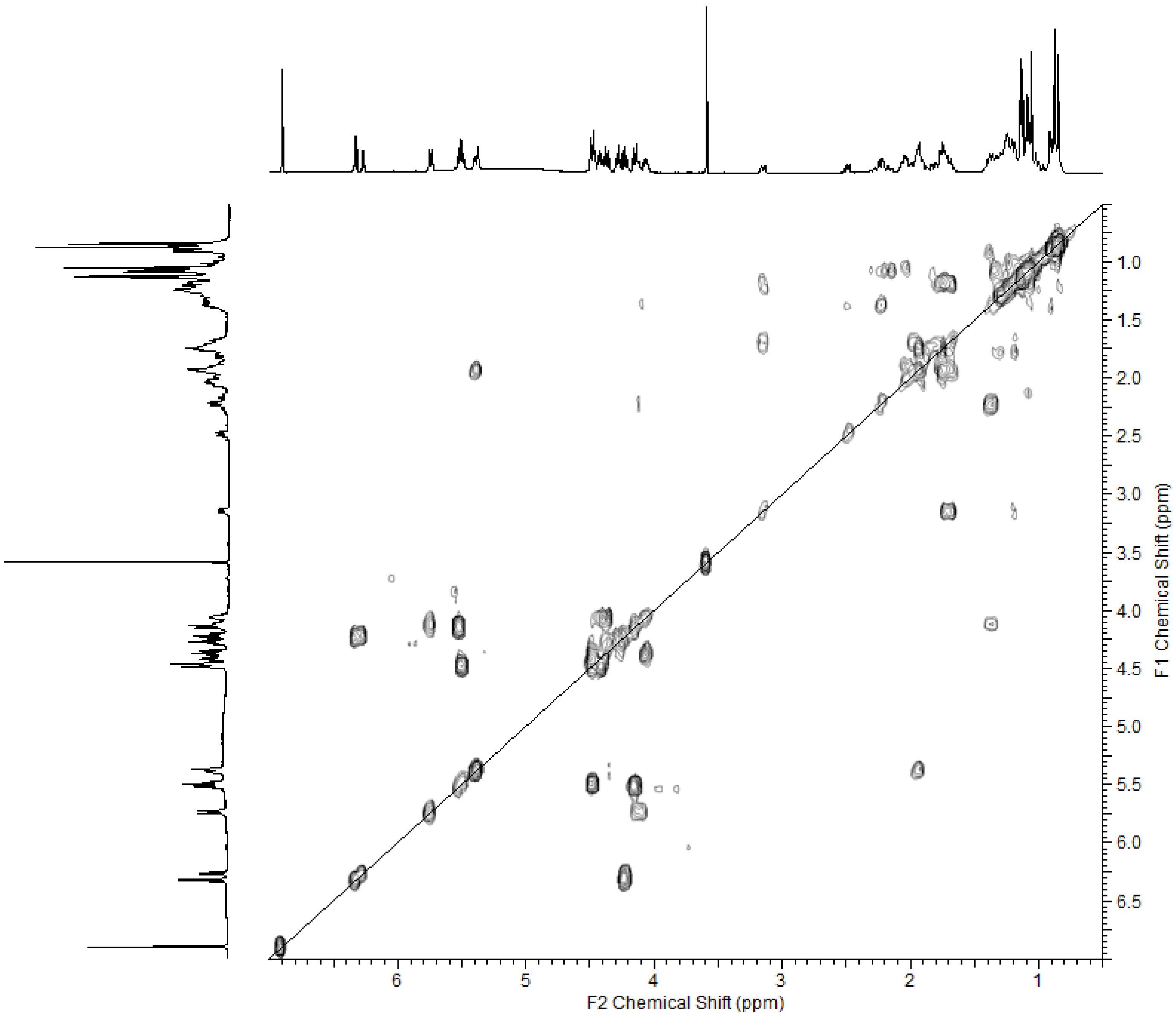

Figure S35. ^{1}H-^{1}H COSY spectrum of **11** (pyridine-d_5, 500 MHz)

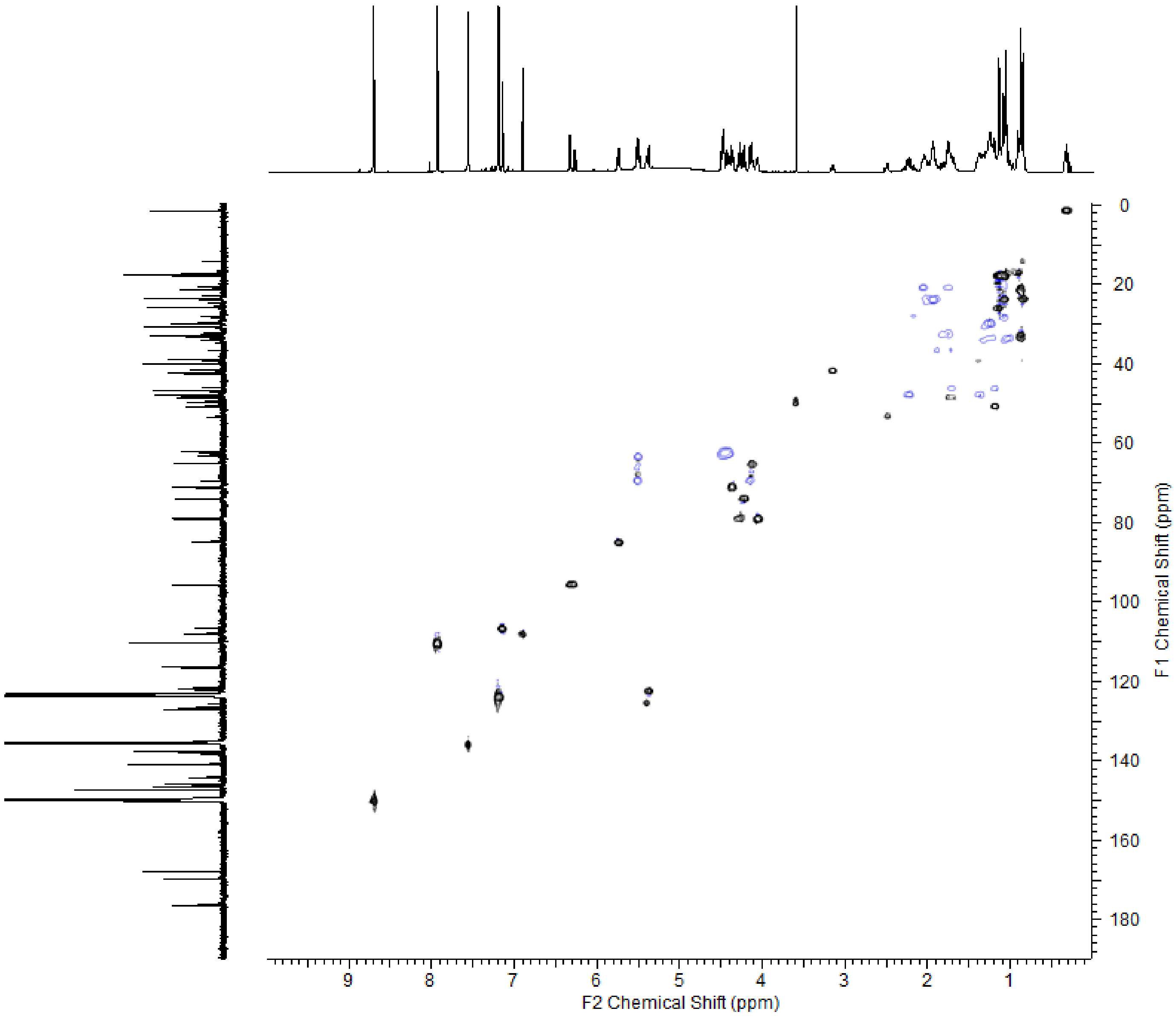

Figure S36. HSQC spectrum of **11** (pyridine-d_5)

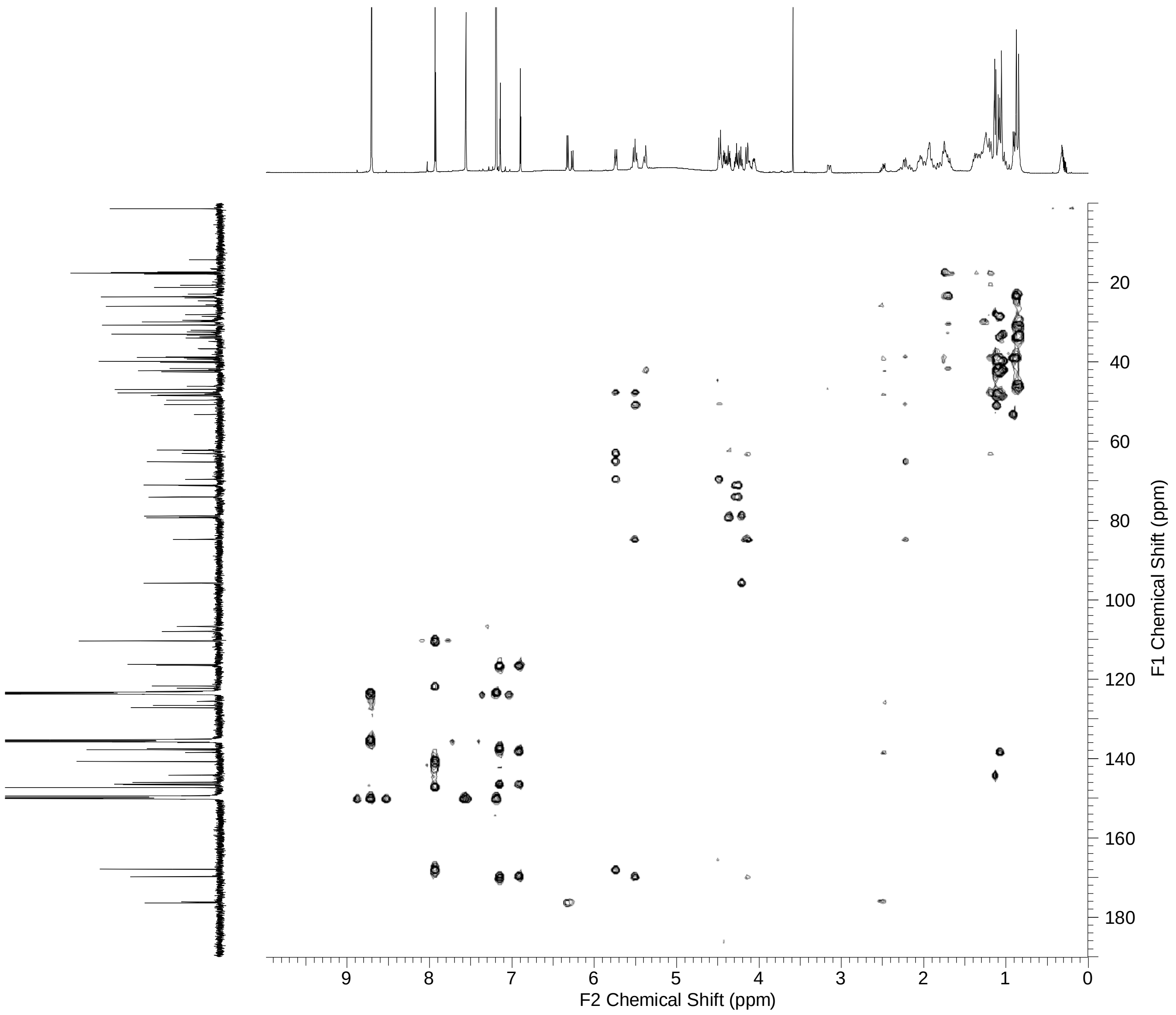

Figure S37. HMBC spectrum of **11** (pyridine-*d*$_5$)

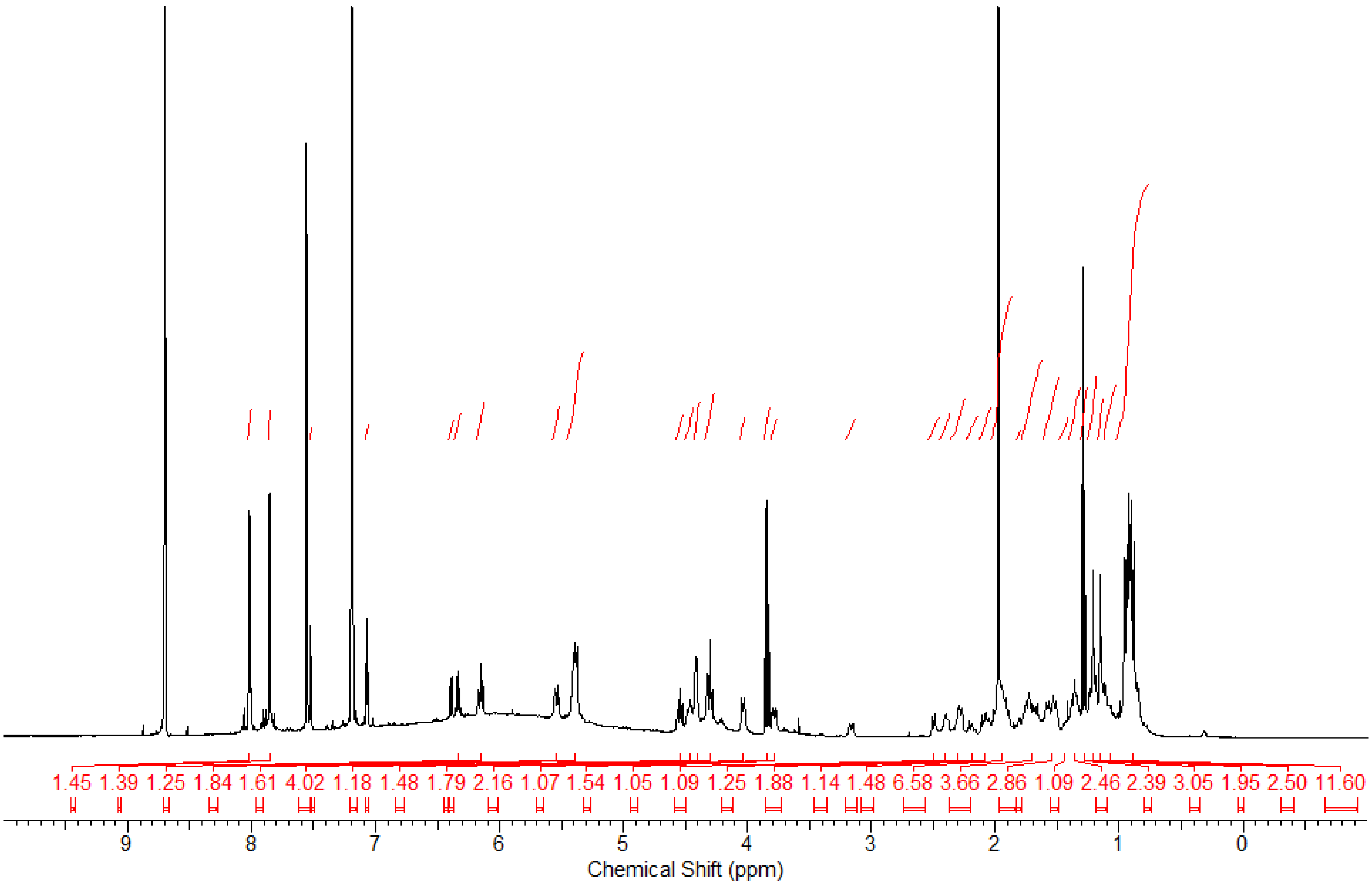

Figure S38. ¹H NMR spectrum of **12** (pyridine-d_5, 500 MHz)

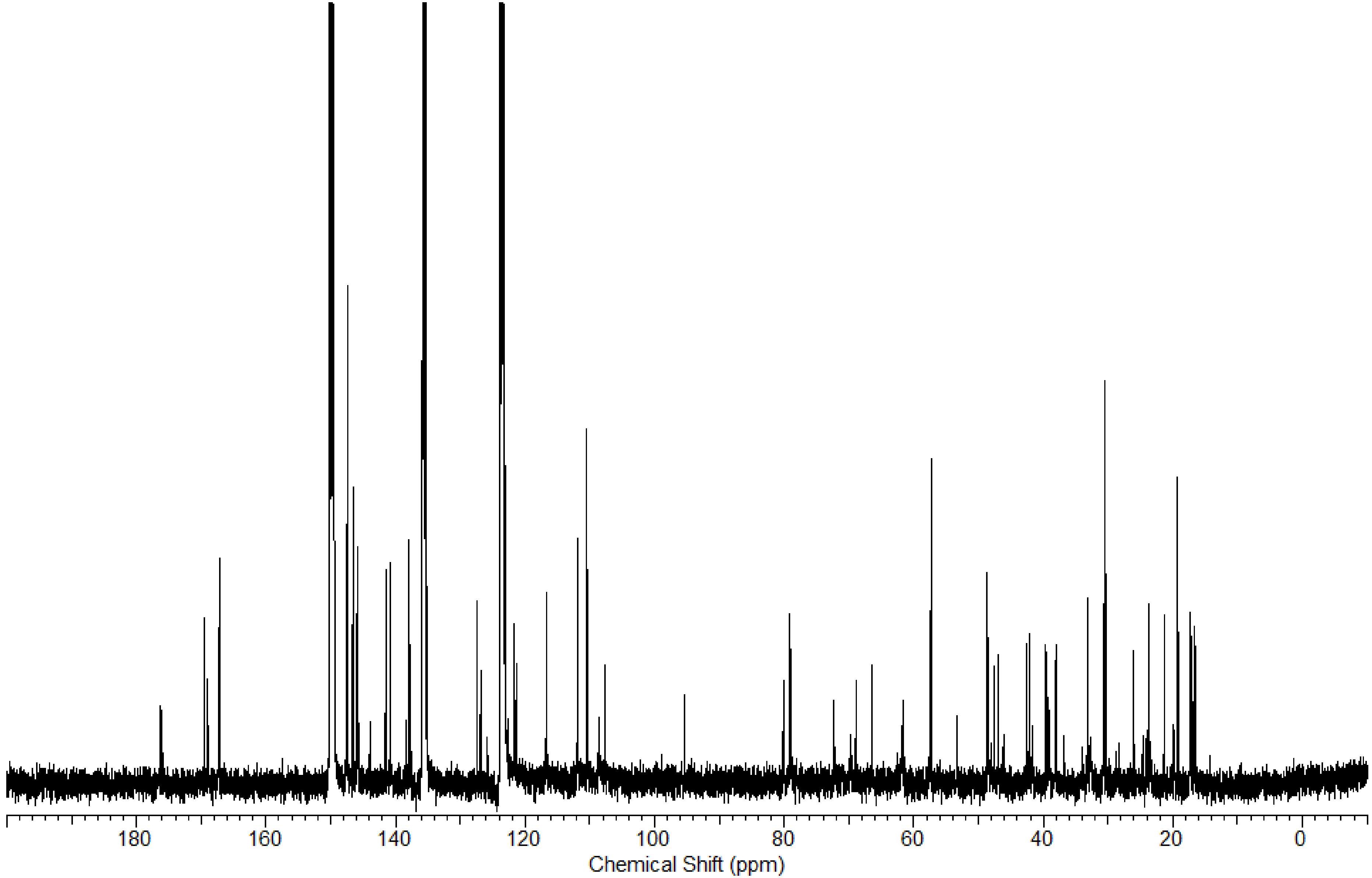

Figure S39. ^{13}C NMR spectrum of **12** (pyridine-d_5, 126 MHz)

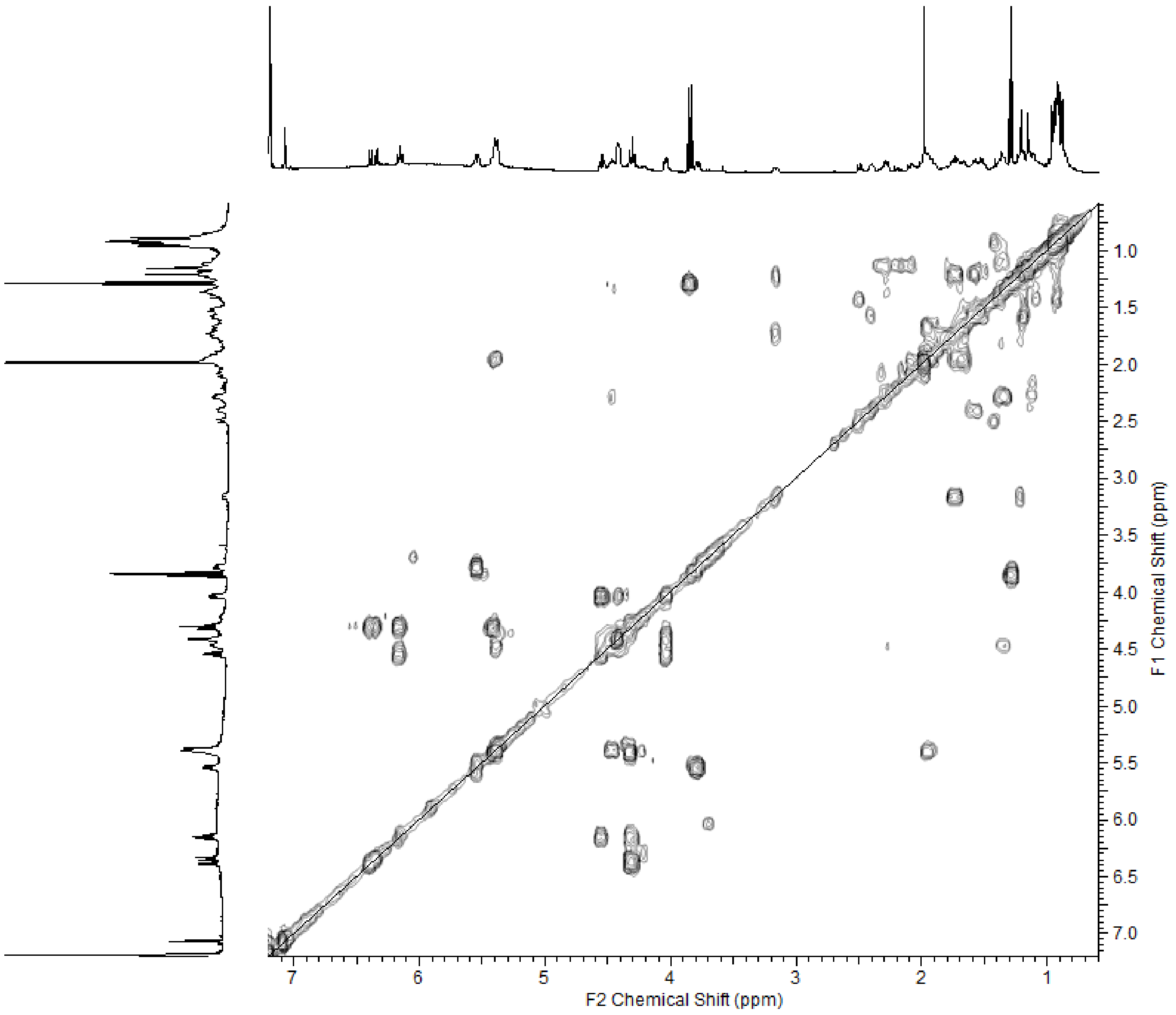

Figure S40. ^{1}H-^{1}H COSY spectrum of **12** (pyridine-d_5, 500 MHz)

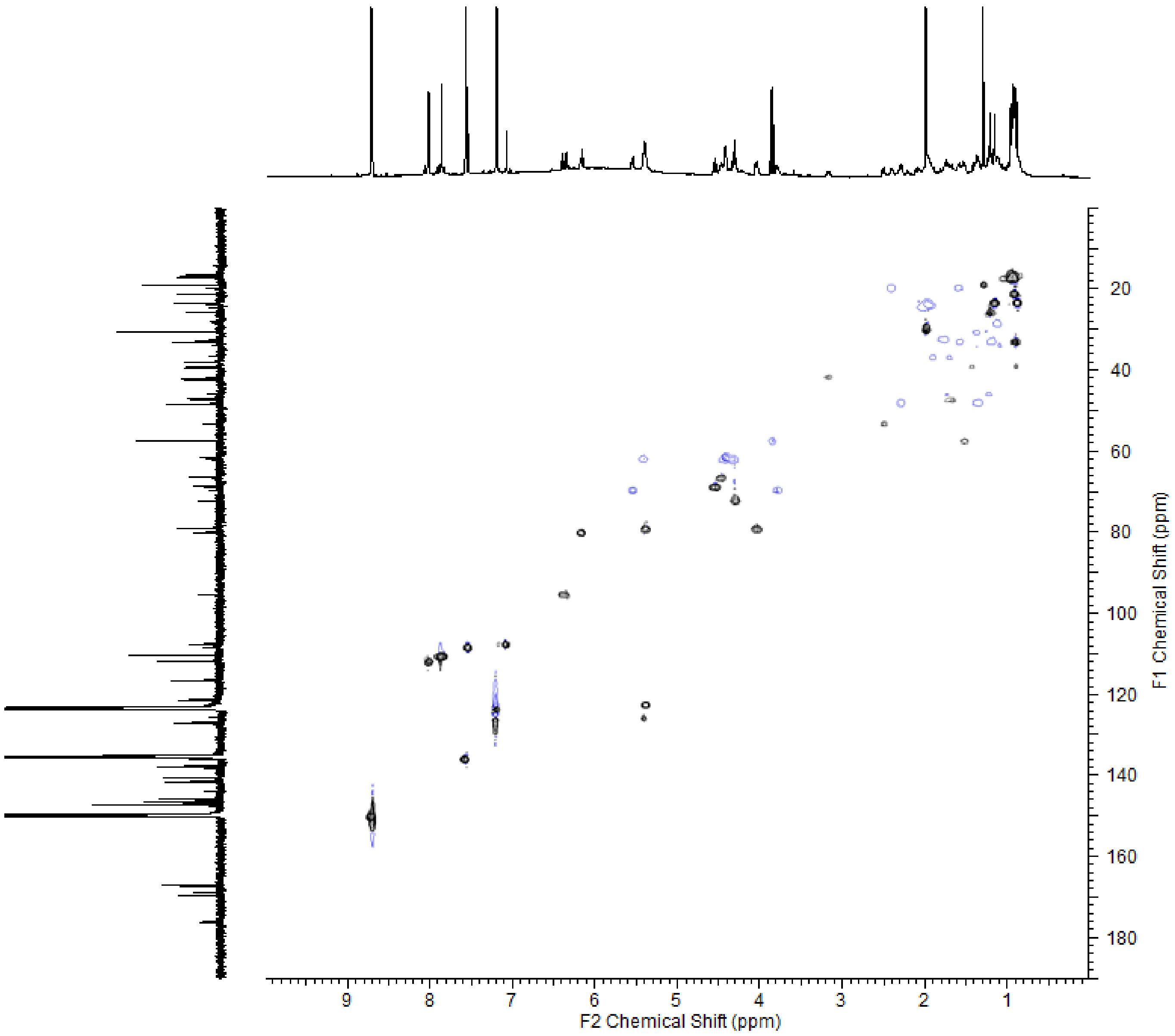

Figure S41. HSQC spectrum of **12** (pyridine-d_5)

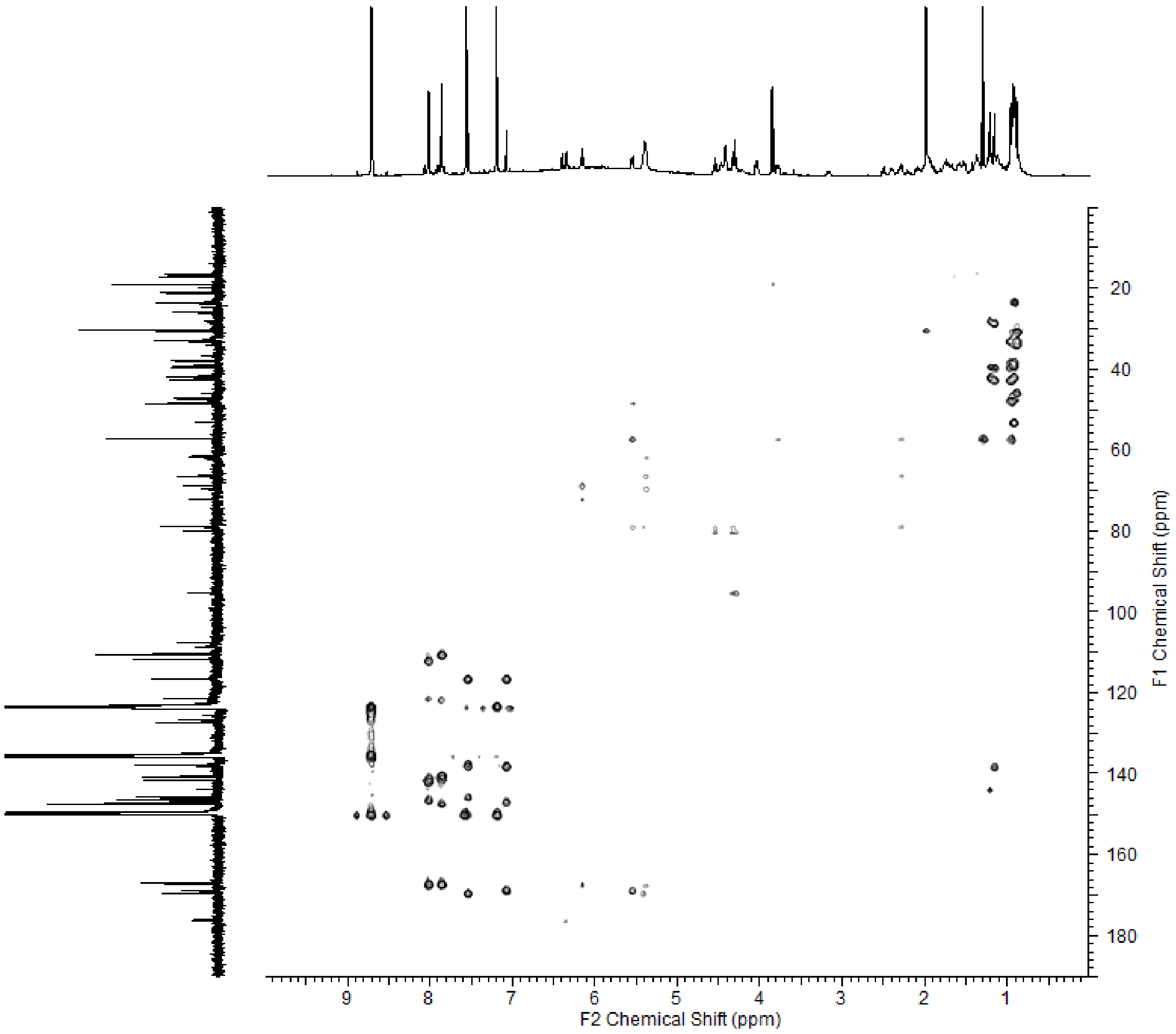

Figure S42. HMBC spectrum of **12** (pyridine-d_5)

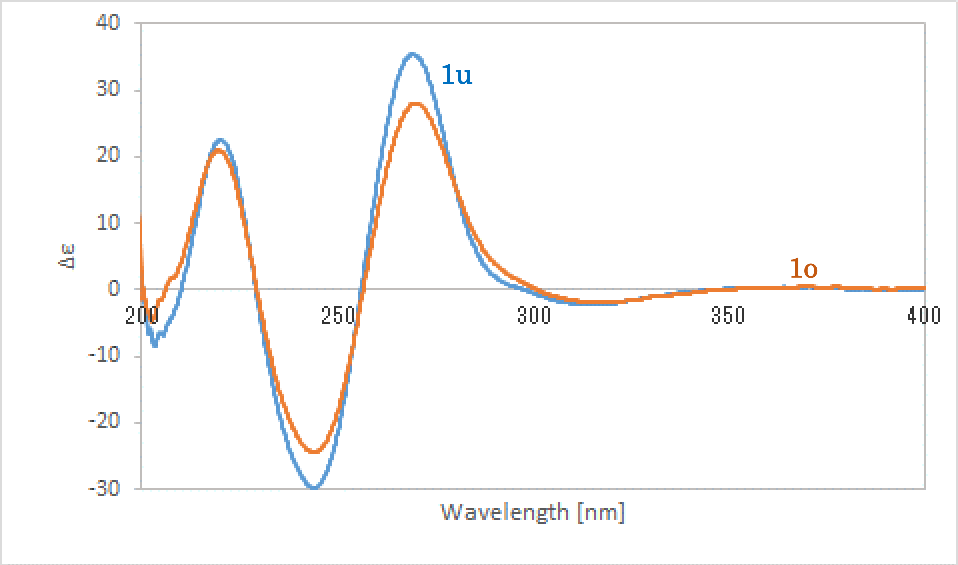

Figure S43. Experimental ECD spectra of **1o** and **1u**

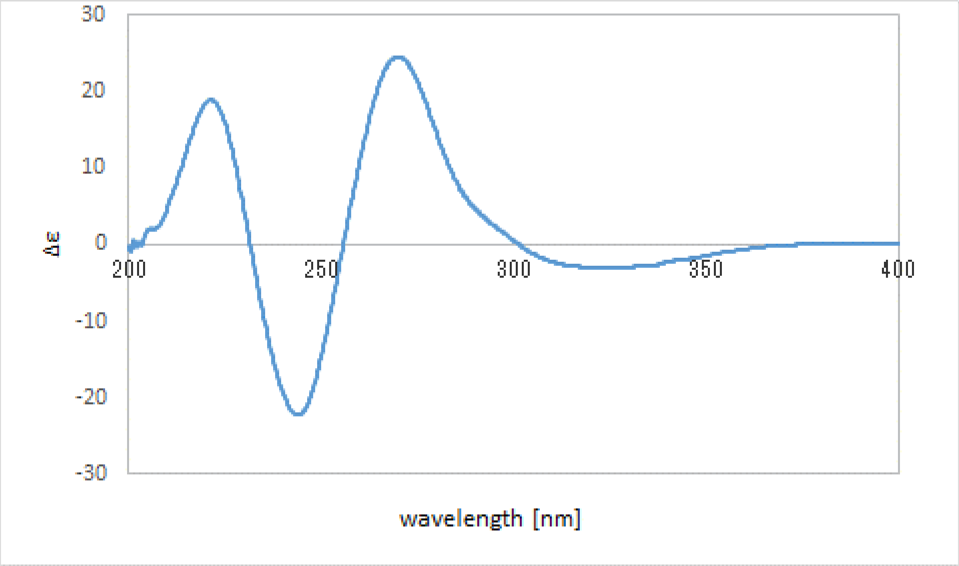

Figure S44. Experimental ECD spectra of **2o,u**

Figure S45. Lowest-energy conformers of 3,24-(*R*)-HHDP ester of methyl 2α,3β,23,24-tetrahydroxyolean-12-en-28-oate (**4'oA**) and 3,23-(*R*)-HHDP ester of methyl 2α,3β,23,24-tetrahydroxyolean-12-en-28-oate (**4'oB**) at the B3LYP/6-31G(d,p) level in pyridine.

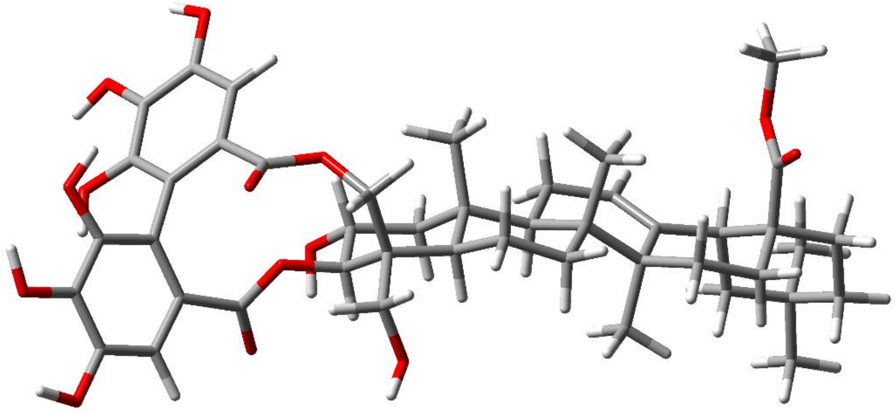

4'oA (3,24-(*R*)-HHDP ester**)**

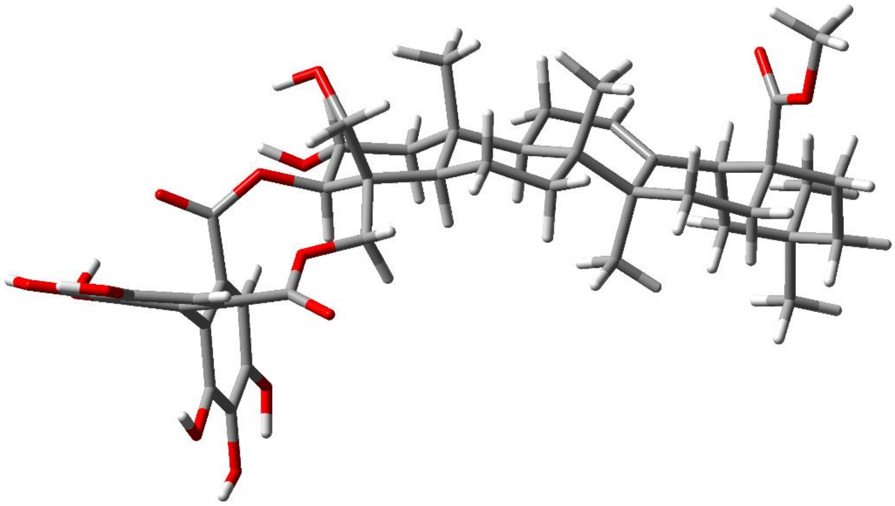

4'oB (3,23-(*R*)-HHDP ester**)**

Table S3. Calculated ^{1}H NMR chemical shifts of **4'oA**.

Position	calculated[a]																			experimental[b]
	**4oA-1**	**4oA-2**	**4oA-3**	**4oA-4**	**4oA-5**	**4oA-6**	**4oA-7**	**4oA-8**	**4oA-9**	**4oA-10**	**4oA-11**	**4oA-12**	**4oA-13**	**4oA-14**	**4oA-15**	**4oA-16**	**4oA-17**	averaged-**4oA**[c]	averaged-**4oA** (corrected)[d]	**4o**
1	2.11	2.12	2.01	1.99	2.03	2.01	2.05	1.97	2.04	2.18	2.04	2.09	2.15	2.14	1.95	2.01	2.00	2.07	2.03	2.02
	0.89	0.89	0.89	0.91	0.93	0.94	0.97	0.87	1.04	0.98	0.97	0.93	0.98	0.93	0.89	1.03	0.91	0.91	0.82	1.16
2	3.95	3.96	3.42	3.39	3.49	3.46	3.53	3.33	4.20	3.89	3.53	4.03	3.89	4.23	3.32	4.20	3.45	3.76	3.78	4.17
3	5.38	5.39	4.20	4.21	5.15	5.15	4.96	4.49	5.65	4.98	4.98	5.39	4.97	5.45	4.50	5.63	4.21	5.08	5.15	5.82
5	1.75	1.76	1.41	1.38	1.85	1.82	1.18	1.39	1.79	1.19	1.19	1.76	1.18	1.79	1.41	1.80	1.39	1.64	1.58	1.97
6	1.53	1.52	2.96	2.94	1.71	1.70	1.46	2.86	1.57	1.48	1.47	1.54	1.49	1.56	2.87	1.57	2.98	1.86	1.80	1.66
	1.54	1.54	1.64	1.61	1.54	1.52	1.54	1.63	1.55	1.56	1.56	1.54	1.58	1.58	1.65	1.56	1.60	1.56	1.50	1.34
7	1.82	1.82	1.80	1.83	1.78	1.80	1.72	1.83	1.83	1.75	1.69	1.80	1.75	1.83	1.83	1.83	1.84	1.81	1.75	1.59
	1.44	1.43	1.40	1.46	1.41	1.45	1.43	1.45	1.44	1.44	1.39	1.45	1.42	1.47	1.44	1.43	1.46	1.43	1.36	1.30
9	1.85	1.83	1.79	1.77	1.83	1.81	1.74	1.76	1.86	1.80	1.77	1.86	1.82	1.87	1.80	1.87	1.77	1.82	1.77	1.73
11	2.17	2.17	2.08	2.06	2.11	2.10	2.07	2.04	2.18	2.14	2.08	2.18	2.14	2.21	2.06	2.16	2.06	2.13	2.09	1.89
12	5.87	5.92	5.87	5.85	5.89	5.90	5.84	5.86	5.93	5.90	5.87	5.85	5.84	5.88	5.79	5.89	5.85	5.88	5.98	5.34
15	2.01	1.72	1.90	1.64	1.91	1.63	1.62	1.66	1.65	1.63	1.88	2.00	1.90	1.98	1.89	1.97	1.64	1.83	1.77	2.27
	1.18	1.26	1.14	1.23	1.14	1.22	1.22	1.22	1.26	1.21	1.13	1.16	1.13	1.15	1.15	1.22	1.24	1.20	1.12	1.05
16	2.06	2.18	2.01	2.17	2.02	2.14	2.17	2.17	2.12	2.12	2.01	2.05	2.01	2.06	2.05	2.07	2.18	2.10	2.06	1.97
	1.77	1.78	1.76	1.78	1.75	1.78	1.78	1.78	1.79	1.77	1.77	1.77	1.75	1.80	1.79	1.79	1.78	1.77	1.72	1.89
18	3.19	3.21	3.18	3.23	3.18	3.24	3.27	3.26	3.24	3.28	3.22	3.20	3.22	3.20	3.21	3.21	3.23	3.21	3.21	3.13
19	1.81	1.82	1.81	1.81	1.82	1.83	1.80	1.76	1.80	1.75	1.83	1.80	1.75	1.80	1.83	1.86	1.81	1.81	1.76	1.63
	1.23	1.27	1.18	1.27	1.18	1.26	1.27	1.26	1.27	1.26	1.21	1.24	1.23	1.25	1.25	1.24	1.27	1.24	1.16	1.16
21	1.52	1.53	1.53	1.54	1.53	1.51	1.53	1.56	1.51	1.54	1.52	1.52	1.51	1.54	1.50	1.50	1.54	1.53	1.46	1.30
	1.36	1.35	1.38	1.25	1.38	1.30	1.25	1.33	1.31	1.28	1.34	1.35	1.29	1.34	1.27	1.33	1.25	1.34	1.26	1.04
22	1.74	1.72	1.74	1.71	1.74	1.71	1.71	1.75	1.76	1.72	1.70	1.75	1.72	1.76	1.69	1.72	1.71	1.73	1.67	1.63
23	3.56	3.55	3.47	3.44	3.54	3.54	4.65	3.50	3.46	4.16	4.66	3.61	4.17	3.68	3.51	3.46	3.44	3.61	3.62	
	3.69	3.68	3.99	3.99	4.00	3.97	3.40	4.13	3.68	3.30	3.44	3.71	3.30	3.76	4.16	3.68	4.00	3.77	3.79	
24	4.58	4.58	5.34	5.33	5.15	5.16	5.32	5.25	4.53	4.97	5.31	4.61	4.98	4.62	5.24	4.52	5.29	4.87	4.93	
	4.15	4.17	4.71	4.70	4.43	4.42	4.58	4.65	4.17	4.62	4.59	4.12	4.61	4.18	4.65	4.18	4.68	4.35	4.39	
25	1.31	1.33	0.86	0.86	0.87	0.88	0.84	0.85	1.37	1.32	0.83	1.30	1.29	1.37	0.85	1.35	0.84	1.14	1.06	1.12
26	0.99	1.03	0.89	0.92	0.89	0.94	0.91	0.92	1.06	1.01	0.86	0.98	0.95	1.03	0.87	1.02	0.92	0.97	0.88	1.16
27	1.26	1.26	1.22	1.24	1.20	1.22	1.23	1.24	1.27	1.25	1.20	1.25	1.22	1.26	1.25	1.27	1.25	1.24	1.17	1.09
29	0.99	0.99	0.99	0.99	0.99	0.99	0.98	0.96	0.98	0.97	1.01	0.99	0.97	0.99	0.99	1.00	0.99	0.99	0.90	0.84
30	1.05	1.00	1.05	1.02	1.05	1.02	1.02	1.00	1.02	1.01	1.06	1.05	1.04	1.06	1.06	1.03	1.02	1.03	0.95	0.84
HHDP 3	7.49	7.49	7.74	7.73	7.75	7.74	7.81	7.62	7.74	7.62	7.81	7.14	7.61	7.22	7.62	7.71	7.67	7.59	7.75	7.72
HHDP 3'	7.35	7.36	7.06	7.07	6.98	6.98	7.05	7.11	7.38	7.51	7.04	7.09	7.50	7.53	7.12	7.36	6.84	7.23	7.38	7.08

[a] *Calculated using the GIAO method at the mPW1PW91/6-311+G(2d,p) level in pyridine (PCM).* [b] *Measured in pyridine-d_5.* [c] *Averaged according to the Boltzmann distribution theory at 298 K based on relative Gibbs free energies.* [d] *Linearly corrected for the experimental data.*

Table S4. Calculated ^{1}H NMR chemical shifts of **4'oB**.

Position	calculated[a]																					experimental[b]
	**4oB-1**	**4oB-2**	**4oB-3**	**4oB-4**	**4oB-5**	**4oB-6**	**4oB-7**	**4oB-8**	**4oB-9**	**4oB-10**	**4oB-11**	**4oB-12**	**4oB-13**	**4oB-14**	**4oB-15**	**4oB-16**	**4oB-17**	**4oB-18**	**4oB-19**	averaged-**4oB**[c]	averaged-**4oB** (corrected)[d]	**4o**
1	2.29	2.26	2.21	2.27	2.20	2.21	2.15	2.20	2.19	2.09	2.24	2.32	2.30	2.16	2.17	2.17	2.38	2.26	2.22	2.23	2.18	2.02
	1.19	1.19	1.19	1.20	1.19	1.19	1.19	1.22	1.19	1.23	1.16	1.19	1.20	1.15	1.20	1.11	0.98	1.16	1.15	1.19	1.15	1.16
2	4.62	4.44	4.50	4.46	4.44	4.66	4.51	4.54	4.46	4.61	4.30	4.29	4.68	4.49	4.54	4.14	4.21	4.44	4.50	4.51	4.43	4.17
3	5.17	5.21	5.13	5.19	5.20	5.08	5.13	5.16	5.20	5.09	5.06	5.08	5.17	5.09	5.16	4.98	4.93	5.15	5.09	5.15	5.07	5.82
5	1.12	1.17	1.18	1.17	1.15	1.12	1.17	1.19	1.16	1.11	1.06	1.10	1.13	1.17	1.18	1.01	1.19	1.16	1.18	1.15	1.12	1.97
6	1.63	1.50	1.51	1.51	1.52	1.63	1.53	1.52	1.54	1.65	2.03	2.02	1.64	1.54	1.54	1.92	1.51	1.51	1.52	1.58	1.54	1.66
	1.21	1.31	1.33	1.35	1.32	1.22	1.33	1.40	1.35	1.22	1.42	1.43	1.23	1.35	1.38	1.60	1.43	1.36	1.34	1.31	1.27	1.34
7	1.68	1.65	1.65	1.66	1.61	1.68	1.63	1.68	1.63	1.53	1.44	1.47	1.69	1.63	1.66	1.52	1.67	1.66	1.65	1.64	1.60	1.59
	1.43	1.39	1.40	1.40	1.31	1.43	1.32	1.41	1.34	1.36	1.28	1.33	1.44	1.34	1.39	1.33	1.38	1.40	1.41	1.38	1.35	1.30
9	1.83	1.81	1.79	1.83	1.83	1.80	1.80	1.79	1.86	1.89	1.85	1.84	1.84	1.79	1.82	1.85	1.75	1.82	1.77	1.82	1.78	1.73
11	2.19	2.14	2.11	2.15	2.13	2.17	2.11	2.13	2.16	2.21	2.19	2.21	2.19	2.11	2.14	2.16	2.14	2.14	2.11	2.15	2.10	1.89
12	5.90	5.87	5.86	5.88	5.79	5.90	5.78	5.89	5.81	5.75	5.81	5.88	5.91	5.78	5.84	5.78	5.92	5.87	5.86	5.86	5.76	5.34
15	1.70	1.66	1.66	1.67	1.87	1.71	1.85	1.68	1.90	2.08	1.88	1.63	1.71	1.84	1.86	1.90	1.65	1.66	1.67	1.74	1.70	2.27
	1.19	1.20	1.21	1.19	1.05	1.20	1.05	1.21	1.04	1.04	1.05	1.17	1.20	1.06	1.08	1.05	1.22	1.20	1.22	1.15	1.12	1.05
16	2.15	2.14	2.14	2.14	2.02	2.15	2.01	2.15	2.03	2.05	2.00	2.16	2.15	2.02	2.05	2.01	2.14	2.14	2.14	2.11	2.06	1.97
	1.80	1.78	1.79	1.77	1.72	1.80	1.72	1.76	1.75	1.75	1.72	1.82	1.78	1.73	1.79	1.73	1.76	1.77	1.79	1.77	1.73	1.89
18	3.23	3.21	3.21	3.21	3.20	3.22	3.20	3.23	3.23	3.17	3.18	3.24	3.21	3.20	3.22	3.19	3.24	3.18	3.21	3.21	3.15	3.13
19	2.15	2.11	2.10	2.13	2.11	2.14	2.09	2.12	2.13	2.21	2.16	2.20	2.16	2.10	2.13	2.18	2.12	2.13	2.10	2.13	2.08	1.63
	1.19	1.20	1.21	1.19	1.05	1.20	1.05	1.21	1.04	1.04	1.05	1.17	1.20	1.06	1.08	1.05	1.22	1.20	1.22	1.15	1.12	1.16
21	1.53	1.52	1.52	1.53	1.47	1.53	1.46	1.52	1.47	1.46	1.47	1.51	1.54	1.46	1.50	1.47	1.52	1.54	1.53	1.51	1.47	1.30
	1.30	1.29	1.29	1.30	1.32	1.30	1.31	1.32	1.31	1.31	1.33	1.29	1.30	1.31	1.30	1.33	1.33	1.30	1.29	1.30	1.27	1.04
22	1.69	1.69	1.69	1.67	1.71	1.69	1.69	1.67	1.68	1.72	1.70	1.70	1.68	1.69	1.67	1.71	1.70	1.67	1.69	1.69	1.65	1.63
23	3.20	3.55	3.54	3.47	3.55	3.23	3.56	3.52	3.44	3.24	3.19	3.20	3.20	3.53	3.49	3.22	3.51	3.46	3.51	3.41	3.35	
	4.48	5.01	5.13	5.56	5.02	4.47	5.10	5.57	5.61	4.48	5.30	5.32	4.82	5.18	5.61	5.67	5.54	5.53	5.17	5.03	4.95	
24	3.74	3.58	3.61	3.62	3.59	3.74	3.62	3.63	3.64	3.76	4.12	4.13	3.83	3.62	3.65	3.99	3.61	3.62	3.61	3.68	3.61	
	3.70	4.41	4.41	4.45	4.41	3.69	4.41	4.45	4.43	3.71	4.02	4.00	3.79	4.39	4.43	3.91	4.40	4.43	4.39	4.18	4.11	
25	1.48	0.98	0.97	1.02	0.94	1.49	0.93	1.00	0.98	1.43	1.20	1.27	1.50	0.93	0.99	1.18	1.01	1.00	0.97	1.14	1.10	1.12
26	1.05	0.85	0.85	0.86	0.77	1.03	0.77	0.87	0.77	0.77	0.79	0.87	1.05	0.78	0.81	0.82	0.92	0.85	0.85	0.88	0.85	1.16
27	1.24	1.21	1.21	1.21	1.18	1.23	1.19	1.21	1.22	1.22	1.17	1.19	1.24	1.19	1.22	1.18	1.19	1.20	1.21	1.21	1.18	1.09
29	0.98	0.97	0.97	0.97	0.98	0.98	0.98	0.97	0.99	0.99	0.97	0.98	0.98	0.98	0.98	0.98	0.98	0.97	0.97	0.98	0.95	0.84
30	1.02	1.01	1.01	1.01	1.06	1.02	1.06	1.01	1.06	1.08	1.06	1.02	1.01	1.06	1.05	1.06	1.00	1.01	1.01	1.03	1.00	0.84
HHDP 3	7.71	7.76	7.79	7.85	7.74	7.82	7.80	8.22	7.80	7.84	7.51	7.49	7.83	7.64	8.17	7.37	8.07	7.71	7.63	7.79	7.67	7.72
HHDP 3'	7.53	7.58	7.62	7.57	7.59	7.54	7.63	7.57	7.57	7.54	7.51	7.50	7.36	7.58	7.58	8.13	7.59	7.41	7.56	7.57	7.46	7.08

[a] Calculated using the GIAO method at the mPW1PW91/6-311+G(2d,p) level in pyridine (PCM). [b] Measured in pyridine-d_5. [c] Averaged according to the Boltzmann distribution theory at 298 K based on relative Gibbs free energies. [d] Linearly corrected for the experimental data.

Table S5. Calculated ^{13}C NMR chemical shifts of **4'oA**.

Position	calculated[a]																			experimental[b]
	**4oA-1**	**4oA-2**	**4oA-3**	**4oA-4**	**4oA-5**	**4oA-6**	**4oA-7**	**4oA-8**	**4oA-9**	**4oA-10**	**4oA-11**	**4oA-12**	**4oA-13**	**4oA-14**	**4oA-15**	**4oA-16**	**4oA-17**	averaged-**4oA**[c]	averaged-**4oA** (corrected)[d]	**4o**
1	45.5	45.5	44.7	44.6	44.7	44.6	44.1	44.7	45.8	45.1	44.0	45.5	45.1	45.4	44.5	45.7	44.5	45.1	46.8	48.3
2	68.4	68.4	67.7	67.6	67.4	67.4	67.5	68.2	67.3	67.6	67.6	68.4	67.6	68.3	68.1	67.3	67.4	68.0	69.0	67.4
3	77.4	77.4	81.2	81.2	79.7	79.7	84.4	80.5	75.1	84.0	84.4	77.5	84.1	78.2	80.3	75.1	81.5	79.0	79.7	79.1
4	48.5	48.5	50.4	50.2	49.6	49.7	47.8	49.7	48.6	48.7	47.9	48.5	48.7	48.6	49.9	48.5	50.1	49.0	50.5	48.3
5	44.0	44.1	55.5	55.6	44.2	44.2	52.2	55.6	44.1	50.8	52.1	44.0	50.7	44.0	55.5	44.0	55.4	47.2	48.8	47.0
6	16.0	16.0	16.9	16.9	15.7	15.6	16.7	16.8	16.3	16.9	16.7	16.0	16.9	16.0	16.8	16.3	16.9	16.2	18.8	18.2
7	30.0	30.7	30.2	31.3	29.9	30.9	31.3	31.2	30.9	31.5	30.3	30.0	30.5	30.3	30.4	29.9	31.2	30.5	32.6	32.7
8	41.0	40.7	40.2	40.1	40.5	40.2	40.2	40.3	40.7	40.7	39.9	41.1	40.6	41.0	40.1	40.7	40.1	40.6	42.4	39.9
9	45.1	45.2	45.8	45.8	45.9	45.9	45.7	45.9	45.3	45.5	45.7	44.9	45.5	45.0	45.9	45.3	45.7	45.4	47.1	47.5
10	39.1	39.2	39.0	39.0	38.5	38.7	38.6	39.0	38.9	39.9	38.6	39.2	39.8	39.3	39.1	38.6	38.9	39.0	40.9	37.7
11	22.4	22.3	22.4	22.4	22.4	22.4	22.4	22.4	22.3	22.0	22.4	22.4	22.0	22.4	22.4	22.4	22.4	22.4	24.7	24.0
12	127.4	127.8	127.0	127.3	127.2	127.4	127.4	127.6	127.8	127.9	127.0	127.3	127.5	127.6	126.7	127.5	127.3	127.4	126.6	122.7
13	150.5	149.6	150.5	149.6	150.4	149.6	149.5	149.5	149.9	149.9	150.3	150.8	150.6	150.6	150.5	150.2	149.7	150.1	148.6	144.1
14	42.9	43.7	43.6	43.8	43.7	44.1	43.7	43.7	44.2	43.9	43.7	42.8	43.3	43.4	43.5	43.4	43.7	43.5	45.2	42.2
15	24.8	25.5	24.3	25.3	24.3	25.3	25.4	25.2	25.0	25.3	24.4	24.7	24.4	24.6	24.5	24.6	25.4	25.0	27.2	28.1
16	20.7	21.3	20.6	21.4	20.6	21.5	21.4	21.3	21.4	21.5	20.7	20.7	20.7	20.7	20.8	20.8	21.4	21.0	23.4	23.4
17	48.7	48.2	48.6	48.0	48.6	47.9	47.8	48.4	48.4	48.1	48.0	48.5	48.5	48.6	48.2	49.2	48.0	48.4	49.9	47.0
18	41.5	41.1	41.2	41.2	41.3	41.0	41.1	41.1	41.3	41.2	41.3	41.4	41.6	41.6	41.5	41.4	41.2	41.3	43.1	41.7
19	43.1	43.2	43.8	42.9	43.8	43.1	43.0	42.9	42.8	42.9	43.6	43.0	42.8	43.0	43.0	43.4	42.9	43.2	44.9	46.1
20	31.4	31.1	31.2	31.2	31.1	31.1	31.2	31.8	31.3	31.1	31.1	31.4	31.0	31.1	31.3	31.1	31.1	31.2	33.3	30.7
21	31.1	31.3	30.8	31.5	30.8	31.3	31.6	31.7	31.4	31.4	30.8	31.1	31.1	31.2	31.1	30.9	31.5	31.2	33.2	33.9
22	31.0	30.7	31.1	31.0	31.1	30.8	30.8	30.8	31.0	30.7	31.0	31.0	30.9	31.0	31.1	31.0	30.9	30.9	33.0	33.0
23	60.1	60.1	69.0	69.0	56.8	56.8	71.2	68.9	60.0	73.4	71.2	60.2	73.5	60.3	68.9	60.0	69.3	62.7	63.8	62.2
24	60.3	60.4	71.2	71.4	70.4	70.4	72.9	70.1	60.9	62.1	72.9	59.9	62.2	59.8	70.0	60.9	71.4	64.7	65.7	62.4
25	10.3	10.3	11.3	11.3	11.5	11.5	10.8	11.2	10.3	10.9	11.0	10.4	10.9	10.5	11.3	10.3	11.2	10.7	13.4	17.5
26	12.6	13.3	12.5	13.2	12.5	13.2	13.3	13.3	13.7	13.6	12.4	12.5	12.7	12.8	12.3	12.9	13.2	12.9	15.6	17.7
27	20.9	20.4	20.7	20.2	20.7	20.2	20.1	20.1	20.5	20.3	20.6	21.0	20.8	20.7	20.8	20.9	20.2	20.6	23.0	25.9
28	184.0	183.9	183.9	183.8	183.9	183.9	183.8	183.9	184.0	183.7	183.8	184.0	183.7	183.9	183.7	184.0	183.8	183.9	181.4	176.4
29	28.5	28.5	28.6	28.3	28.6	28.4	28.2	28.3	28.3	28.3	28.6	28.4	28.3	28.4	28.3	28.4	28.3	28.4	30.6	33.0
30	18.7	18.6	18.7	18.6	18.7	18.6	18.6	18.6	18.6	18.6	18.7	18.7	18.7	18.7	18.7	18.7	18.6	18.6	21.1	23.6
HHDP-1	110.5	110.5	116.8	116.6	116.8	116.6	116.5	113.9	111.3	110.4	116.7	117.3	110.3	117.5	114.1	111.2	120.7	113.2	112.8	118.4
HHDP-1'	109.3	109.1	116.0	116.1	115.8	115.9	115.9	116.6	110.3	110.4	116.0	116.9	110.3	119.4	116.5	110.1	118.6	112.4	112.0	118.0
HHDP-2	128.0	128.0	125.5	125.5	125.8	125.9	126.1	124.6	126.9	127.5	126.0	126.5	127.5	127.1	124.5	127.0	125.0	126.9	126.1	
HHDP-2'	131.2	131.2	124.7	124.5	125.1	124.8	124.3	123.7	131.7	129.8	124.7	129.4	129.6	128.9	124.1	131.8	124.7	128.5	127.6	
HHDP-3	111.7	111.7	113.3	113.4	113.0	113.2	113.7	110.7	113.3	113.3	113.7	107.2	113.2	110.0	110.5	113.3	112.5	112.2	111.9	112.1
HHDP-3'	107.5	107.5	104.3	104.3	104.0	104.0	104.0	106.9	107.3	108.7	104.2	104.0	108.7	110.0	106.9	107.3	101.7	106.3	106.1	106.6
HHDP-4	146.6	146.6	146.6	147.1	146.6	147.0	147.1	144.0	146.3	146.5	146.7	143.1	146.6	143.1	143.9	146.3	145.9	146.4	145.0	145.7
HHDP-4'	147.3	147.2	145.8	145.9	145.8	145.9	145.9	145.3	146.8	147.1	145.8	143.4	147.1	145.6	145.1	147.0	143.2	146.5	145.1	145.9
HHDP-5	135.9	136.0	141.1	141.8	141.1	141.5	141.7	137.2	136.4	136.7	141.0	135.8	136.8	135.7	137.2	136.3	140.9	137.9	136.8	139.8
HHDP-5'	135.6	135.5	135.0	135.5	134.7	135.4	135.4	135.4	134.6	136.2	135.0	135.2	136.3	140.0	134.8	134.9	135.5	135.5	134.4	137.7
HHDP-6	143.2	143.3	146.0	146.1	146.1	146.2	146.0	146.2	143.0	143.3	145.8	146.6	143.4	143.3	146.1	142.8	144.5	144.4	143.1	147.1
HHDP-6'	144.0	143.9	143.9	143.7	143.8	144.1	143.6	143.7	143.0	143.6	143.6	146.5	143.6	143.7	143.5	143.3	146.9	143.9	142.6	146.9
HHDP-7	173.6	173.7	174.3	174.4	174.7	174.7	175.0	173.6	170.9	174.6	174.9	175.4	174.8	174.7	173.7	171.0	175.7	174.0	171.8	168.9
HHDP-7'	173.9	173.9	178.5	178.6	179.3	179.2	179.1	174.4	174.0	175.8	179.0	174.1	175.8	173.8	174.3	174.0	178.1	175.8	173.5	170.7

[a] *Calculated using the GIAO method at the mPW1PW91/6-311+G(2d,p) level in pyridine (PCM).* [b] *Measured in pyridine-d_5.* [c] *Averaged according to the Boltzmann distribution theory at 298 K based on relative Gibbs free energies.* [d] *Linearly corrected for the experimental data.*

Table S6. Calculated ^{13}C NMR chemical shifts of **4'oB**.

Position	calculated[a]																					experimental[b]
	**4oB-1**	**4oB-2**	**4oB-3**	**4oB-4**	**4oB-5**	**4oB-6**	**4oB-7**	**4oB-8**	**4oB-9**	**4oB-10**	**4oB-11**	**4oB-12**	**4oB-13**	**4oB-14**	**4oB-15**	**4oB-16**	**4oB-17**	**4oB-18**	**4oB-19**	averaged-**4oB**[c]	averaged-**4oB** (corrected)[d]	**4o**
1	42.1	41.4	44.3	41.2	41.3	45.1	44.2	44.8	41.0	45.1	41.8	42.0	42.1	44.3	44.6	40.7	44.6	41.3	44.5	42.8	43.9	48.3
2	68.7	67.5	67.7	67.5	67.5	69.0	67.7	67.9	67.5	68.7	67.5	67.6	68.6	68.0	67.9	67.7	68.7	67.6	67.9	68.0	68.4	67.4
3	92.7	91.2	88.5	90.1	91.4	89.4	88.6	87.2	90.2	89.4	90.4	90.4	91.3	88.5	87.2	89.0	89.6	89.8	88.3	90.2	90.0	79.1
4	48.7	47.7	47.5	47.6	47.6	48.6	47.4	47.7	47.4	48.6	47.2	47.4	48.1	47.4	47.6	47.9	48.7	47.5	47.5	47.9	48.9	48.3
5	51.8	53.1	52.4	52.9	52.7	51.5	52.2	52.3	52.8	51.2	52.4	52.8	51.6	52.2	52.4	51.8	52.2	52.9	52.5	52.3	53.2	47.0
6	18.3	17.1	17.2	17.1	17.2	18.4	17.3	17.1	17.0	18.7	19.6	19.7	18.0	17.2	17.1	19.8	17.2	17.0	17.2	17.6	19.5	18.2
7	31.6	31.3	31.2	31.4	29.8	31.6	29.9	31.4	29.8	28.8	30.2	31.5	31.6	29.9	30.4	30.1	31.4	31.4	31.2	30.9	32.4	32.7
8	40.9	40.4	40.4	40.5	40.0	40.9	40.1	40.5	40.4	40.8	40.0	40.1	40.9	40.2	40.6	40.0	40.3	40.6	40.5	40.5	41.7	39.9
9	45.3	46.5	46.2	46.6	46.4	45.2	46.2	46.4	46.6	45.7	46.7	46.7	45.4	46.2	46.3	47.0	46.2	46.6	46.3	46.1	47.1	47.5
10	38.4	38.6	38.3	38.5	38.5	38.2	38.3	38.2	38.5	38.0	38.3	38.6	38.5	38.2	38.1	38.8	38.8	38.6	38.3	38.4	39.6	37.7
11	22.7	22.5	22.4	22.5	22.5	22.7	22.5	22.4	22.6	22.9	22.4	22.3	22.7	22.5	22.6	22.3	22.2	22.5	22.4	22.6	24.2	24.0
12	127.6	127.1	127.0	127.4	126.6	127.5	126.6	127.5	126.9	127.0	126.8	127.1	127.9	126.5	127.1	126.6	127.4	127.3	127.0	127.2	125.9	122.7
13	149.6	149.3	149.5	149.5	150.3	149.6	150.3	149.6	150.2	150.8	150.2	149.5	149.6	150.3	150.2	150.4	149.9	149.6	149.5	149.8	147.9	144.1
14	43.8	43.4	43.3	43.5	42.6	43.8	42.8	43.5	42.8	42.7	42.3	43.1	44.0	42.9	43.1	42.5	43.6	43.5	43.3	43.3	44.4	42.2
15	25.5	25.7	25.6	25.7	24.6	25.5	24.6	25.6	24.6	24.7	24.7	25.3	25.5	24.6	24.6	24.5	25.5	25.6	25.6	25.3	26.9	28.1
16	21.6	21.6	21.6	21.6	20.5	21.6	20.6	21.5	20.7	20.4	20.4	21.3	21.5	20.6	20.8	20.5	21.5	21.5	21.6	21.3	23.0	23.4
17	48.1	47.9	47.9	47.6	48.3	48.1	48.2	47.5	48.1	47.8	48.1	47.8	47.8	48.2	47.9	48.3	47.4	47.6	47.9	48.0	49.0	47.0
18	41.3	41.0	41.0	41.2	41.3	41.2	41.3	41.2	41.3	41.5	41.3	40.9	41.4	41.3	41.4	41.3	41.2	41.1	41.0	41.2	42.4	41.7
19	42.8	43.0	43.0	43.2	43.0	42.8	42.9	43.3	42.8	42.6	43.3	43.0	43.0	42.9	43.0	43.2	43.4	43.2	43.0	43.0	44.1	46.1
20	31.6	31.7	31.7	31.4	31.5	31.6	31.6	31.2	31.8	31.9	31.4	31.8	31.4	31.7	31.7	31.4	31.2	31.4	31.7	31.6	33.0	30.7
21	31.4	31.4	31.3	31.3	30.9	31.3	31.0	31.3	30.9	30.8	30.9	31.4	31.4	31.0	31.0	30.9	31.4	31.3	31.3	31.2	32.7	33.9
22	30.7	30.7	30.7	30.9	30.9	30.7	30.9	30.9	30.9	31.0	31.1	30.8	30.9	30.9	30.7	31.0	30.7	30.9	30.7	30.8	32.3	33.0
23	71.0	73.4	72.8	72.9	73.4	71.0	72.9	73.0	72.7	71.0	68.6	68.6	71.3	72.4	72.8	68.8	73.3	72.6	72.3	72.2	72.5	62.2
24	58.3	63.4	63.4	63.8	63.4	58.3	63.4	63.8	63.8	58.3	59.3	59.4	58.7	63.4	63.8	56.3	63.4	63.7	63.4	61.7	62.3	62.4
25	10.6	11.8	11.6	11.9	11.7	10.4	11.5	11.7	11.9	10.3	10.8	11.0	10.4	11.5	11.7	11.7	11.6	11.8	11.6	11.3	13.3	17.5
26	13.6	12.6	12.6	12.5	11.4	13.5	11.6	12.6	11.5	12.0	10.8	12.2	13.5	11.7	11.8	11.3	12.9	12.5	12.6	12.5	14.5	17.7
27	20.0	20.1	20.2	20.3	20.7	20.0	20.7	20.3	21.0	22.2	20.7	20.1	20.2	20.7	20.8	20.8	20.4	20.3	20.2	20.4	22.1	25.9
28	183.8	183.8	183.8	183.6	183.5	183.8	183.4	183.7	183.6	183.9	183.5	183.8	183.7	183.4	183.6	183.4	183.8	183.7	183.8	183.7	180.8	176.4
29	28.3	28.3	28.3	28.3	28.3	28.3	28.3	28.3	28.3	28.3	28.3	28.3	28.3	28.3	28.3	28.3	28.3	28.3	28.3	28.3	29.8	33.0
30	18.7	18.7	18.7	18.6	18.8	18.7	18.8	18.6	18.8	18.9	18.8	18.7	18.6	18.8	18.8	18.8	18.6	18.6	18.7	18.7	20.5	23.6
HHDP-1	114.2	114.3	114.0	116.2	114.4	113.9	114.2	115.9	115.9	114.5	113.9	114.1	115.4	117.3	115.7	114.6	116.2	119.2	117.4	114.8	113.9	118.0
HHDP-1'	115.8	115.5	116.1	113.4	115.5	116.0	115.9	113.6	113.5	116.0	115.9	115.9	113.3	120.3	113.6	117.7	113.5	118.0	120.3	115.4	114.5	118.4
HHDP-2	126.2	126.6	127.3	123.8	126.4	126.5	127.0	124.2	123.8	126.3	126.8	127.1	124.6	127.1	124.2	127.8	123.9	124.0	127.2	126.0	124.8	
HHDP-2'	127.9	128.1	127.9	125.8	128.0	127.9	128.0	126.0	125.6	127.9	128.6	128.5	126.6	127.0	125.8	119.6	125.8	124.1	126.9	127.3	126.0	
HHDP-3	107.0	107.3	107.2	108.7	107.2	107.2	107.2	109.4	108.5	107.0	106.4	106.2	108.6	104.8	109.1	106.8	108.9	106.1	105.0	107.5	106.8	106.6
HHDP-3'	111.8	112.4	112.5	110.4	112.4	111.8	112.5	110.2	110.6	111.8	111.2	111.2	109.2	111.6	110.5	113.4	110.5	108.8	111.6	111.6	110.8	112.1
HHDP-4	145.7	146.0	145.7	145.0	145.8	145.6	145.7	145.0	144.9	145.4	145.8	145.7	144.9	143.1	144.9	142.8	144.8	142.4	143.1	145.4	143.6	145.7
HHDP-4'	147.2	147.1	146.9	143.8	147.1	147.1	147.0	143.6	144.1	147.3	147.0	147.0	144.1	145.8	143.9	145.7	143.6	143.4	145.8	146.2	144.4	145.9
HHDP-5	134.6	134.8	134.2	135.6	134.8	134.2	134.2	135.0	135.5	134.1	134.5	134.7	135.1	134.0	135.0	134.3	135.1	135.5	134.2	134.7	133.2	137.7
HHDP-5'	140.1	139.9	140.1	136.6	140.0	140.2	140.2	136.4	136.8	140.6	139.6	139.7	136.5	139.9	136.7	138.5	136.4	136.5	139.8	139.2	137.6	139.8
HHDP-6	143.3	143.2	142.7	143.5	143.2	142.9	142.7	143.1	143.3	143.4	143.3	143.8	143.5	145.3	143.0	145.5	143.2	146.3	145.5	143.3	141.6	146.9
HHDP-6'	145.6	145.5	145.5	145.3	145.6	145.6	145.7	145.4	145.4	146.0	145.6	145.5	145.8	144.3	145.4	143.2	145.2	145.6	144.3	145.5	143.7	147.1
HHDP-7	178.1	178.1	178.9	174.3	178.1	178.6	178.9	174.4	174.2	178.5	178.4	178.3	173.5	178.5	174.4	172.5	175.1	174.0	178.5	177.3	174.6	170.7
HHDP-7'	171.4	170.6	170.2	170.1	170.6	171.3	170.4	170.3	170.0	171.3	171.7	171.6	171.4	170.7	170.2	171.0	170.5	170.6	170.7	170.7	168.3	168.9

[a] *Calculated using the GIAO method at the mPW1PW91/6-311+G(2d,p) level in pyridine (PCM).* [b] *Measured in pyridine-*d_5. [c] *Averaged according to the Boltzmann distribution theory at 298 K based on relative Gibbs free energies.* [d] *Linearly corrected for the experimental data.*

Figure S46. Correlation plots of experimental ^{1}H NMR chemical shifts versus corresponding calculated ^{1}H NMR chemical shifts of **4'o**.

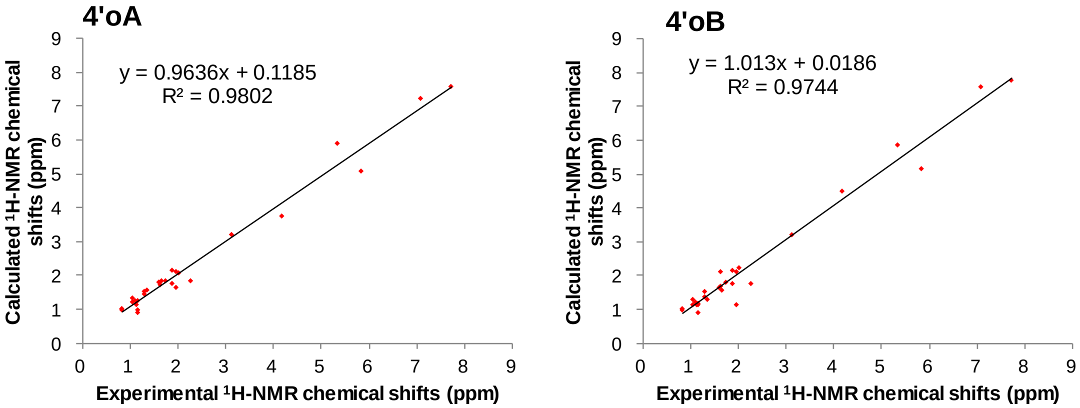

Figure S47. Correlation plots of experimental ^{13}C NMR chemical shifts versus corresponding calculated ^{13}C NMR chemical shifts of **4'o**.

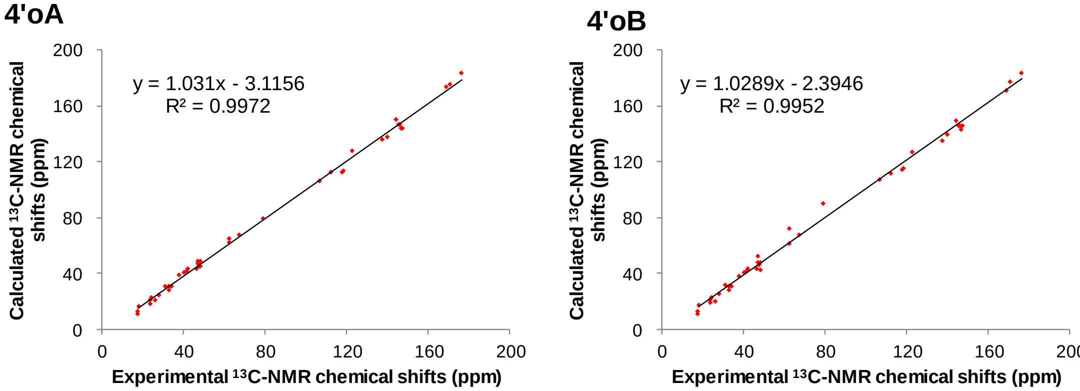

Table S7. The DP4+ probability analysis of **4'o**.

	**4'oA**	**4'oB**
sDP4+ (^{1}H)	48.77%	51.23%
sDP4+ (^{13}C)	100.00%	0.00%
sDP4+ (^{1}H + ^{13}C)	100.00%	0.00%
uDP4+ (^{1}H)	0.58%	99.42%
uDP4+ (^{13}C)	99.90%	0.10%
uDP4+ (^{1}H + ^{13}C)	85.66%	14.34%
DP4+ (^{1}H)	0.55%	99.45%
DP4+ (^{13}C)	100.00%	0.00%
DP4+ (^{1}H + ^{13}C)	100.00%	0.00%

Cartesian coordinates of the lowest-energy conformer of **4'oA** (**4'oA-1**) at the B3LYP/6-31G(d,p) level in pyridine (PCM).

C	1.50017600	0.42996600	1.09983100
C	1.93986700	-0.84574500	0.38158400
C	1.30883400	-1.09715400	-1.01920800
C	-0.24758100	-0.95309100	-0.85650800
C	-0.75792700	0.35462500	-0.16542300
C	-0.02803900	0.47625300	1.20080500
C	-1.05354800	-1.24658100	-2.13403400
C	-2.51799100	-1.53565100	-1.77671300
C	-3.22262900	-0.38821800	-1.00830800
C	-2.29724900	0.14041200	0.14474500
C	-0.45262200	1.63360600	-0.98196700
C	-4.58194200	-0.91318700	-0.32429100
C	-5.21297900	0.24060400	0.48013000
C	-4.44823300	1.21135500	0.99879100
C	-2.95999400	1.34753500	0.83722000
C	-5.56246900	-1.44622600	-1.41345600
C	-7.03512300	-1.52514100	-0.98388900
C	-7.56358800	-0.17360400	-0.46365900
C	-6.71793300	0.27375000	0.76454200
C	-9.07979200	-0.24770300	-0.09564800
C	-9.37533900	-1.03401400	1.18674300
C	-8.58661600	-0.52173400	2.41249700
C	-7.08396200	-0.52357700	2.04543800
C	-3.54704200	0.73394300	-2.02973000
C	-9.06001400	0.88501900	2.83183700
C	-8.81205000	-1.47986400	3.59616000
C	-4.33091300	-2.09395800	0.66178000
C	6.26093400	-2.65438200	0.93068600
C	5.59315000	-1.43659100	0.75646900
C	6.24469400	-0.32690300	0.15748800
C	7.56218300	-0.52889400	-0.29379300
C	8.21538500	-1.75786900	-0.15134100
C	7.56728200	-2.82344100	0.47774900
C	5.73141300	1.08439000	0.10751300
C	4.75885400	1.59105400	-0.78534600
C	4.36009200	2.92970200	-0.74622800
C	4.93965100	3.80994300	0.16582000
C	5.89458500	3.33401700	1.06817500
C	6.27331100	1.98833000	1.04339600
O	8.31818600	0.44356200	-0.88673900
O	7.19806600	1.64889200	1.99021600
O	6.41856800	4.22154500	1.96359800
O	4.56449600	5.11694200	0.18413600
C	4.08887900	0.65642600	-1.73343200
O	2.74246600	0.84896300	-1.71524500
O	4.65045100	-0.18129500	-2.40847800
C	4.15459700	-1.45174400	1.16839800
O	3.72354100	-2.02220900	2.15547500
C	1.65411300	-2.56093600	-1.40562900
C	1.91352000	-0.23396000	-2.18237300
O	3.38519200	-0.84872000	0.24483100
O	1.04422000	-3.46472500	-0.49272500
O	2.10978700	0.52261000	2.38956300
O	9.48823600	-1.96029400	-0.59710100
O	8.19420500	-4.02052700	0.63421200
C	-7.51235600	0.84794500	-1.61052700
O	-7.60817500	0.56664600	-2.79168600
O	-7.44336000	2.12344200	-1.17840200
C	-7.49778300	3.13831300	-2.19987000
H	1.87639400	1.29674900	0.55433300
H	1.66932600	-1.70487900	1.00352200
H	-0.50590600	-1.75910000	-0.15718600
H	-0.29692000	1.40756000	1.70894200
H	-0.34746200	-0.34540300	1.85734000
H	-0.98686200	-0.41779500	-2.84790200
H	-0.65240900	-2.12351500	-2.65163200
H	-3.07987300	-1.76207900	-2.68993500
H	-2.51900600	-2.45336200	-1.18106900
H	-2.28029800	-0.66574700	0.88586500
H	-0.81252600	1.58557400	-2.01018100
H	0.61889900	1.83223700	-1.03135800
H	-0.91079600	2.51084300	-0.51781500

H	-4.93036100	2.00485400	1.57136500
H	-2.50960500	1.51214800	1.82434100
H	-2.76779200	2.27473300	0.27946600
H	-5.23015500	-2.44209700	-1.72769500
H	-5.52371400	-0.81822300	-2.30371600
H	-7.17525300	-2.28382400	-0.20796600
H	-7.63611100	-1.84226500	-1.84177500
H	-6.96752700	1.31776000	0.96945000
H	-9.46185300	0.77371700	0.01285900
H	-9.62061100	-0.69124500	-0.93993500
H	-9.13769300	-2.09460000	1.03379500
H	-10.45297800	-0.98878400	1.39057000
H	-6.50035000	-0.10503400	2.87473200
H	-6.76517700	-1.56617800	1.94917400
H	-4.15982000	1.52545400	-1.59441300
H	-2.63983000	1.19546500	-2.41749100
H	-4.08326300	0.33894700	-2.89598100
H	-8.93139600	1.63180000	2.04359500
H	-8.50033400	1.23242500	3.70769200
H	-10.12257300	0.86927700	3.10024300
H	-8.24587400	-1.15755100	4.47759900
H	-8.49503700	-2.49916100	3.34795900
H	-9.87161000	-1.51663100	3.87484800
H	-3.81695300	-1.78056100	1.57264000
H	-5.28017600	-2.53637800	0.97030800
H	-3.75285300	-2.89788000	0.19909300
H	5.75169400	-3.48689700	1.40112700
H	3.59972900	3.29940500	-1.42347300
H	7.75233800	1.20010800	-1.10786800
H	7.30753500	0.68533200	2.00729800
H	7.01044700	3.74051300	2.56305800
H	5.04897700	5.56047100	0.89841500
H	2.74721300	-2.66276400	-1.39498700
H	1.32021000	-2.75854600	-2.43356500
H	2.52023600	-0.85546000	-2.84327100
H	1.12697800	0.22725400	-2.77659800
H	1.43488900	-4.33695600	-0.63247800
H	2.06713400	-0.35201100	2.80420600
H	9.78714700	-1.15558500	-1.04945600
H	9.07946600	-3.95225100	0.24293900
H	-8.43438600	3.07069800	-2.75749700
H	-6.65955100	3.02977100	-2.89130200
H	-7.43556200	4.08892700	-1.67200800

Cartesian coordinates of the lowest-energy conformer of **4'oB** (**4'oB-1**) at the B3LYP/6-31G(d,p) level in pyridine (PCM).

C	-1.15850000	0.11761900	2.74122700
C	-2.01364900	-0.13011000	1.49169900
C	-1.49878700	-1.24638200	0.52914400
C	0.06252100	-1.05844600	0.33663200
C	0.94190700	-0.81968100	1.60621800
C	0.30309000	0.35592500	2.38922200
C	0.72953100	-2.10965100	-0.57289900
C	2.04539400	-1.54903000	-1.13124800
C	3.07178400	-1.14146000	-0.04280600
C	2.35827700	-0.32421500	1.09461400
C	1.00905700	-2.04917100	2.54075000
C	4.21769000	-0.19318200	-0.65867300
C	5.16067700	0.25513600	0.47719500
C	4.72713600	0.33001700	1.74288900
C	3.35548100	-0.04051100	2.23237300
C	5.00776900	-0.95252600	-1.76785000
C	6.36894900	-0.33832700	-2.12324200
C	7.28487200	-0.22621100	-0.88636400
C	6.60608500	0.67558200	0.18289100
C	8.70112800	0.31570300	-1.25782900
C	8.73247400	1.81306700	-1.58454900
C	8.13757600	2.69550400	-0.46470000
C	6.70881500	2.18371000	-0.16718000
C	3.71999500	-2.44161600	0.50632500
C	9.01963600	2.66369600	0.79998700
C	8.05351900	4.15129400	-0.95826900
C	3.63173500	1.08527400	-1.32862700

C	-3.83964200	2.54459600	1.16543600
C	-4.53119400	1.36762800	0.86402800
C	-5.37464300	1.26826200	-0.25943700
C	-5.48669800	2.39625600	-1.08622100
C	-4.80681600	3.58292800	-0.78955300
C	-3.98592400	3.65942200	0.33850200
C	-6.15654100	0.04604900	-0.64057800
C	-5.70616100	-0.89463100	-1.59666700
C	-6.57560100	-1.85029500	-2.13535300
C	-7.89333200	-1.93207100	-1.68795600
C	-8.31989700	-1.08631300	-0.65552700
C	-7.45534400	-0.11590400	-0.13829900
O	-6.25062400	2.44775800	-2.21729100
O	-7.94321700	0.63605800	0.89901200
O	-9.58177300	-1.25219000	-0.17252900
O	-8.73020100	-2.87341900	-2.20118700
C	-4.25791000	-1.04004100	-1.92355300
O	-3.52070200	-0.87993300	-0.79887300
O	-3.79048000	-1.34918000	-3.00248200
C	-4.49372500	0.18241300	1.76699900
O	-5.51502200	-0.25100300	2.28182900
C	-1.97085500	-2.66884700	0.97108800
C	-2.08975100	-1.01035600	-0.88824000
O	-3.34163900	-0.46443000	2.02909800
O	-2.05791000	-2.90079500	2.37295400
O	-1.63504000	1.28191700	3.42810500
O	-4.90985300	4.69593900	-1.57467100
O	-3.32518100	4.81232300	0.63321700
C	7.55503200	-1.61201100	-0.27898700
O	7.61951400	-1.86584600	0.90940100
O	7.80666100	-2.53824400	-1.22909700
C	8.16395300	-3.85003300	-0.75216900
H	-1.24951400	-0.75248900	3.39701400
H	-2.08592200	0.80871300	0.94042900
H	0.13646400	-0.10652400	-0.21247000
H	0.83852500	0.54501200	3.32417800
H	0.36667900	1.27788200	1.79551800
H	0.90359700	-3.04215900	-0.02596100
H	0.08945700	-2.37495100	-1.41933000
H	2.49928000	-2.28410800	-1.80524800
H	1.78010700	-0.68817800	-1.75235100
H	2.12601100	0.64672400	0.64394000
H	1.42558200	-2.93134400	2.05517800
H	0.01862100	-2.33329300	2.89575200
H	1.62935500	-1.83596900	3.41604800
H	5.42730600	0.65320800	2.51417600
H	2.97883500	0.76311300	2.87765800
H	3.45522200	-0.91414500	2.89185800
H	4.38909700	-1.00230900	-2.67102100
H	5.18791600	-1.98708300	-1.46797500
H	6.24477600	0.65586200	-2.56395300
H	6.85272400	-0.95493200	-2.88641000
H	7.15942700	0.52661900	1.11460500
H	9.38054700	0.11843500	-0.41979400
H	9.08445900	-0.26190300	-2.10616100
H	8.17645100	2.00084100	-2.51231000
H	9.76971000	2.11152900	-1.78455400
H	6.27970800	2.74933600	0.66908800
H	6.08533200	2.41465200	-1.03694800
H	4.60468000	-2.23803500	1.11225600
H	3.02509200	-3.01259000	1.11956500
H	4.02228800	-3.10340500	-0.30953400
H	10.02621900	3.03576400	0.57704600
H	9.12591500	1.66060600	1.22177000
H	8.59311400	3.30397400	1.58045600
H	7.61047100	4.80135900	-0.19504900
H	7.43964000	4.22917900	-1.86283000
H	9.04951300	4.54386800	-1.19402000
H	3.19593400	1.77774200	-0.60578500
H	4.41657500	1.63218400	-1.85398200
H	2.87219600	0.84384500	-2.07666500
H	-3.18523800	2.60606100	2.02769000
H	-6.21618800	-2.56178500	-2.86977500
H	-6.67500900	1.58774300	-2.36038600
H	-7.23893700	0.64790100	1.57938300
H	-9.68222900	-0.65650000	0.58930400

H	-9.57876800	-2.81249100	-1.73465400
H	-2.94747000	-2.85877000	0.51125100
H	-1.27703900	-3.41514100	0.57684300
H	-1.68948300	-0.09151700	-1.32863500
H	-1.86282400	-1.83761000	-1.56266600
H	-2.82065000	-2.39221200	2.68510800
H	-2.41116600	1.02096100	3.94214700
H	-5.50659600	4.49821600	-2.31326300
H	-3.54907100	5.47002600	-0.04409800
H	8.33200500	-4.45101700	-1.64482400
H	7.35471900	-4.27370200	-0.15364800
H	9.07160300	-3.80318600	-0.14649700
